# Taxonomic inventory and distributions of Chenopodiaceae (Amaranthaceae s.l.) in Orenburg Region, Russia

**DOI:** 10.3897/BDJ.12.e121541

**Published:** 2024-06-14

**Authors:** Alexander P. Sukhorukov, Maria A. Kushunina, Nina Yu. Stepanova, Olga G. Kalmykova, Yaroslav M. Golovanov, Alexander N. Sennikov

**Affiliations:** 1 M.V. Lomonosov Moscow State University, Moscow, Russia M.V. Lomonosov Moscow State University Moscow Russia; 2 Tomsk State University, Tomsk, Russia Tomsk State University Tomsk Russia; 3 Department of Plant Physiology, Biological Faculty, Lomonosov Moscow State University, 119234, Moscow, Russia Department of Plant Physiology, Biological Faculty, Lomonosov Moscow State University, 119234 Moscow Russia; 4 Tsitsin Main Botanical Garden, Moscow, Russia Tsitsin Main Botanical Garden Moscow Russia; 5 Institute of Steppe, Orenburg, Russia Institute of Steppe Orenburg Russia; 6 South Ural Botanical Garden-Institite, Ufa, Russia South Ural Botanical Garden-Institite Ufa Russia; 7 University of Helsinki, Helsinki, Finland University of Helsinki Helsinki Finland

**Keywords:** alien plants, arid lands, checklist, conservation, forest steppes, mapping, steppes

## Abstract

**Background:**

Orenburg Region is located in the South Urals, mostly in the steppe zone and is characterised by various landscapes suitable for many Chenopodiaceae. The species of Chenopodiaceae are present in all major plant communities (saline vegetation, steppes, on limestone, chalk and sand, and as degraded or ruderal communities). In the steppe zone, many native subshrubby species (*Atriplexcana*, *Caroxylonlaricinum*, *Suaedaphysophora*) playing a crucial role in semi-deserts (known as southern steppes in the recent Russian literature) located southwards of Orenburg Region are locally found, and several annuals (*Salicorniaperennans*, *Suaeda* spp.) are most common dominants in plant communities. Some typical semi-desert species (*Kalidiumfoliatum*, *Bassiahyssopifolia*, *Sodafoliosa*, *Spirobassiahirsuta*) are found in the easternmost part of the region.

**New information:**

We compiled a checklist of Chenopodiaceae in Orenburg Region, with two new records (*Chenopodiumvirgatum*, *Corispermumlaxiflorum*), based on our critical revision, comprehensive inventory of herbarium specimens and documented observations and field research. In total, we report 76 species in the Region, which is the third-highest number of the Chenopodiaceae species compared with other administrative territories of European Russia, North Caucasus and West Siberia. Alien and native taxa are distinguished. Zonal patterns of species distributions are confirmed. A preliminary conservation status is proposed for each native species. Three species are recommended for exclusion from the Red Data Book of Orenburg Region: *Petrosimoniatriandra* (because of its extensive distribution), *Kalidiumfoliatum* and *Anabasissalsa* (because of the lack of actual threat to their populations). *Arthrophytumlehmannianum* and *Salsolarosacea* are considered threatened (Vulnerable) because of their restricted occurrence and population size and because their localities are under anthropogenic pressure. *Atriplexhortensis*, *Atriplexrosea*, *Chenopodiumacuminatum*, *C.karoi*, *C.praetericola*, *C.vulvaria*, *Climacopteraaffinis*, *C.crassa*, *Halimocnemiskarelinii*, *Salsolapaulsenii* and *Xylosalsolaarbuscula* are excluded from the checklist, based on various reasons as discussed in the paper. Point distribution maps are provided for each species. *Agriophyllumpungens* (Vahl) Link is accepted as the correct authorship instead of "M.Bieb. ex C.A.Mey."

## Introduction

**Figure 1.**
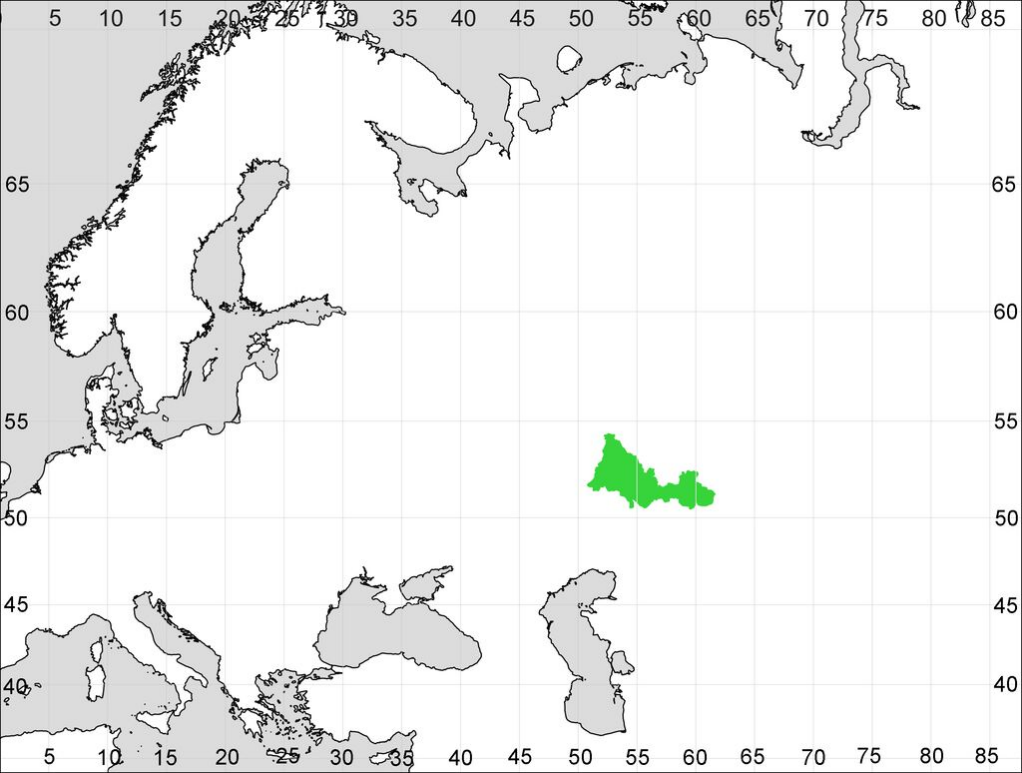
Location of Orenburg Region in Eurasia.

Orenburg Region is a first-level administrative territory (federal subject) of the Russian Federation (Fig. 1). It covers the southern extensions of the Ural Mountains (Guberlya Mts.) and adjacent portions of the East European Plain in the west and the West Siberian Plain in the east, which are mostly hilly; it also includes tiny portions of the Caspian Lowlands in the south-west. Orenburg Region is divided by the border between Europe and Asia, tentatively running along the watershed of the Urals (Shahgedanova et al. 2002). At the Russian scale, it is a relatively small administrative territory that extends to slightly less than 124,000 km^2^, thus ranking 39 in the list of federal subjects of Russia by area.

The family Chenopodiaceae Vent (Amaranthaceae Juss. s.l.) is most abundant in arid zones of Eurasia (Wulf 1943, Zhu 1995, Akhani et al. 1997). The majority of Chenopodiaceae in the Eurasian steppe zone, including Orenburg Region, are confined to open plant communities, especially on saline substrates (Prokopyeva et al. 2021). This type of vegetation is characterised by a wide variability in response of plants to the local conditions (e.g. Kamenetskaya (1952), Gordeeva and Larin (1965), Saiz et al. (2018)). Open vegetation types, most commonly steppes, are threatened due to extensive agricultural use of the steppe zone in the recent times (Török et al. 2016, Tsiripidis 2016, Liu and Wang 2021). In Orenburg Region, five species of Chenopodiaceae (*Anabasiscretacea* Pall., *A.salsa* (Ledeb.) Benth. ex Volkens, *Kalidiumfoliatum* (Pall.) Moq., *Nanophytonerinaceum* (Pall.) Bunge, *Petrosimoniatriandra* (Pall.) Simonk.) were included in the Red Data Book of Orenburg Region (Belov 2019).

The Chenopodiaceae of Orenburg Region were not specifically studied between the late 18^th^ century and the early 20^th^ century. Although the territory of present-day Orenburg Region was effectively included in the Russian Empire by building a chain of fortifications along the Southern Urals in 1730s-1740s, it was rarely visited by botanists due to its remote position at the very limit of the Empire and the ongoing battles with the nomadic population. Fenzl (1851) provided the first records of seven most common species of Chenopodiaceae in the territory, based on the collections of P.S. Pallas from the 18^th^ century. Among the early plant hunters, G.S. Karelin and Antonov (first name unknown) collected some specimens from "Orenburg", but their annotations are considered unreliable (e.g. Knjazev (2009)) because their specimens were most likely at least partly collected in present-day Kazakhstan. The first reliable scientific collections of Chenopodiaceae of Orenburg Region were assembled by Yu. Schell, D.I. Litvinov and other people who made botanical excursions to the Southern Urals and neighbouring steppes in the late 19^th^ and early 20^th^ centuries, but a large-scale botanical exploration of the terrirory started following the ground-breaking description and analysis of the vegetation of south-eastern European Russia and north-western Kazakhstan published by Krascheninnikov (1925).

Iljin (1930) critically summarised the knowledge on the diversity of Chenopodiaceae in south-eastern European Russia, including parts of Orenburg Region, based on the herbarium collections which are partly at LE, but partly no longer available. He developed an original taxonomy of the family and provided an analysis of its diversity and distributions according to the vegetation zones delimited by Krascheninnikov (1925). This treatment was a milestone in taxonomic studies of Chenopodiaceae in the arid zone of Eurasia.

Further accumulation of herbarium collections from Orenburg Region was largely connected with its geobotanical exploration, especially for the Soviet development of steppic territories into arable lands (Ryabinina 1998).

The multivolume Flora of Eastern Europe belongs to the most siginificant floristic overviews of the late 20^th^ century (Sennikov and Geltman 2005). It provided taxonomic and distributional data for the territories situated west from the Ural watershed, i.e. including the western part of Orenburg Region. This work (Gusev 1996, Medvedeva 1996, Mosyakin 1996, Tzvelev 1996) gives a broad-scale overview of the taxonomy and distributions of Chenopodiaceae of Eastern Europe. Due to the brief format of this work, it is impossible to separate the data on Orenburg Region or even ascertain the presence of some species in the region because individual administrative territories were not specified. In particular, many species (e.g. *Chenopodiumvulvaria*) were treated in this work as occurring in the whole territory of the Transvolga Floristic Region, including Orenburg Region, which have never been reported in more detailed surveys and supported by herbarium specimens or other documented observations. *Atriplexrosea* was also included in this work, but treated as absent in Orenburg Region by Sukhorukov et al. (2022) .

The modern period in floristic works was started with Z.N. Ryabinina, who studied the flora and vegetation of Orenburg Region towards a floristic synopsis with detailed distributions (Ryabinina 1998), inventories of protected (Ryabinina 1995), arboreous (Ryabinina and Vyelmovsky 1999), riparian and aquatic plants (Ryabinina and Rachenkova 2008) and the steppic vegetation (Ryabinina 2003). Her floristic works were based on extensive field observations, but practically no voucher specimens were preserved, thus not allowing the verification of the distributional data. During the same period, extensive herbarium collections were made by field botanists from Ekaterinburg, M.S. Kniazev and P.V. Kulikov. As a result of this collection activity, a new treatment of Chenopodiaceae was published (Knjazev 2009), with supplements based on the later fieldwork (Golovanov et al. 2018).

In 2020-2021, the first author in cooperation with other botanists carried out fieldwork in Orenburg Region, specifically targeting the family Chenopodiaceae. During our field and herbarium studies, we noted that the taxonomic composition of the family, species distributions, their roles in plant communities and possible conservation need to be further studied in details or significantly improved. Some important data (recorded by Ryabinina) cannot be authenticated properly, whereas many older herbarium collections originated from Orenburg Region in the 1930s and scattered in local herbaria (e.g. MOSP, PKM) have never been revised and included in floristic and conservational work. Other important treatments either utilised the information accumulated in the largest Russian herbaria (LE and MW) or were based on the locally collected and preserved information. To date, no attempt has been made to summarise and verify the information accumulated in all the collections, in order to produce a reliable treatment of Chenopodiaceae in Orenburg Region and its analysis in the context of the zonal patterns of Eurasian steppes.

The aims of the present study are: (1) to include new records and more information about species distributions of Chenopodiaceae in Orenburg Region, based on a new critical revision of the herbarium material in all available collections and our own field work, as well as comparisons of the species diversity between Orenburg Region and other territories in the steppic zone of Eastern Europe, North Caucasus and West Siberia; (2) to revise distributions and produce point maps for all species, showing their distrubition patterns; and (3) to re-evaluate their conservation status in provisional IUCN assessments.

## Materials and methods

### Study area

This study is strictly limited by the administrative borders of Orenburg Region, Russian Federation. Bounding coordinates: south-west 50.506° N, 50.768° E; north-east 54.374° N, 61.066° E.

**Figure 2.**
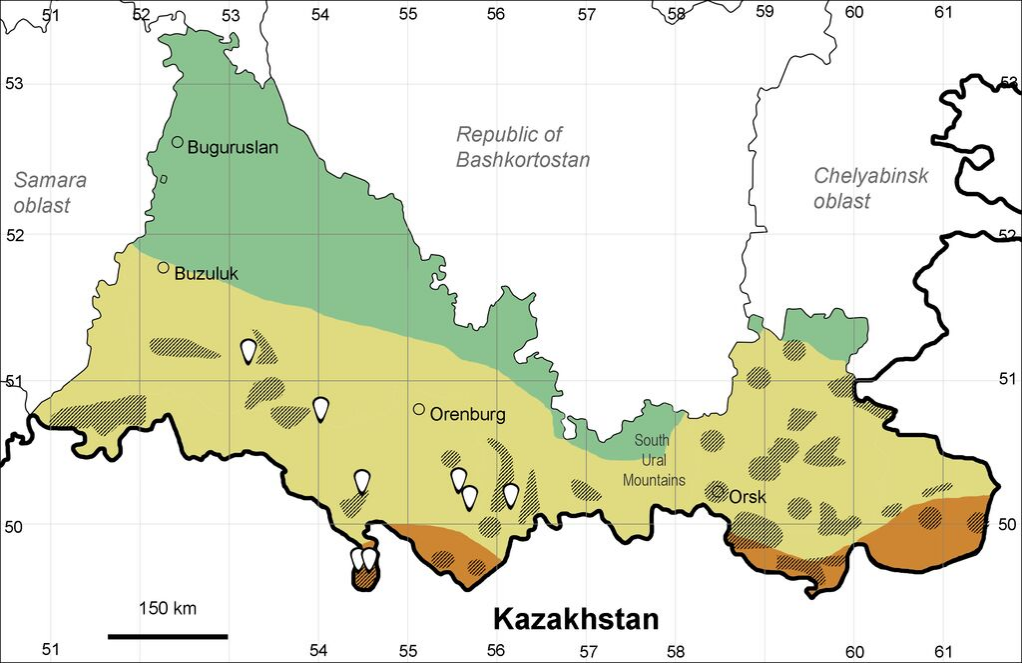
Zonal subdivision and azonal substrates of Orenburg Region. Green: forest steppe; straw: northern steppe; brown: true steppe. Hatching: saline lands; white symbols: calcareous outcrops. Sources: Ogureeva (1999), corrected after Safronova et al. (2020) and Safronova (2021).

Orenburg Region lies in the forest-steppe and steppe zones (Fig. 2) but includes a variety of intrazonal landscapes, i.e. limestones, sands and saline lands (Ryabinina 1998). In the north-western part, the steppe vegetation is interspersed by various forest types including broad-leaved and pine forests. The steppe zone is subdivided into two belts, which remarkably differ in their water regime and vegetation: the northern steppe, a more humid area of *Stipa, Festuca* and forb steppes and the true steppe, a drier area of *Stipa* and *Festuca* steppes with a considerable participation of semi-desert plants and a lesser influence of forbs (Chibilyov 2002). The flora and vegetation are remarkably different in the European and Siberian parts of the Region, depending on the influence of the European and Central Asian floras (Krascheninnikov 1925). This difference is based on a higher continentality of the Asian steppes (Chibilyov 2002). According to recent studies (Safronova 2021), Artemisieta santonicae and Artemisieta lerchianae predominate in the Transvolga Region, whereas Artemisieta nitrosae prevails in the Transural Region. This difference may also be caused by a greater aridity and continentality of the Transuralian territories, because the Ural Mountains effectively act as a barrier for the oceanic climate (Shahgedanova et al. 2002).

The exact delimitation of the steppe zone in Orenburg Region has been debated and variously represented on vegetation maps, of which the most recent was published by Ogureeva (1999). We accept the subdivisions and limits of the steppe zone made in that work and corrected on the basis of recent field studies (Safronova et al. 2020, Safronova 2021).

### Taxonomic scope

In this work, we include native and alien (spontaneous) taxa of Chenopodiaceae occurring in the study area. Alien taxa are considered spontaneous if they occur without direct human assistance. Unlike in previous treatments (Iljin 1936, Ryabinina 1998, Knjazev 2009), cultivated taxa (e.g. *Beta vulgaris* L.) are excluded if they do not occur spontaneously outside the places of their introduction or if they do not persist in places of introduction when the cultivation activities have ceased for a considerably long time. Alien taxa are subdivided into casual and established (naturalised) aliens, based on their long-term persistence (Pyšek et al. 2004).

### Taxonomy

Our checklist is organised taxonomically according to the following system (genera classified in subfamilies and tribes).

Chenopodioideae Burnett: Anserineae Dumort. (*Blitum* L.), Dysphanieae Pax (*Teloxys* Moq., *Dysphania* R.Br.), Chenopodieae Dumort.(*Atriplex* L., *Chenopodium* L., *Chenopodiastrum* S.Fuentes, Uotila & Borsch, *Halimione* Aellen, *Lipandra* Moq., *Oxybasis* Kar. & Kir.), Axyrideae G.Kadereit & Sukhor. (*Axyris* L., *Ceratocarpus* L., *Krascheninnikovia* Gueldenst.).

Corispermoideae Raf.: *Agriophyllum* Link, *Corispermum* L.

Salicornioideae Kostel.: *Halocnemum* M.Bieb., *Kalidium* Moq., *Salicornia* L.

Suaedoideae Ulbr.: *Suaeda* L.

Camphorosmoideae A.J.Scott: *Bassia* All., *Camphorosma* L., *Sedobassia* Freitag & G.Kadereit, *Spirobassia* Freitag & G.Kadereit.

Salsoloideae Raf.: Caroxyleae Akhani & Roalson (*Caroxylon* Thunb., *Nanophyton* Less., *Ofaiston* Raf., *Petrosimonia* Bunge, *Pyankovia* Akhani & Roalson), Salsoleae C.A.Mey. (*Anabasis* L., *Arthrophytum* Schrenk, *Halogeton* Ledeb., *Salsola* L., *Soda* Fourr.).

Phylogenetically, the family Chenopodiaceae is accepted and circumscribed as a part of the Amaranthaceae s.l. alliance (e.g. Kadereit et al. (2003), Ogundipe and Chase (2009)). The infrafamilial relationships and generic delimitations were greatly improved and specified by the recent detailed studies based on molecular phylogeny: Hohmann et al. (2006) in Betoideae; Kadereit and Freitag (2011) in Camphorosmoideae, Kadereit et al. (2010), Fuentes-Bazan et al. (2012a), Fuentes-Bazan et al. (2012b), Sukhorukov et al. (2018), Uotila et al. (2021) in Chenopodioideae; Shepherd et al. (2005), Kadereit et al. (2006) in Salicornioideae; Akhani et al. (2007), Wen et al. (2010) in Salsoloideae and Schütze et al. (2003) in Suaedoideae. *Polycnemum* L., traditionally included in Chenopodiaceae s.str. (Ryabinina 1998, Knjazev 2009), is excluded from the checklist because it was placed in Amaranthaceae s.str. in molecular phylogenetic studies (Kadereit et al. 2003).

For generic delimitations and species classifications, we accepted the latest revisions of Chenopodieae (Fuentes-Bazan et al. 2012b), Dysphanieae (Uotila et al. 2021), some *Chenopodium* (Uotila and Lomonosova 2016, Lomonosova and Uotila 2022) and *Suaeda* (Lomonosova et al. 2008). The general taxonomic framework was proposed by Sukhorukov (2014).

### Nomenclature

We verified and corrected the nomenclature of accepted names and their synonyms according to their protologues and the current rules of botanical nomenclature. References to the places of valid publication are provided for each name at the rank of species and below. By accepted species names, links are provided to the corresponding entries in IPNI, although the nomenclatural and bibliographic data in these entries are not necessarily full and correct. The most important synonyms are given, especially those used in the previous accounts (Iljin 1930, Gusev 1996, Medvedeva 1996, Mosyakin 1996, Tzvelev 1996, Ryabinina 1998, Knjazev 2009). Typifications are not cited as deemed inappropriate in a regional inventory, but synonyms are organised according to their types.

### Distributions

Species distributions in Orenburg Region are indicated according to the current first-level administrative subdivisions (districts, largest towns). District names in English are derived from the names of their respective administrative centres.

Species distributions in Orenburg Region are compiled and visualised on point maps strictly according to the specimens examined and documented observations. Literature data (Ryabinina 1998, Knjazev 2009) may differ in details because of undocumented observations taken into account; such differences are mentioned or discussed case by case. Species distributions outside Orenburg Region follow Sukhorukov (2014), with updates.

Doubtful and unconfirmed records from the 19^th^ century were excluded from the checklist and added to the list of rejected records, whereas more certain records of *Kalidiumcaspicum* and *Corispermumlaxiflorum* originating from the early 20^th^ century were retained in the main checklist. These two species may be recollected like *Petrosimonabrachyphylla*; the first record of this species originated in the 1930s and was confirmed by three recent gatherings in this study.

We compared the number of species in Orenburg Region with those in other regions of southern European Russia (Bashkortostan, Chelyabinsk Region, Rostov Region, Samara Region, Saratov Region, Volgograd Region), West Siberia (Kurgan Region) and East Caucasus (Dagestan), from which more complete data were available. These administrative units are fully or partially located in the steppe zone, thus sharing many suitable habitats. The species numbers of Chenopodiaceae in these territories were taken from earlier accounts: Murtazaliev (2009) for Dagestan, Naumenko (2008) for Kurgan Region, Sukhorukov (2014) with additions (Muldashev et al. 2017; Sukhorukov, in prep.) for European Russia.

### Material

We traced and revised almost all specimens of the Chenopodiaceae ever collected from Orenburg Region. Specimens kept in the following herbaria were studied (acronyms according to Thiers (2024)): LE, LECB, MHA, MOSM, MOSP, MW (GBIF 2023b), MWG, NNSU, ORIS, PKM, PVB, SARAT, SVER (as images), TLT, UFA (as images) and Herbarium of the Orenburg Pedagogical University (not registered in Index Herbariorum). Verified data from iNaturalist (GBIF 2023a) were also included. Our own field studies (2000-2022) were undertaken in various districts of Orenburg Region and specimens were deposited to BR, K, LE, MW, MHA and ORIS. All specimens examined and documented observations were provided as an occurrence dataset in the Supplement (Suppl. material 1).

Species occurrences were georeferenced by the authors and mapped using SimpleMappr online tool (Shorthouse 2010). For the most widespread species, we have mapped all records and additionally hatched the districts where the species are clearly present even in the absence of current records.

### Conservation assessments

Regional conservation status of each native species was preliminarily assessed using the IUCN Red List Categories and Criteria (IUCN 2012) according to our knowledge about their distributions and projected reductions of population size (IUCN 2012b). Area of occupancy (AOO) and extent of occurrence (EOO) were calculated with the Geospatial Conservation Assessment Tool (GeoCAT: Bachman et al. (2011)).

## Checklists

### Chenopodiaceae of Orenburg Region

#### Blitum virgatum

L.

8E42B68B-0BF3-535D-855B-05A4EFCAB178

urn:lsid:ipni.org:names:164548-1

***Blitumvirgatum*** L., Sp. Pl. 1: 4 (1753) ≡ *Morocarposfoliosus* Moench, Methodus: 342 (1794), nom. illeg. ≡ *Chenopodiumfoliosum* Asch., Fl. Prov. Brandenburg 1: 572 (1864).

##### Native status

Native.

##### Conservation status

Least Concern (LC).

EOO 41,345 km^2^, AOO 48 km^2^. Not rare, no reduction or decline.

##### Distribution

**Figure 3.**
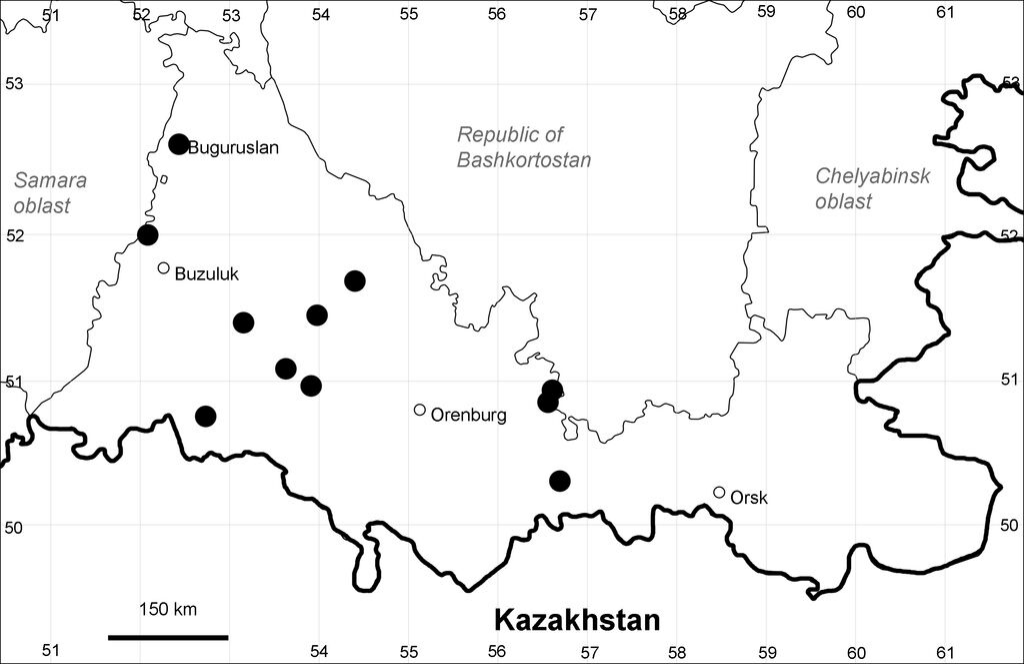
Distribution map of *Blitumvirgatum* (confirmed occurrences).

Orenburg Region (western districts): Alexandrovka, Belyaevka, Buguruslan, Buzuluk, Novosergievka, Saraktash, Sorochinsk, Tashla Districts (Fig. 3). Eastern limit of the European distribution fragment.

Previous reports: Iljin (1930), Ryabinina (1998), Knjazev (2009).

The species occurs on limestone, gravels, also in human-disturbed sites. It was found in scattered occurrences in the Region. Ryabinina (1998) and Knjazev (2009) assumed that the species distribution is broader than it is currently confirmed.

The species is distributed as native in the Mediterranean, West and Central Asia, Iran and the Himalayas. It is known as alien in North America, rest of Europe, South Africa and temperate Asia.

#### Teloxys aristata

(L.) Moq.

EA60E4D5-CB84-538B-967E-6E1BE00B01E0

urn:lsid:ipni.org:names:1113636-2

***Teloxysaristata*** (L.) Moq., Ann. Sci. Nat. Bot., ser. 2(1): 289 (1834) ≡ *Chenopodiumaristatum* L., Sp. Pl. 1: 221 (1753) ≡ *Dysphaniaaristata* (L.) Mosyakin & Clemants, Ukr. Bot. Zhurn. 59(4): 383 (2002).

##### Native status

Alien. Presumably casual.

##### Conservation status

Not applicable.

##### Distribution

**Figure 4.**
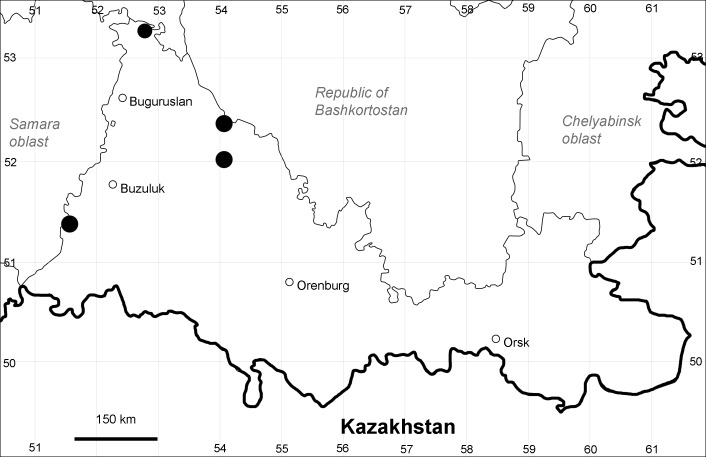
Distribution map of *Teloxysaristata*.

Orenburg Region (western districts): Kurmanaevka, Ponomaryovka, Severnoe Districts (Fig. 4).

Previous reports: Ryabinina (1998), Knjazev (2009).

The species occurs on sands, also in human-disturbed sites. Rare in the Region.

The species is distributed as native in Central Asia and the Himalayas. It is known as alien in North America, Europe, North and East Asia.

#### Dysphania botrys

(L.) Mosyakin & Clemants

6DF3F0CC-B007-5EA9-BEB1-458C6DF2C082

urn:lsid:ipni.org:names:1076147-2

***Dysphaniabotrys*** (L.) Mosyakin & Clemants, Ukr. Bot. Zhurn. 59(4): 383 (2002) ≡ *Chenopodiumbotrys* L., Sp. Pl. 1: 219 (1753).

##### Native status

Native.

##### Conservation status

Data Deficient (DD).

A single occurrence has been confirmed, but the species distribution is presubambly broader (Ryabinina 1998, Knjazev 2009). For this reason, a threatened category has not been assigned.

##### Distribution

**Figure 5.**
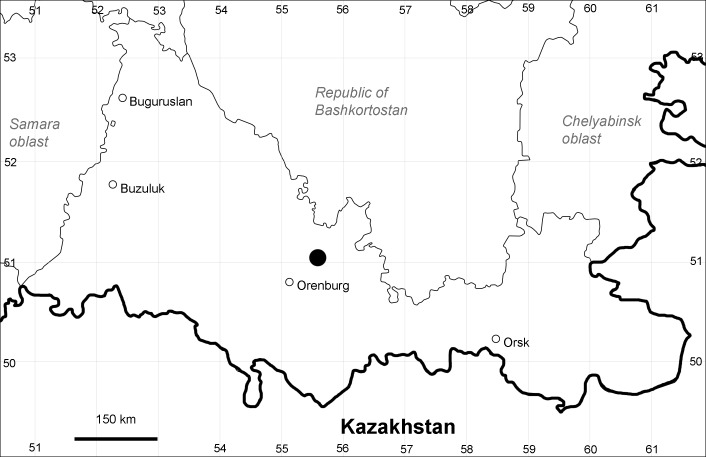
Distribution map of *Dysphaniabotrys* (confirmed occurrence).

Orenburg Region: Sakmara District (Fig. 5).

Previous reports: Ryabinina (1998), Knjazev (2009).

The species occurs on limestone, screes, also in human-disturbed sites. Only one collection is known, dated 1980s, although Ryabinina (1998) and Knjazev (2009) considered the species to occur in several districts.

The species is distributed as native in arid and semi-arid parts of Eurasia (predominantly the Irano-Turanian and Mediterranean Regions). It is known as alien in Northern and Central Europe, Northern Africa and North America.

#### Atriplex aucheri

Moq.

731AC785-2D48-58D2-A008-9B4B08523437

urn:lsid:ipni.org:names:163662-1

***Atriplexaucheri*** Moq., Сhenop. Monogr. Enum.: 51 (1840).
= *Atriplexamblyostegia* Turcz., Bull. Soc. Nat. Mosc. 25(2): 416 (1852).
= Atriplexnitenssubsp.desertorum Iljin, Bull. Jard. Bot. Princ. l’URSS 26: 414 (1927).

##### Native status

Native.

##### Conservation status

Data Deficient (DD).

A threatened category has not been assigned due to the absence of recent collections.

##### Distribution

**Figure 6.**
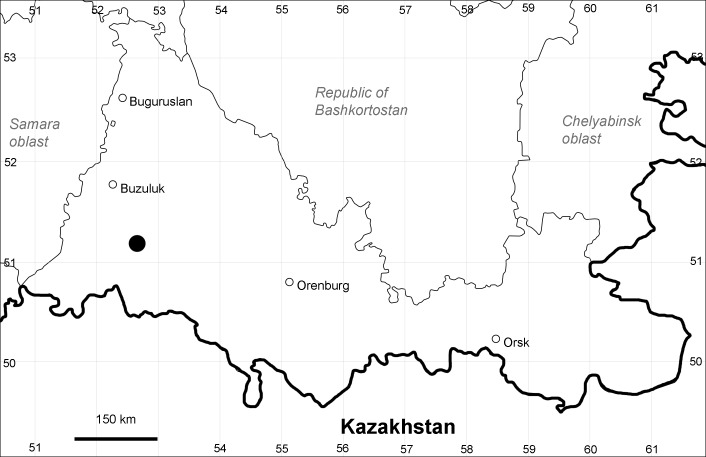
Distribution map of *Atriplexaucheri*.

Orenburg Region: Totskoe District (Fig. 6).

Previous reports: Knjazev (2009).

The species occurs on saline substrates, also in human-disturbed sites. Only one collection, dated 1930s.

The species is distributed in the Black Sea area (westwards to Bulgaria), European Russia (south-eastern part), the Caucasus, Central and West Asia, north-western China.

#### Atriplex cana

Ledeb.

4762E165-4636-5AED-8204-6211213F3CEC

urn:lsid:ipni.org:names:163698-1

***Atriplexcana*** Ledeb., Ic. Pl. Fl. Ross. 1: 11 (1829) ≡ *Sukhorukoviacana* (Ledeb.) Vasjukov, Botanika (Minsk) 44: 119 (2015).

##### Native status

Native.

##### Conservation status

Least Concern (LC).

EOO 71,750 km^2^, AOO 80 km^2^. Common, no reduction or decline.

##### Distribution

**Figure 7.**
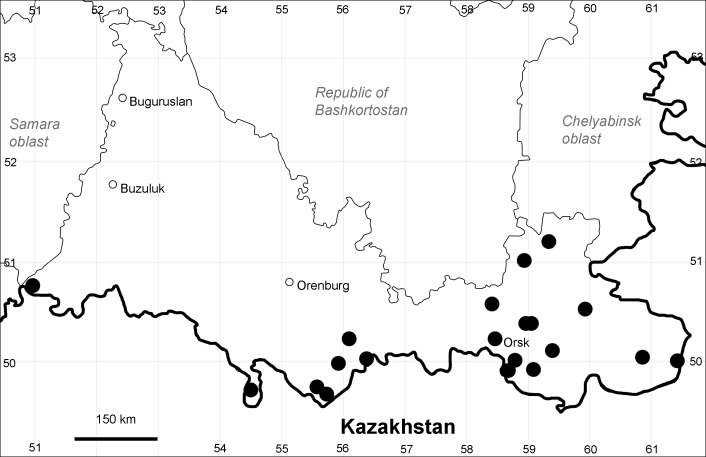
Distribution map of *Atriplexcana*.

Orenburg Region (eastern and southern districts): Adamovka, Akbulak, Belyaevka, Dombarovka, Gai, Kvarkeno, Novoorsk, Pervomaiskii, Sol'-Iletsk, Svetlyi, Yasnyi Districts, Orsk Town (Fig. 7).

Previous reports: Iljin (1930), Ryabinina (1998), Knjazev (2009).

The species occurs on saline substrates, in petrophytic steppes. It is frequent in the south and east of the Region.

The species is distributed in Eastern Europe (south-eastern part), southern Siberia, the Caucasus, Central Asia (Kazakhstan, northern Uzbekistan), western Mongolia, north-western China.

#### Atriplex intracontinentalis

Sukhor.

7D370B0B-9D96-52D4-A866-A92D93B90452

urn:lsid:ipni.org:names:77080816-1

***Atriplexintracontinentalis*** Sukhor., Ann. Naturhist. Mus. Wien 107 B: 349 (2006).
– *Atriplexlittoralis* auct.: Iljin (1930).
– *Atriplexlaevis* auct.: Medvedeva (1996).

##### Native status

Native.

##### Conservation status

Least Concern (LC).

EOO 79,880 km^2^, AOO 56 km^2^. Common, no reduction or decline.

##### Distribution

**Figure 8.**
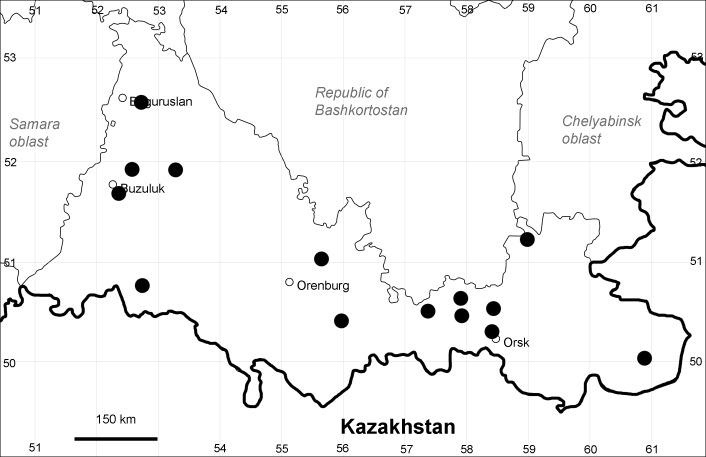
Distribution map of *Atriplexintracontinentalis*.

Orenburg Region (whole territory): Asekeevo, Belyaevka, Buzuluk, Gai, Krasnaya Gvardia, Kuvandyk, Kvarkeno, Sakmara, Svetlyi, Tashla Districts, Orsk Town (Fig. 8).

Previous reports: Ryabinina (1998), as *A.littoralis*; Sukhorukov (2006), Knjazev (2009).

The species occurs on saline substrates. It is frequent in the Region.

The species is distributed in Central and Eastern Europe, southern Siberia, Kazakhstan.

#### Atriplex laevis

Ledeb.

931B86CA-55CA-5BDE-8EBA-A97DDF092FD5

urn:lsid:ipni.org:names:60448166-2

***Atriplexlaevis*** Ledeb., Ic. Pl. Fl. Ross. 1: 10 (1829).

##### Native status

Native. Knjazev (2009) noted that this species occurs on saline substrates, but also spreads along railway embankments.

##### Conservation status

Least Concern (LC).

EOO 520 km^2^, AOO 24 km^2^. Despite the apparently restricted occurrence at the very margin of the distribution area, the species demonstrates no tendency in reduction of its distribution or in decline of its populations. Moreover, it expands to secondary habitats (Knjazev 2009).

##### Distribution

**Figure 9.**
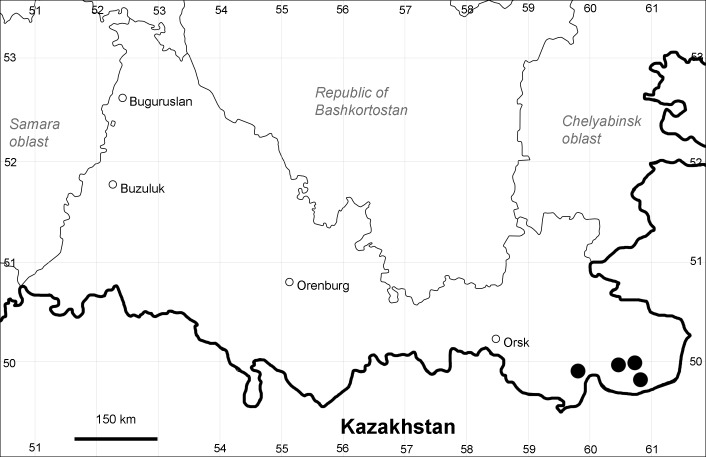
Distribution map of *Atriplexlaevis*.

Orenburg Region (south-eastern districts): Svetlyi, Yasnyi Districts (Fig. 9). North-western limit of the native species distribution.

Previous reports: Ryabinina (1998), Knjazev (2009).

The species occurs on saline lands and sands, along waterbodies, also in human-disturbed sites. It is rare in the Region.

The species is distributed as native in southern Siberia, Mongolia, north-western and northern China, northern and eastern Kazakhstan. It is alien in Fennoscandia, Eastern Europe, West Asia (Turkey), Iran, Syria, Caucasus (Armenia), East Asia (Korea, Japan), Central Asia (western Kazakhstan, Tajikistan).

#### Atriplex micrantha

Ledeb.

D9B224EC-F381-5AC5-AEBA-65D06B698050

urn:lsid:ipni.org:names:60448164-2

***Atriplexmicrantha*** Ledeb., Icon. Pl. Fl. Ross. 1: 11 (1829).
= *Atriplexheterosperma* Bunge, Beitr. Fl. Russl.: 272 (1852).

##### Native status

Native.

##### Conservation status

Least Concern (LC).

EOO 67,635 km^2^, AOO 44 km^2^. This species is not rare and has no tendency for declining.

##### Distribution

**Figure 10.**
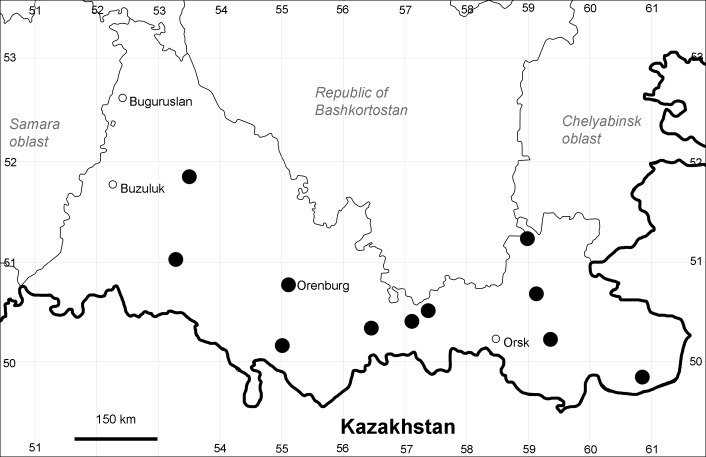
Distribution map of *Atriplexmicrantha* (confirmed occurrences).

Orenburg Region: Belyaevka, Krasnaya Gvardia, Kuvandyk, Kvarkeno, Novoorsk, Novosergievka, Sol'-Iletsk, Svetlyi, Yasnyi Districts, Orenburg City (Fig. 10). The species is very common in Orenburg Region and its occurrence in other districts is suggested (Ryabinina 1998, Knjazev 2009).

Previous reports: Iljin (1930), Ryabinina (1998), Knjazev (2009).

The species occurs on saline substrates, sometimes as a weed. It was found sporadically mostly in the southern part of the Region.

The species is distributed as native in Central Asia, north-western China, southern Siberia, in steppes and deserts of southern Eastern Europe. It is known as alien in Western, Central and Northern Europe (as ephemerophyte in Fennoscandia), West Asia, East Asia (Russian Far East), temperate North and South America.

#### Atriplex oblongifolia

Waldst. & Kit.

FE09262E-A088-55A9-8D74-EBC50C82E4EF

urn:lsid:ipni.org:names:164012-1

***Atriplexoblongifolia*** Waldst. & Kit., Descr. Icon. Pl. Rar. Hung. 3: 278 (1812).

##### Native status

Native.

##### Conservation status

Least Concern (LC).

EOO 34,900 km^2^, AOO 36 km^2^. This species is not rare and has no tendency for declining.

##### Distribution

**Figure 11.**
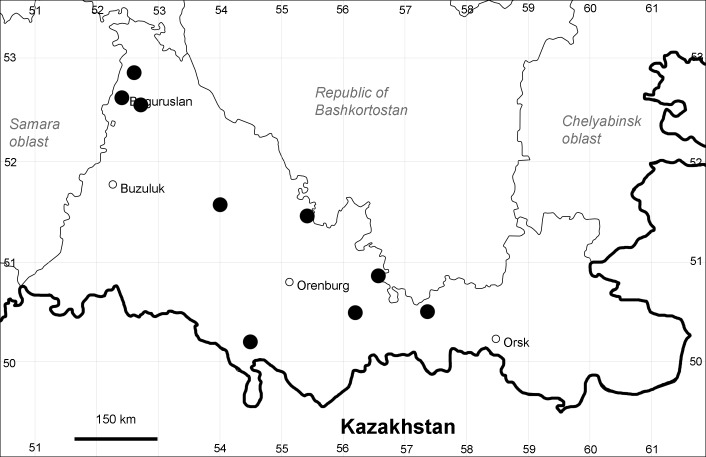
Distribution map of *Atriplexoblongifolia*.

Orenburg Region (western districts): Asekeevo, Belyaevka, Buguruslan, Kuvandyk, Novosergievka, Oktyabrskoe, Saraktash, Sol'-Iletsk Districts (Fig. 11).

Previous reports: Iljin (1930), Ryabinina (1998), Knjazev (2009).

The species occurs in steppes, meadows, on saline lands, also found in human-disturbed sites. It is rather frequent in the Region.

The species is distributed as native in Eastern and Southern Europe, Central Asia (except Turkmenistan and Tajikistan), south-western Siberia, north-western China (western part of Xinjiang). It is known as alien in North America, Central, Western and Northern Europe.

#### Atriplex patens

(Litv.) Iljin

38C40008-C27B-570E-9554-262BE38CAE11

urn:lsid:ipni.org:names:164043-1

***Atriplexpatens*** (Litv.) Iljin, Bull. Jard. Bot. Princ. URSS 26(4): 415 (1927) ≡ Atriplexlittoralisvar.patens Litv., Sched. Herb. Fl. Ross. 5: 12 (1905).
– *Atriplexcrassifolia* auct.: Knjazev (2009).

##### Native status

Native.

##### Conservation status

Least Concern (LC).

EOO 21,000 km^2^, AOO 32 km^2^. This species is common in the south of Orenburg Region and has no tendency for declining.

##### Distribution

**Figure 12.**
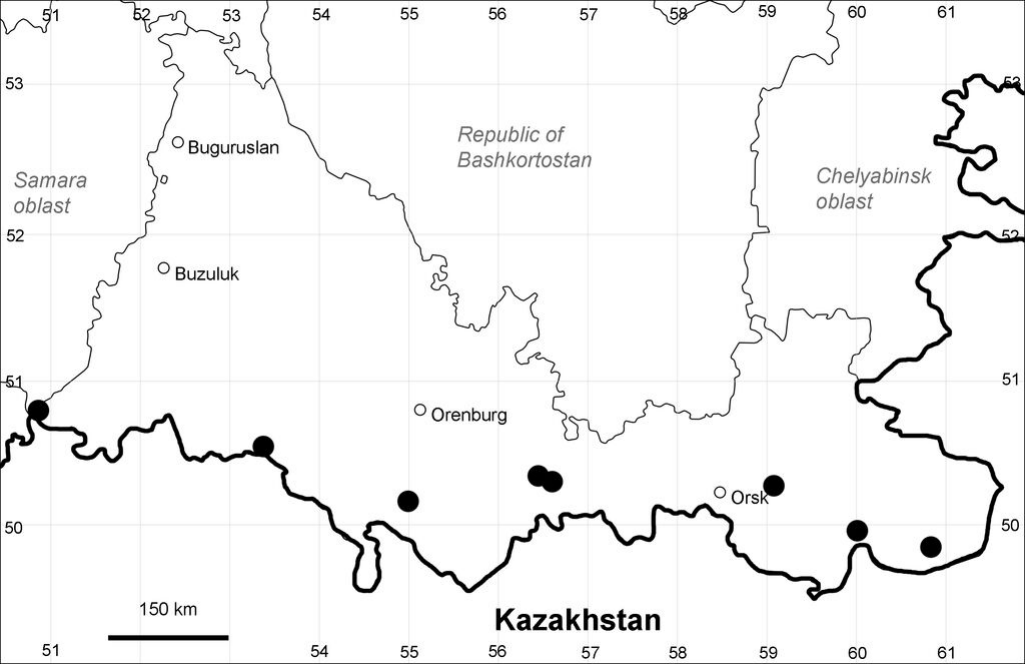
Distribution map of *Atriplexpatens* (confirmed occurrences).

Orenburg Region (southern districts): Belyaevka, Ilek, Novoorsk, Pervomaiskii, Sol'-Iletsk, Svetlyi, Yasnyi Districts (Fig. 12). Northern limit of the native species distribution.

Previous reports: Iljin (1930), Ryabinina (1998), Knjazev (2009).

The species occurs on saline substrates. It is common in the southern part of the Region.

The species is distributed as native in Eastern Europe, North Caucasus, Central Asia (northern part), Siberia. It is known as alien in the forest zone of European Russia and the Russian Far East.

#### Atriplex patula

L.

EF32AB34-FD98-52F4-8D98-0E75B8345E11

urn:lsid:ipni.org:names:26419-2

***Atriplexpatula*** L., Sp. Pl. 1: 1053 (1753).

##### Native status

Alien. Based on the scattered distribution and local occurrence, the residence status is likely casual (ephemerous or locally persisting). The earliest record (Orenburg City) is dated 1870s.

##### Conservation status

Not applicable.

##### Distribution

**Figure 13.**
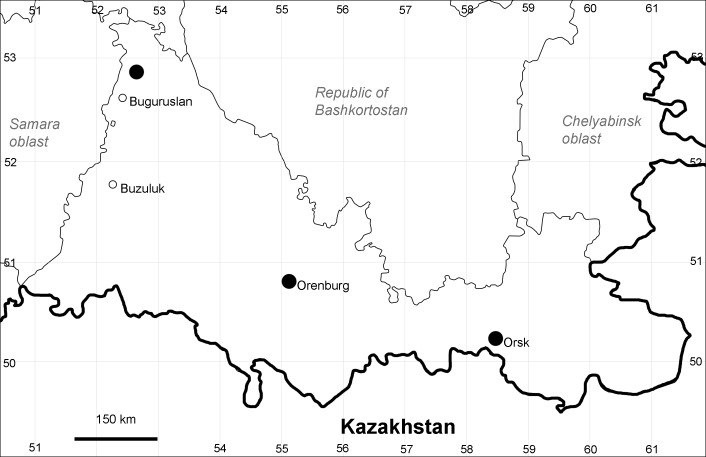
Distribution map of *Atriplexpatula*.

Orenburg Region: Buguruslan District, Orsk Town, Orenburg City (Fig. 13).

Previous reports: Iljin (1930), Ryabinina (1998), Knjazev (2009).

The species is found in ruderal habitats in populated places, mostly in direct connection with transport discharge areas.

The native distribution area is uncertain, but may cover Southern Europe and Mediterranean West Africa. The species is alien in temperate Eurasia, North and South America.

#### Atriplex prostrata

Boucher ex DC.

CD24E9BB-9DAF-5187-B16F-0CD50CDBC840

urn:lsid:ipni.org:names:60448217-2

***Atriplexprostrata*** Boucher ex DC. in Lam. & DC., Fl. Franc., ed. 3, 3: 387 (1805).
= *Atriplexlatifolia* Wahlenb., Svensk Bot. 9: pl. 628 (1824).
– *Atriplexcalotheca* auct.: Ryabinina (1998).
– *Atriplexhastata* auct.: Iljin (1930).

##### Native status

Native.

##### Conservation status

Least Concern (LC).

EOO 51,790 km^2^, AOO 44 km^2^. This species is not rare in Orenburg Region and has no tendency for declining.

##### Distribution

**Figure 14.**
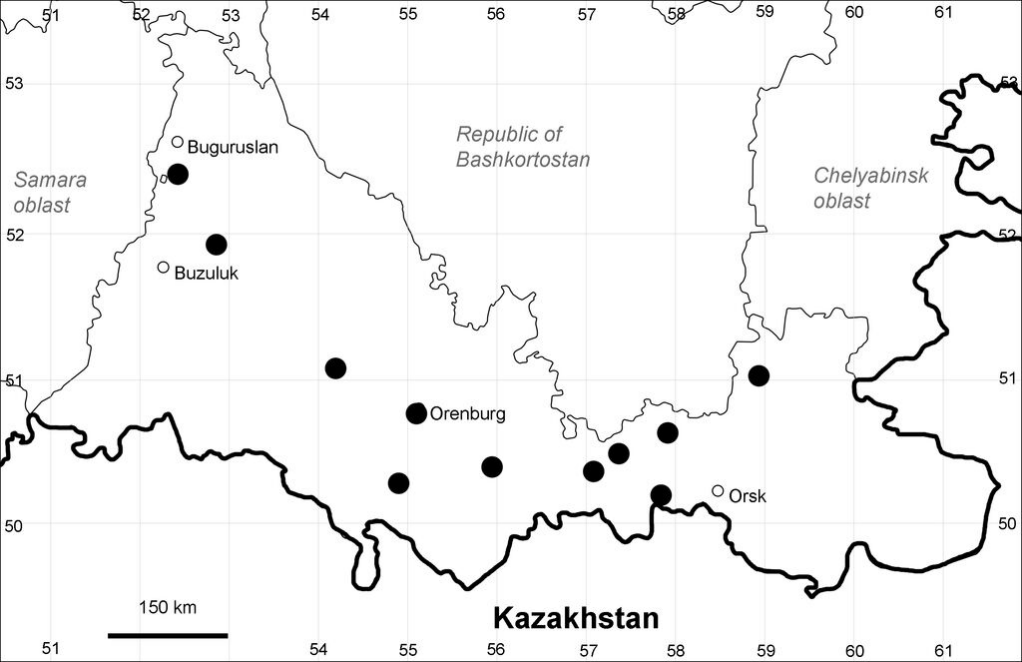
Distribution map of *Atriplexprostrata*.

Orenburg Region: Adamovka, Belyaevka, Buguruslan, Dombarovka, Gai, Grachevka, Kvarkeno, Kuvandyk, Sol'-Iletsk Districts, Orenburg City (Fig. 14).

Previous reports: Iljin (1930), Ryabinina (1998), Knjazev (2009).

The species occurs on saline substrates, along watercourses, also in human-disturbed sites. It is rather frequent in the Region.

The species is distributed as native in temperate Eurasia. It is found as alien in northern Africa, North and South America, southern Australia.

#### Atriplex sagittata

Borkh.

E53D7FF1-38FB-5596-BBB3-47DCD7869446

urn:lsid:ipni.org:names:164128-1

***Atriplexsagittata*** Borkh., Rhein. Mag. Erweit. Naturk. 1: 477 (1793).
= *Atriplexnitens* Schkuhr, Bot. Handb. 3: 541 (1802).

##### Native status

Native.

##### Conservation status

Least Concern (LC).

EOO 110,000 km^2^, AOO 76 km^2^. This species is very common in Orenburg Region and we assume its occurrence in all districts. It has no tendency for declining.

##### Distribution

**Figure 15.**
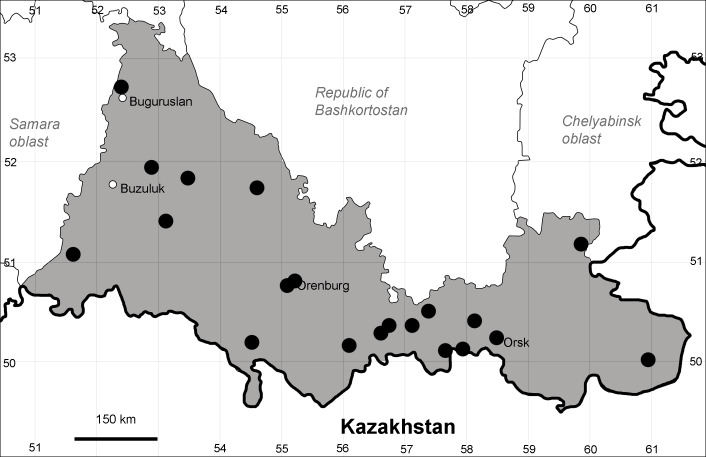
Distribution map of *Atriplexsagittata*, verified (points) and projected (shade) occurrence.

Orenburg Region (whole territory): Akbulak, Belyaevka, Buguruslan, Gai, Grachevka, Krasnaya Gvardia, Kuvandyk, Kvarkeno, Pervomaiskii, Severnoe, Sol'-Iletsk, Sorochinsk, Svetlyi Districts, Orsk Town, Orenburg City (Fig. 15).

Previous reports: Iljin (1930), Ryabinina (1998), Knjazev (2009).

The species occurs in steppes, meadows, along watercourses, also in human-disturbed sites. It is common in the Region.

The species is distributed as native in forest-steppes, steppes and semi-deserts of Eurasia and is widespread in the forest zone of Eurasia as an alien ruderal plant.

#### Atriplex sphaeromorpha

Iljin

BAE7A152-7B91-5602-8337-5D1CE61170C5

urn:lsid:ipni.org:names:77186039-1

***Atriplexsphaeromorpha*** Iljin, Izv. Glavnogo Bot. Sada 26(4): 414 (1927).

##### Native status

Native.

##### Conservation status

Data Deficient (DD).

A threatened category has not been assigned due to the absence of recent collections.

##### Distribution

**Figure 16.**
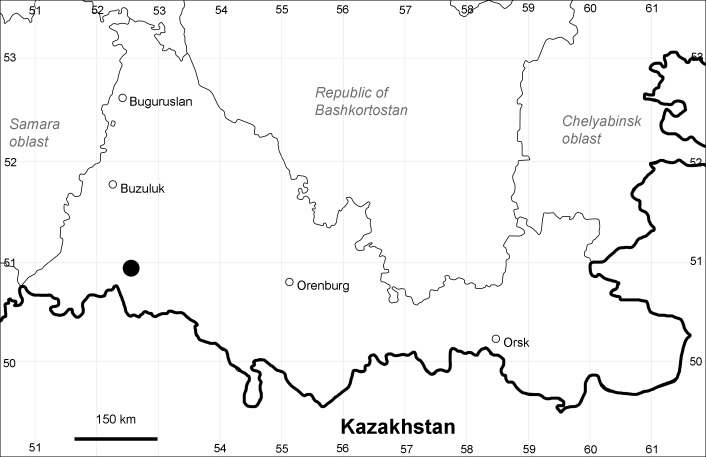
Distribution map of *Atriplexsphaeromorpha*.

Orenburg Region: Tashla District (Fig. 16).

Previous reports: Ryabinina (1998), Knjazev (2009).

The species occurs in steppes, sometimes in human-disturbed sites. It is very rare in the Region, known only from a single collection dated 1930s.

The species is distributed in the southern part of Eastern Europe (southern part of European Russia, southern Ukraine) and Central Asia (Kazakhstan, Uzbekistan: northern part of Karakalpakstan). It is known as a casual alien in Germany and the forest zone of European Russia.

#### Atriplex tatarica

L.

95548BF4-3F78-5D40-8E23-996EF87C4142

urn:lsid:ipni.org:names:164195-1

***Atriplextatarica*** L., Sp. Рl.: 1053 (1753).

##### Native status

Native.

##### Conservation status

Least Concern (LC).

EOO 110,000 km^2^, AOO 108 km^2^. This common species is an apophyte in Orenburg Region, without a tendency to decline.

##### Distribution

**Figure 17.**
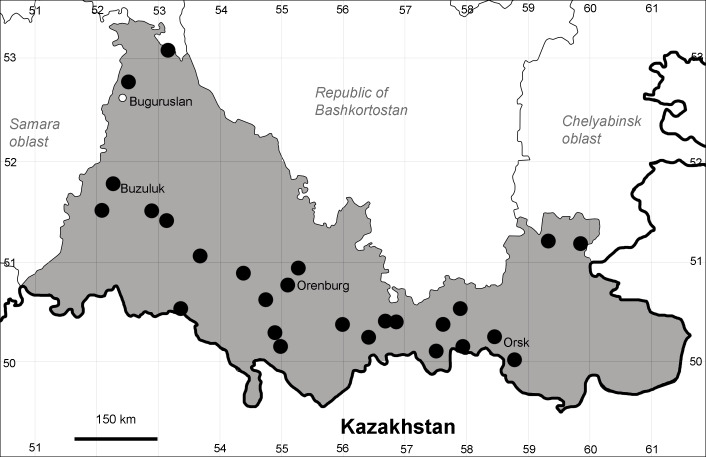
Distribution map of *Atriplextatarica*, verified (points) and projected (shade) occurrence.

Orenburg Region: Belyaevka, Buguruslan, Buzuluk, Dombarovka, Gai, Ilek, Kurmanaevka, Kuvandyk, Kvarkeno, Novosergievka, Perevolotskii, Sakmara, Severnoe, Sol'-Iletsk, Sorochinsk, Totskoe Districts, Orsk, Mednogorsk Towns, Orenburg City (Fig. 17).

Previous reports: Iljin (1930), Ryabinina (1998), Knjazev (2009).

The species occurs on saline substrates, also in human-disturbed sites. It is common in the Region.

The species is distributed as native in the Eastern Mediterranean and Eastern Europe, Caucasus, the Irano-Turanian Region. It is widely found as alien in Europe, North and South America.

#### Chenopodium acerifolium

Andrz.

0A1E4E52-77DF-5C8F-AC6C-3B03C1057AE0

urn:lsid:ipni.org:names:164748-1

***Chenopodiumacerifolium*** Andrz., Universitetskiye Izvestiya (Kiev) 7: 132 (1862).
= Chenopodiumalbumvar.hastatum C.Klinggr., Veg. Verh. Preuss. 2 Nachtr.: 130 (1866) ≡ Chenopodiumalbumvar.klinggraeffii Abrom., Fl. Ost- u. Westpreussen 2(2): 712 (1917), nom. illeg. ≡ *Chenopodiumklinggraeffii* Aellen, Fedd. Repert. 26: 159 (1929).

##### Native status

Native.

##### Conservation status

Data Deficient (DD).

A threatened category has not been assigned due to the absence of recent collections.

##### Distribution

**Figure 18.**
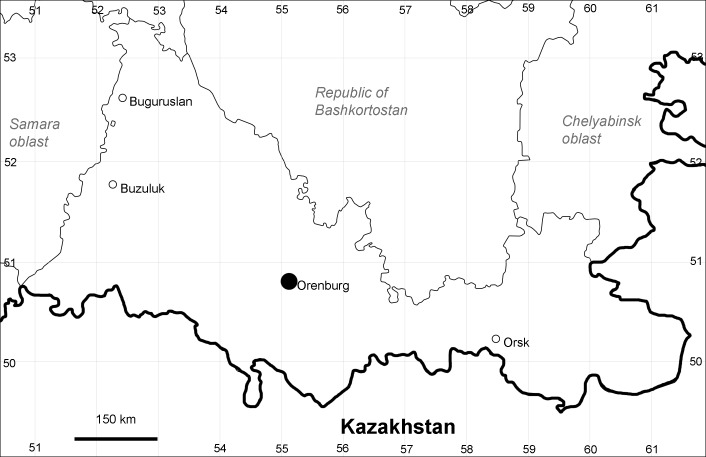
Distribution map of *Chenopodiumacerifolium*.

Orenburg Region: Orenburg City (Fig. 18).

Previous reports: Ryabinina (1998), Knjazev (2009).

The species occurs along riversides. It is known from the only old, undated specimen, first reported by Uotila and Lomonosova (2016).

The species is distributed in Eastern Europe and Siberia.

#### Chenopodium album

L.

7537B6A7-2B54-5BDB-AFB1-55DC4989CE03

urn:lsid:ipni.org:names:55993-2

***Chenopodiumalbum*** L., Sp. Pl. 1: 219 (1753).
The circumscription of this critical taxon requires further studies.

##### Native status

Native.

##### Conservation status

Least Concern (LC).

EOO 107,000 km^2^, AOO 92 km^2^. This common species is an apophyte in Orenburg Region, without a tendency to decline.

##### Distribution

**Figure 19.**
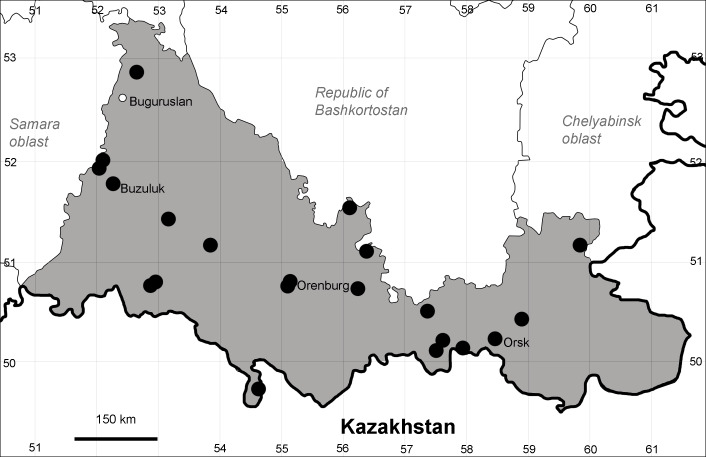
Distribution map of *Chenopodiumalbum*, confirmed (points) and projected (shade) occurrence.

Orenburg Region (whole territory): Buguruslan, Buzuluk, Gai, Kvarkeno, Kuvandyk, Novoorsk, Novosergievka, Saraktash, Sol'-Iletsk, Sorochinsk, Tashla, Tyulgan Districts, Orsk Town, Orenburg City (Fig. 19).

Previous records: Iljin (1930), Ryabinina (1998), Knjazev (2009).

The species occurs on disturbed lands. It is very common in the Region.

The species is widely distributed in the temperate zone of Eurasia and is found as alien in other continents.

#### Chenopodium betaceum

Andrz.

B6331B23-66DD-538C-9F3A-BDDE8E02D27B

urn:lsid:ipni.org:names:164812-1

***Chenopodiumbetaceum*** Andrz., Universitetskiye Izvestiya (Kiev) 7: 132 (1862).
= *Chenopodiumstriatiforme* Murr, Deutsche Bot. Monatsschr. 19: 51 (1901).
– *Chenopodiumstrictum* auct.: Ryabinina (1998), Mosyakin (1996).

##### Native status

Native.

##### Conservation status

Data Deficient (DD).

EOO 2,000 km^2^, AOO 12 km^2^. This species seems to be commonly overlooked by collectors. We agree with previous estimations (Ryabinina 1998, Knjazev 2009) that its actual occurrence should be much broader. For this reason, a threatened category has not been assigned.

##### Distribution

**Figure 20.**
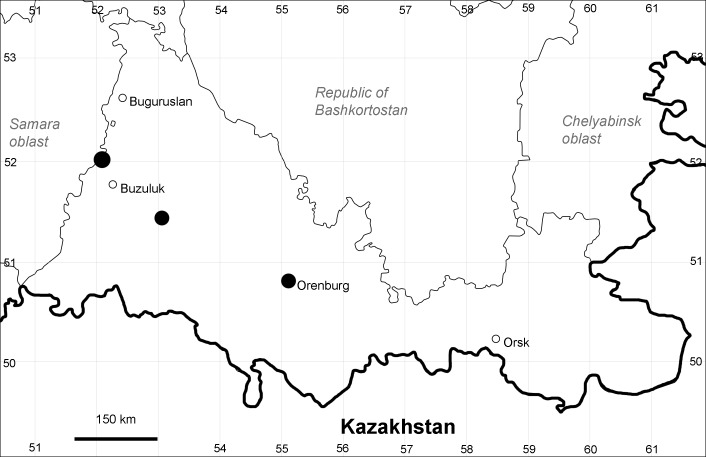
Distribution map of *Chenopodiumbetaceum* (confirmed occurrence).

Orenburg Region: Buzuluk, Sorochinsk Districts, Orenburg City (Fig. 20).

Previous records: Ryabinina (1998), Knjazev (2009), as *C.strictum* and *C.striatiforme*.

The species occurs on sands, along riversides and waterbodies, also in human-disturbed sites. It seems to occur sporadically in the region, mostly overlooked by collectors. Knjazev (2009) reported its occurrence in all districts of Orenburg Region.

The species is distributed in temperate Eurasia (eastwards to West Siberia).

#### Chenopodium opulifolium

Schrad. ex W.D.J.Koch & Ziz

1151635A-CFA5-5C2F-8721-562F7782D83A

urn:lsid:ipni.org:names:60447126-2

>***Сhenopodiumopulifolium*** Schrad. ex W.D.J.Koch & Ziz, Cat. Pl. Palat.: 6 (1814).
The earliest valid publication of a name for this taxon, albeit at the rank of variety (C.rubrumvar.opulifolium Schweigg.), was effected by A.F. Schweigger, based on the material received from the Botanical Garden in Paris (Schweigger 1812, p. 24).

##### Native status

Native.

##### Conservation status

Data Deficient (DD).

EOO and AOO not calculated. This species is an apophyte and, therefore, its actual occurrence may be wider in the territory. For this reason, a threatened category has not been assigned.

##### Distribution

**Figure 21.**
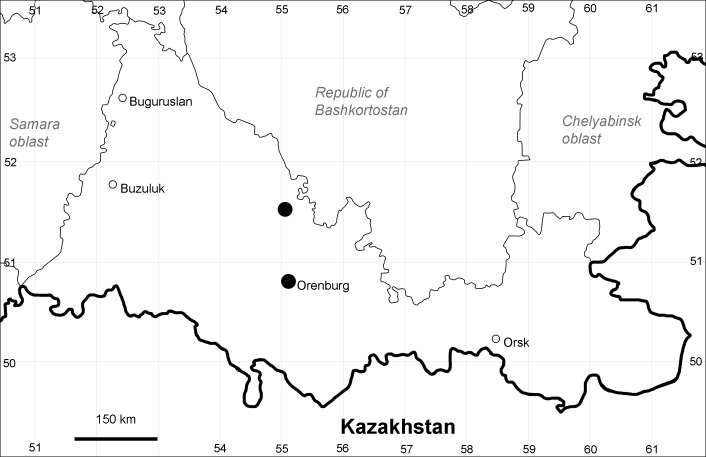
Distribution map of *Chenopodiumopulifolium*.

Orenburg Region: Oktiabrskoe District, Orenburg City (Fig. 21).

Previous reports: Ryabinina (1998), Knjazev (2009).

The species occurs on sands and also in human-disturbed sites. It is rare in the Region.

The species is distributed in Europe and Northern Africa.

#### Chenopodium virgatum

Thunb.

F66909A2-2112-5F84-B7D3-1B7BA19A7549

urn:lsid:ipni.org:names:165300-1

***Chenopodiumvirgatum*** Thunb., Nova Acta Reg. Soc. Sci. Upsal. 7: 143 (1815).
In the taxonomic interpretation of this species name, we follow Uotila (2001).

##### Native status

Casual alien.

##### Conservation status

Not applicable.

##### Distribution

**Figure 22.**
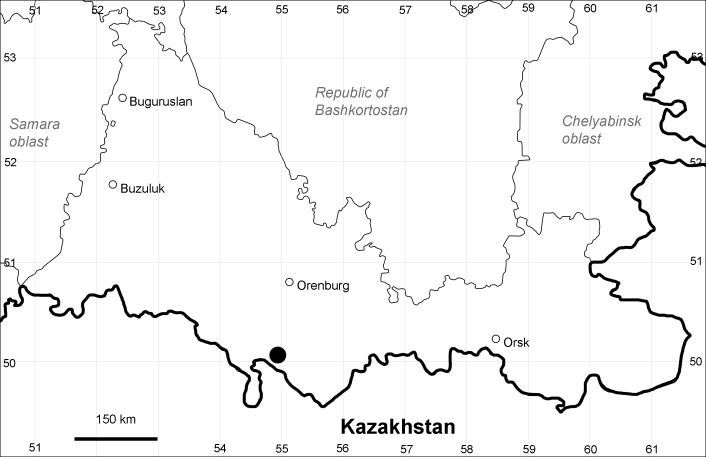
Distribution map of *Chenopodiumvirgatum*.

Orenburg Region: Sol'-Iletsk District (Fig. 22).

The species occurs on sands, in human-disturbed sites. It is first reported from the only locality here: 1 km E of Trudovoe, 28 Sep 2020, *A. Sukhorukov, N. Stepanova & O. Kalmykova 494* (MW).

The species is distributed in temperate Asia (Russia, China, Mongolia).

#### Chenopodiastrum hybridum

(L.) S.Fuentes, Uotila & Borsch

224ECC05-872D-59C0-A1F4-CCD5CF7A4DE2

urn:lsid:ipni.org:names:77121009-1

***Chenopodiastrumhybridum*** (L.) S.Fuentes, Uotila & Borsch, Willdenowia 42(1): 14 (2012) ≡ *Chenopodiumhybridum* L., Sp. Pl. 1: 219 (1753).

##### Native status

Native.

##### Conservation status

Least Concern (LC).

EOO 72,700 km^2^, AOO 64 km^2^. This common species presumably occurs through the whole territory of Orenburg Region, without a tendency to decline.

##### Distribution

**Figure 23.**
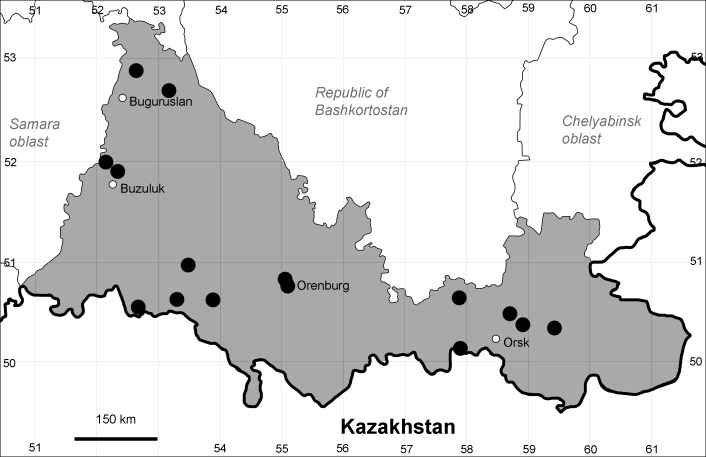
Distribution map of *Chenopodiastrumhybridum*, confirmed (points) and projected (shade) occurrence.

Orenburg Region (whole territory): Asekeevo, Buguruslan, Buzuluk, Gai, Ilek, Kuvandyk, Novoorsk, Novosergievka, Tashla Districts, Orenburg City (Fig. 23).

Previous reports: Iljin (1930), Ryabinina (1998), Knjazev (2009).

The species occurs in human-disturbed sites. It is common in the Region.

The species is distributed in temperate Eurasia.

#### Halimione pedunculata

(L.) Aellen

BDE28953-4A66-5E62-AD45-991627E2BADF

urn:lsid:ipni.org:names:165654-1

***Halimionepedunculata*** (L.) Aellen, Verh. Naturf. Ges. Basel 49: 123 (1938) ≡ *Atriplexpedunculata* L., Fl. Angl.: 25 (1754).

##### Native status

Native.

##### Conservation status

Least Concern (LC).

EOO 8,150 km^2^, AOO 20 km^2^. The species is locally common and further localities are expected.

##### Distribution

**Figure 24.**
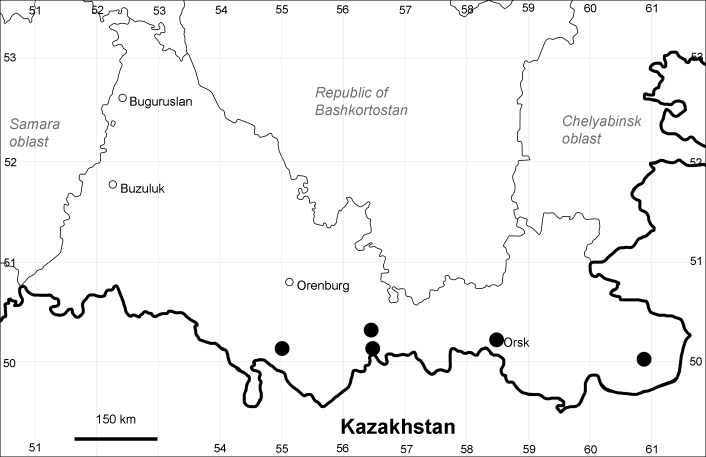
Distribution map of *Halimionepedunculata*.

Orenburg Region (southern districts): Akbulak, Belyaevka, Sol'-Iletsk, Svetlyi Districts, Orsk Town (Fig. 24). Northern limit of the species distribution.

Previous reports: Iljin (1930), Ryabinina (1998), Knjazev (2009).

The species occurs on saline substrates. It is found sporadically in the Region.

The species is distributed in Western, Central and Eastern (southern part) Europe, south-western Siberia and Central Asia (Kazakhstan, Uzbekistan).

#### Halimione verrucifera

(M.Bieb.) Aellen

8D0BEFB8-6E58-5C2D-B8E7-2B048143428D

urn:lsid:ipni.org:names:165656-1

***Halimioneverrucifera*** (M.Bieb.) Aellen, Verh. Naturf. Ges. Basel 49: 121 (1938) ≡ *Atriplexverrucifera* M.Bieb., Fl. Taur.-Cauc. 2: 441 (1808).

##### Native status

Native.

##### Conservation status

Least Concern (LC).

EOO 83,000 km^2^, AOO 76 km^2^. This species occurs almost in the whole territory. No decline is observed.

##### Distribution

**Figure 25.**
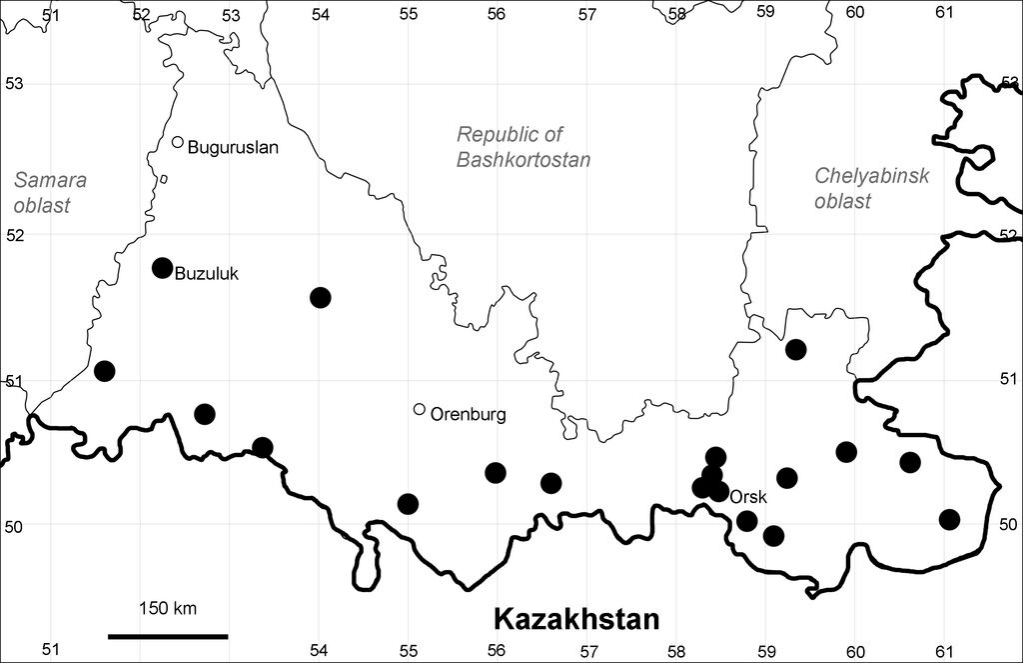
Distribution map of *Halimioneverrucifera*.

Orenburg Region (except northernmost districts): Adamovka, Belyaevka, Buzuluk, Dombarovka, Gai, Ilek, Kvarkeno, Novoorsk, Novosergievka, Pervomaiskii, Sol'-Iletsk, Svetlyi, Tashla Districts, Orsk Town (Fig. 25).

Previous reports: Iljin (1930), Ryabinina (1998), Knjazev (2009).

The species occurs on saline substrates. It is common in the Region.

The species is distributed in Eastern Europe (southern part), West Asia (Turkey), Caucasus, Central Asia (Kazakhstan, Uzbekistan, Kyrgyzstan), China (Xinjiang).

#### Lipandra polysperma

(L.) S.Fuentes, Uotila & Borsch

16FCC24B-20CC-5CE4-AF6B-FD39C089E6B3

urn:lsid:ipni.org:names:77121012-1

***Lipandrapolysperma*** (L.) S.Fuentes, Uotila & Borsch, Willdenowia 42(1): 14 (2012) ≡ *Chenopodiumpolyspermum* L., Sp. Pl. 1: 220 (1753).

##### Native status

Native.

##### Conservation status

Least Concern (LC).

EOO 56,300 km^2^, AOO 56 km^2^. The species is not rare in Orenburg Region. No decline is observed.

##### Distribution

**Figure 26.**
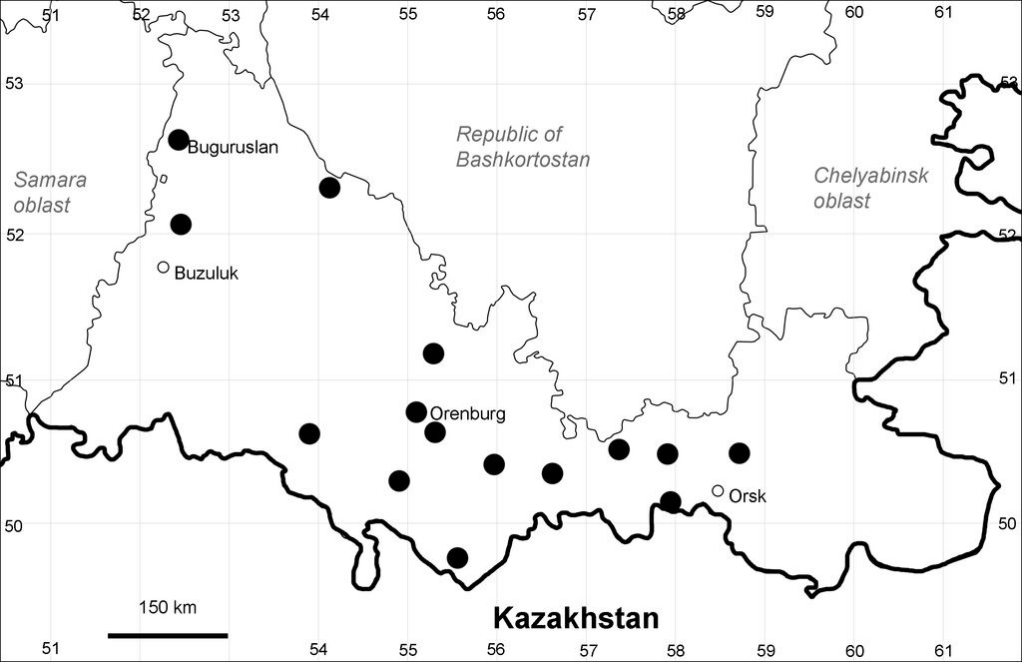
Distribution map of *Lipandrapolysperma*.

Orenburg Region: Belyaevka, Buguruslan, Buzuluk, Gai, Ilek, Kuvandyk, Ponomaryovka, Sol'-Iletsk Districts, Orenburg City (Fig. 26).

Previous reports: Ryabinina (1998), Knjazev (2009).

The species occurs on saline substrates, along watercourses and waterbodies. It was found sporadically in the Region.

The species is distributed as native in Eurasia. It was found as alien and naturalised in North America, South Africa and Australia.

#### Oxybasis chenopodioides

(L.) S.Fuentes, Uotila & Borsch

E915781D-1D17-5ECB-92F2-E79706CC1B81

urn:lsid:ipni.org:names:77121017-1

***Oxybasischenopodioides*** (L.) S.Fuentes, Uotila & Borsch, Willdenowia 42(1): 15 (2012) ≡ *Chenopodiumchenopodioides* (L.) Aellen, Ostenia: 98 (1933) ≡ *Blitumchenopodioides* L., Mant. Pl. Altera: 170 (1771).

##### Native status

Native.

##### Conservation status

Least Concern (LC).

EOO 42,350 km^2^, AOO 32 km^2^. The species widely occurs in the territory. No decline is observed.

##### Distribution

**Figure 27.**
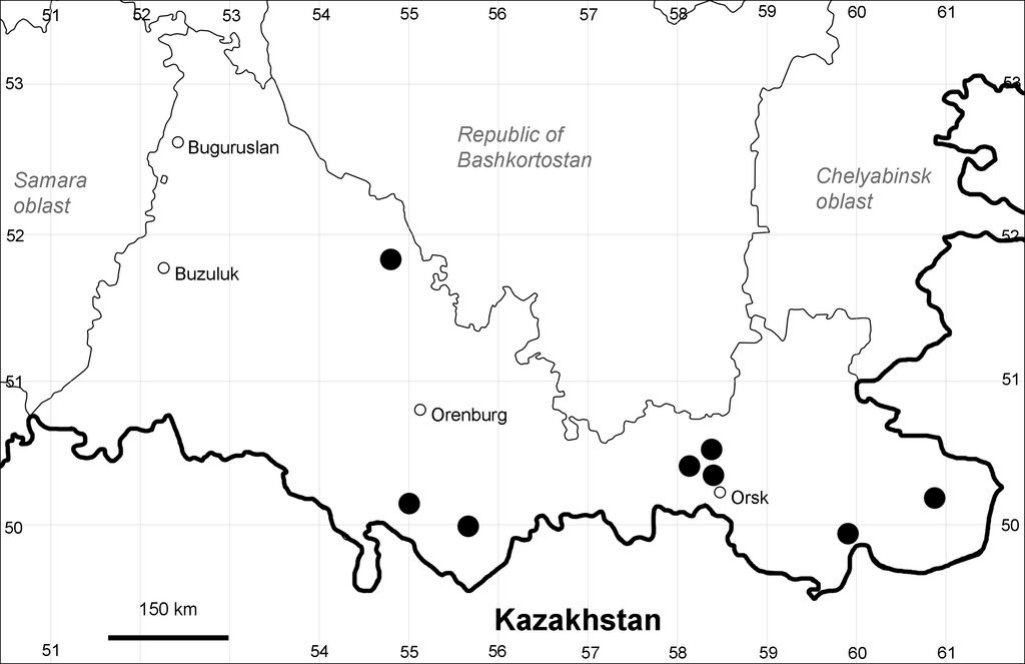
Distribution map of *Oxybasischenopodioides*.

Orenburg Region (central and eastern districts): Akbulak, Gai, Sharlyk, Sol'-Iletsk, Svetlyi, Yasnyi Districts (Fig. 27).

Previous reports: Ryabinina (1998), Knjazev (2009).

The species occurs on saline substrates. It is frequent in the Region.

The species is distributed as native in Europe, West Asia, eastern Mediterranean, Central Asia (Kazakhstan). It is known as alien in East and South Africa, North and South America.

#### Oxybasis glauca

(L.) S.Fuentes, Uotila & Borsch

56FBDD5F-0B82-5E97-8803-6C28CE244C8A

urn:lsid:ipni.org:names:77121014-1

***Oxybasisglauca*** (L.) S.Fuentes, Uotila & Borsch, Willdenowia 42(1): 15 (2012) ≡ *Chenopodiumglaucum* L., Sp. Pl. 1: 220 (1753).

##### Native status

Native.

##### Conservation status

Least Concern (LC).

EOO 41,600 km^2^, AOO 28 km^2^. The species is found in several places in the territory and a wider occurrence was indicated by Knjazev (2009). No decline is observed.

##### Distribution

**Figure 28.**
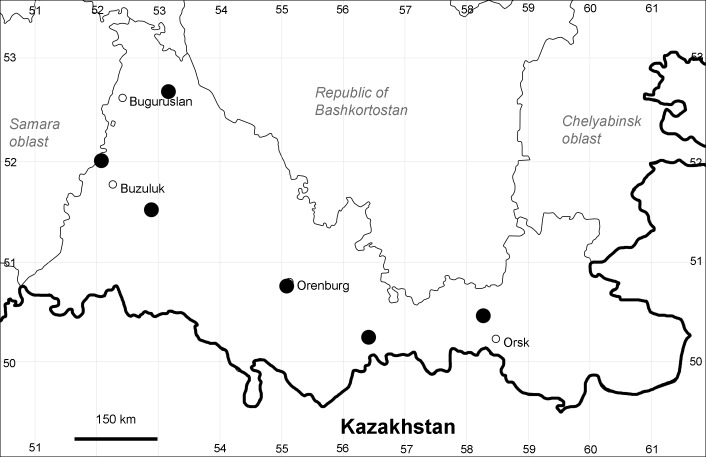
Distribution map of *Oxybasisglauca*.

Orenburg Region: Asekeevo, Belyaevka, Buzuluk, Gai, Ttoskoe Districts, Orenburg City (Fig. 28).

Previous reports: Iljin (1930), Ryabinina (1998), Knjazev (2009).

The species occurs along watercourses and waterbodies and also on human-disturbed and ruderal sites. It is found sporadically in the Region.

The species is distributed as native in Eurasia and North America. It is found as alien in Australia.

#### Oxybasis rubra

(L.) S.Fuentes, Uotila & Borsch

CE954385-CE2F-554E-AC6C-F5F20E83E9D2

urn:lsid:ipni.org:names:77121013-1

***Oxybasisrubra*** (L.) S.Fuentes, Uotila & Borsch, Willdenowia 42(1): 15 (2012) ≡ *Chenopodiumrubrum* L., Sp. Pl. 1: 218 (1753).

##### Native status

Native.

##### Conservation status

Least Concern (LC).

EOO 74,400 km^2^, AOO 36 km^2^. The species is found in several places in the territory and a wider occurrence was indicated by Knjazev (2009). No decline is observed.

##### Distribution

**Figure 29.**
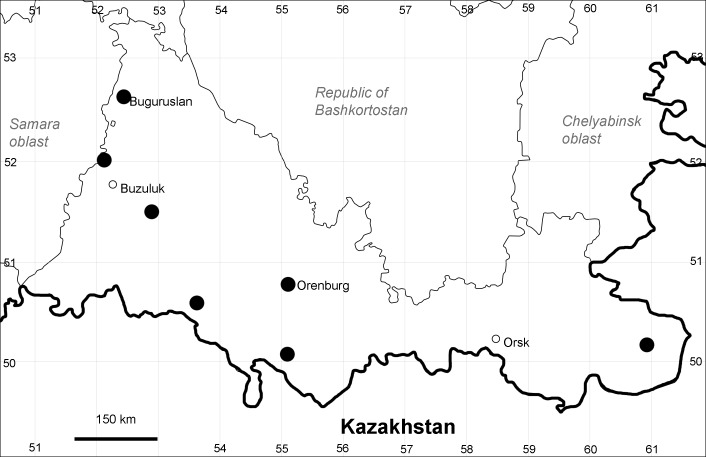
Distribution map of *Oxybasisrubra*.

Orenburg Region: Buguruslan, Buzuluk, Ilek, Sol'-Iletsk, Svetlyi, Totskoe Districts, Orenburg City (Fig. 29).

Previous reports: Iljin (1930), Ryabinina (1998), Knjazev (2009)

The species occurs along watercourses and waterbodies and also on human-disturbed and ruderal sites. It is found sporadically in the Region.

The species is distributed as native in temperate Eurasia and North Africa. It is found as alien in North America.

#### Oxybasis urbica

(L.) S.Fuentes, Uotila & Borsch

D7D256EF-8763-5D77-BF46-ADE74F19582A

urn:lsid:ipni.org:names:77121015-1

***Oxybasisurbica*** (L.) S.Fuentes, Uotila & Borsch, Willdenowia 42(1): 15 (2012) ≡ *Chenopodiumurbicum* L., Sp. Pl. 1: 218 (1753).

##### Native status

Native.

##### Conservation status

Least Concern (LC).

EOO 86,600 km^2^, AOO 60 km^2^. This common species presumably occurs through the whole territory of Orenburg Region, without a tendency to decline.

##### Distribution

**Figure 30.**
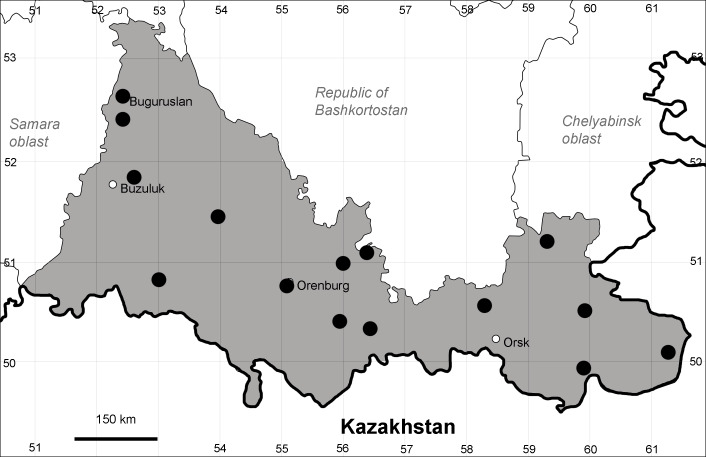
Distribution map of *Oxybasisurbica*, confirmed (points) and projected (shade) occurrence.

Orenburg Region (whole territory): Adamovka, Belyaevka, Buguruslan, Buzuluk, Gai, Kvarkeno, Novosergievka, Saraktash, Svetlyi, Tashla, Yasnyi Districts, Orenburg City (Fig. 30).

Previous reports: Iljin (1930), Ryabinina (1998), Knjazev (2009).

The species occurs in steppes, and also on human-disturbed and ruderal sites. It is common in the Region.

The species is distributed in steppes and semi-deserts of temperate Eurasia.

#### Axyris amaranthoides

L.

66475D43-68D1-5496-B478-BD33ABE8FCD8

urn:lsid:ipni.org:names:164269-1

***Axyrisamaranthoides*** L., Sp. Pl. 2: 979 (1753).

##### Native status

Established alien.

##### Conservation status

Not applicable.

##### Distribution

**Figure 31.**
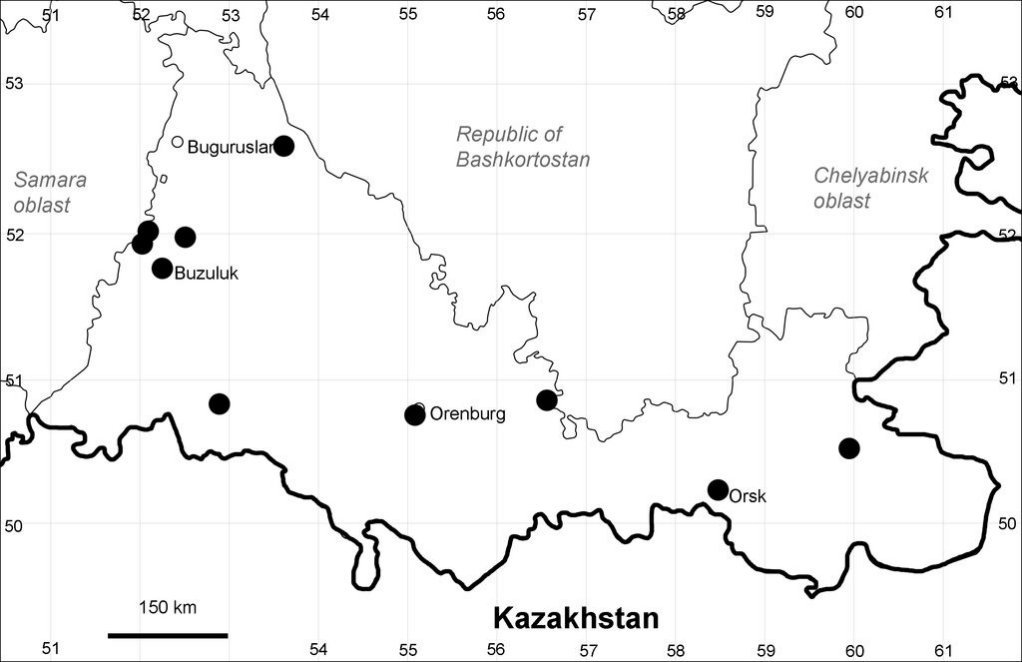
Distribution map of *Axyrisamaranthoides*.

Orenburg Region: Abdulino, Adamovka, Buzuluk, Saraktash, Tashla Districts, Orsk Town, Orenburg City (Fig. 31).

Previous reports: Iljin (1930), Ryabinina (1998), Knjazev (2009).

The species occurs mostly in human-disturbed sites. It is sporadically found in the Region.

The species is distributed as native in Asiatic Russia and Central Asia. It is known as alien in Western, Central and Eastern Europe, the Russian Far East and North America.

#### Ceratocarpus arenarius

L.

CC5CC036-EACE-50E3-8DA1-F896C6262165

urn:lsid:ipni.org:names:164656-1

***Ceratocarpusarenarius*** L., Sp. Pl. 1: 969 (1753).

##### Native status

Native.

##### Conservation status

Least Concern (LC).

EOO 101,250 km^2^, AOO 100 km^2^. This common species presumably occurs through the whole territory of Orenburg Region, without a tendency to decline.

##### Distribution

**Figure 32.**
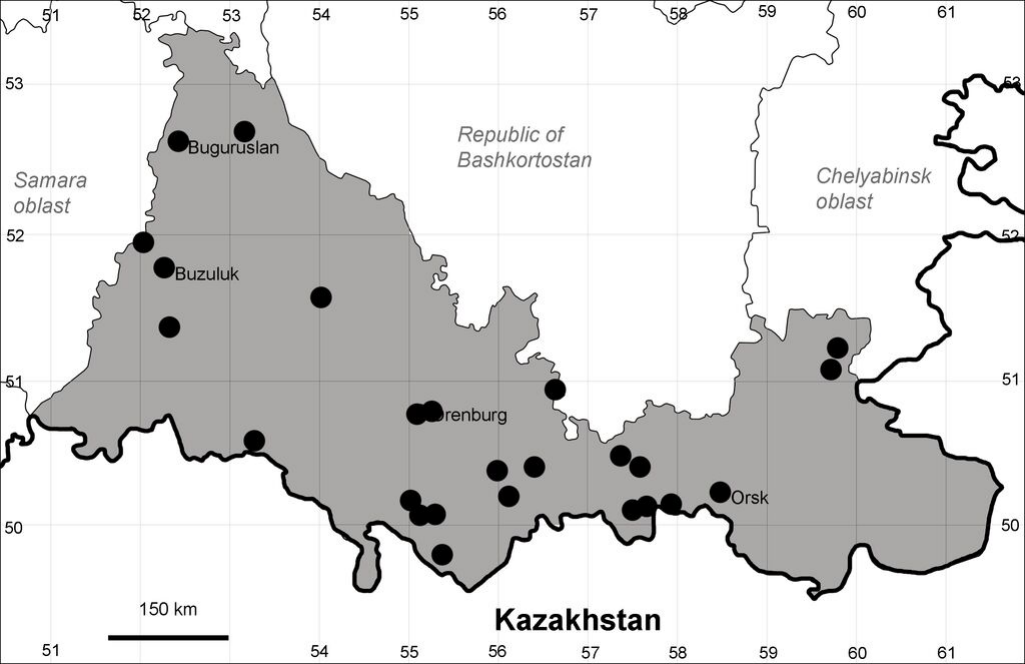
Distribution map of *Ceratocarpusarenarius*, confirmed (points) and projected (shade) occurrence.

Orenburg Region (whole territory): Akbulak, Asekeevo, Belyaevka, Buguruslan, Buzuluk, Gai, Ilek, Kuvandyk, Kvarkeno, Novosergievka, Saraktash, Sol'-Iletsk, Totskoe Districts, Orsk, Mednogorsk Towns, Orenburg City (Fig. 32).

Previous reports: Iljin (1930), Ryabinina (1998), Knjazev (2009).

The species occurs on sands, limestones, in degraded steppes and also in human-disturbed sites. It is common in the Region.

The species is distributed in steppes and deserts of Eurasia.

#### Krascheninnikovia ceratoides

(L.) Gueldenst.

A2B4C4DB-AF47-5F92-9EF3-7F55322EEC21

urn:lsid:ipni.org:names:166043-1

***Krascheninnikoviaceratoides*** (L.) Gueldenst., Nov. Comm. Ac. Sci. Petrop. 16: 555 (1772) ≡ *Axyrisceratoides* L., Sp. Pl. 2: 979 (1753) ≡ *Eurotiaceratoides* (L.) C.A.Mey. in Ledeb., Fl. Altaic. 4: 239 (1833).
= *Ceratospermumpapposum* Pers., Syn. Pl. 2(2): 552 (1807), nom. illeg. ≡ *Ceratoidespapposa* Botsch. & Ikonn., Nov. Sist. Vyssh. Rast. 6: 267 (1970).
= *Eurotialenensis* Kuminova, Sist. Zametki Mater. Gerb. Krylova Tomsk. Gosud. Univ. 1-2: 3 (1939) ≡ *Krascheninnikovialenensis* (Kuminova) Tzvelev in Ukr. Bot. Zhurn. 50(1): 78 (1993).

##### Native status

Native.

##### Conservation status

Least Concern (LC).

EOO 92,150 km^2^, AOO 140 km^2^. This common species presumably occurs through the whole territory of Orenburg Region, without a tendency to decline. A broader occurrence was indicated by Knjazev (2009).

##### Distribution

**Figure 33.**
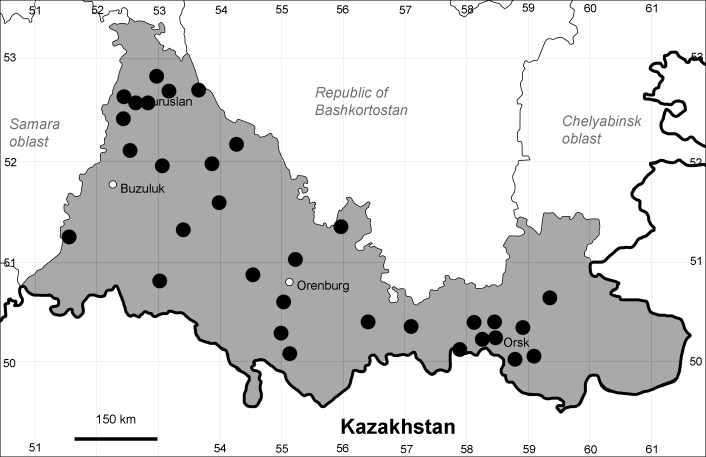
Distribution map of *Krascheninnikoviaceratoides*, confirmed (points) and projected (shade) occurrence.

Orenburg Region (whole territory): Abdulino, Adamovka, Asekeevo, Belyaevka, Buguruslan, Buzuluk, Dombarovskii, Gai, Grachevka, Krasnaya Gvardia, Kurmanaevka, Kuvandyk, Novoorsk, Novosergievka, Orsk, Perevolotskii, Ponomarevka, Sakmara, Sol'-Iletsk, Sorochinsk, Tashla, Tyulgan Districts, Orenburg City (Fig. 33).

Previous reports: Iljin (1930), Ryabinina (1998), Knjazev (2009).

The species occurs in steppes, on limestone. It is common in the Region.

The species is distributed in steppes and deserts of Eurasia, the Himalayas, Tibet, northern Africa, North America (subsp. lanata (Pursh) Heklau).

#### Agriophyllum pungens

(Vahl) Link

84E0BD3E-3E54-5532-9168-2D247E736BA4

urn:lsid:ipni.org:names:163348-1

***Agriophyllumpungens*** (Vahl) Link, Handbuch 2: 408 (Jun 1831); A. Dietr., Sp. Pl. 1: 124 (Oct 1831) ≡ *Corispermumpungens* Vahl, Enum. Pl. 1: 17 (1804) ≡ *Agriophyllumarenarium* M.Bieb. ex C.A.Mey., Verz. Pfl. Cauc. Casp. Meer: 163 (Nov 1831), nom. illeg. superfl.
– *Agriophyllumsquarrosum* auct.: Iljin (1930), Gusev (1996), Ryabinina (1998), Knjazev (2009).
Marschall von Bieberstein (1819) introduced the species name *Agriophyllumpungens* in discussions under *Coryspermumpungens*, indicating that it will be separated as a genus in the future. Czerepanov (1973) disregarded this statement as valid publication of the genus and species combination, apparently because of their provisional acceptance by the original author. He decided that the genus name was validly published by Meyer (1831) who merely accepted the genus with a reference to Bieberstein. However, Czerepanov failed to observe that Bieberstein described and discussed the species, but provided no description or diagnosis of the genus; besides, other authors accepted this genus name earlier than Meyer. Link (1831) was the first to accept the genus and provide its validating description, together with a combination for its only species. His work was published (according to contemporary announcements in *Staats- und Gelehrte Zeitung des Hamburgischen unpartheyischen Correspondenten* and *Kölnischer Correspondent*) by the beginning of June, whereas another book with the same taxonomic content (Dietrich 1831) was published in October (according to contemporary weekly bibliographies: *Bibliographie von Deutschland*). The work of Meyer (1831) was printed in November, according to internal evidence.

##### Native status

Native.

##### Conservation status

Least Concern (LC).

EOO 18,150 km^2^, AOO 16 km^2^. The species is known from a few scattered localities. Knjazev (2009) reported the occurrence in one more district in the south.

Although the species is rare in the territory, it occurs in open sands which are currently not threatened by human activity. As long as these landscapes are not in danger, we assess the conservation status of this species in Orenburg Region as Least Concern.

##### Distribution

**Figure 34.**
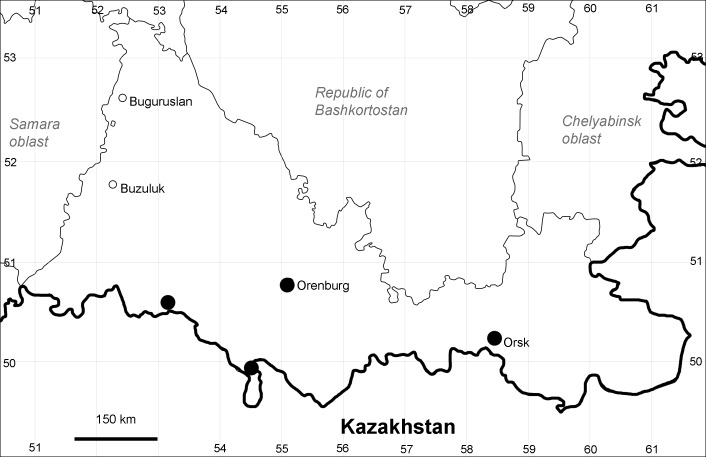
Distribution map of *Agriophyllumpungens*.

Orenburg Region: Ilek, Sol'-Iletsk Districts, Orsk Town, Orenburg City (Fig. 34). Northern limit of the species distribution.

Previous reports: Iljin (1930), Ryabinina (1998), Knjazev (2009).

The species occurs on sands. It is very rare in the southern part of the Region.

The species is distributed in Iran, the Caucasus, Central Asia.

#### Corispermum declinatum

Stephan ex Iljin

77F557CE-5723-5D58-9CE3-BFD45282834B

urn:lsid:ipni.org:names:165376-1

***Corispermumdeclinatum*** Stephan ex Iljin, Trydy Prikl. Bot. Gen. Selekts. 19(2): 69 (1928).

##### Native status

Naturalised alien.

##### Conservation status

Not applicable.

##### Distribution

**Figure 35.**
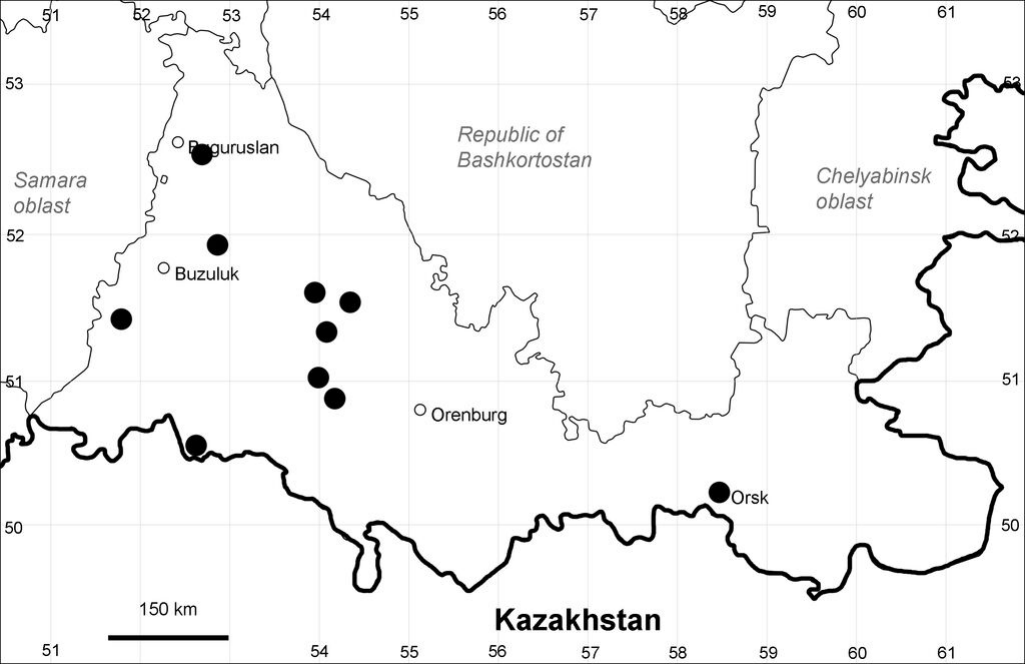
Distribution map of *Corispermumdeclinatum*.

Orenburg Region: Aleksandrovka, Asekeevo, Grachevka, Kurmanaevka, Novosergievka, Perevolotskii, Tashla Districts, Orsk Town (Fig. 35).

Previous reports: Iljin (1930), Ryabinina (1998), Knjazev (2009).

The species occurs on sands. It is rather frequent in the Region.

The species is distributed as native in Eastern Europe (south-eastern part), southern Siberia and Central Asia. It is known as alien in Western, Central and Eastern Europe and the Russian Far East.

#### Corispermum hyssopifolium

L.

1ECFAC41-3D42-5B3C-B33C-8B8F5DEACFDB

urn:lsid:ipni.org:names:327166-2

***Corispermumhyssopifolium*** L., Sp. Pl. 1: 4 (1753).

##### Native status

Native.

##### Conservation status

Least Concern (LC).

EOO 4,000 km^2^, AOO 12 km^2^. Despite its rarity and limited occurrence, the species is not threatened. Its habitats are not under the risk of destruction or degradation and further records may be expected when the territory is better explored.

##### Distribution

**Figure 36.**
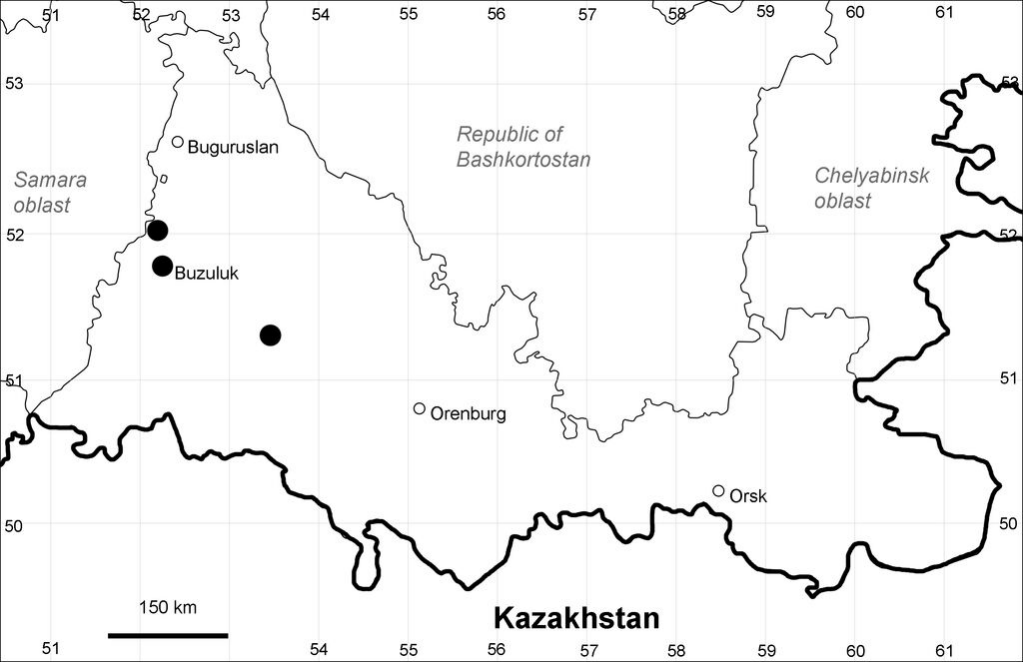
Distribution map of *Corispermumhyssopifolium*.

Orenburg Region (western districts): Buzuluk, Sorochinsk Districts (Fig. 36).

Previous reports: Iljin (1930), Ryabinina (1998), Knjazev (2009).

The species occurs on sands.

The species is distributed in Eastern Europe (south-eastern part), Western Siberia (southern part), Kazakhstan (north-western part).

#### Corispermum laxiflorum

Schrenk

C0D79F6A-6F7A-50CF-AA49-547843298AF3

urn:lsid:ipni.org:names:165410-1

***Corispermumlaxiflorum*** Schrenk, Bull. Cl. Phys.-Math. Acad. Imp. Sci. Saint-Pétersbourg 1: 361 (1843).

##### Native status

Native.

##### Conservation status

Data Deficient (DD).

A threatened category has not been assigned due to the absence of recent collections.

##### Distribution

**Figure 37.**
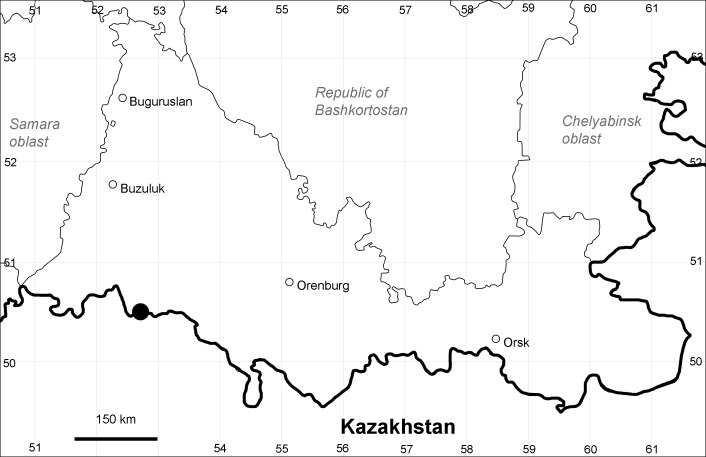
Distribution map of *Corispermumlaxiflorum*.

Orenburg Region: Tashla District (Fig. 37).

The species occurs on sands. The species is known from the only record in the Region, first reported here: Irtetskii [Irtek], 28.08.1928, *A.Borisova 1704* (LE).

The species is distributed in Eastern Europe (Russia: Saratov and Orenburg Regions) and Central Asia (Kazakhstan, Uzbekistan).

#### Corispermum marschallii

Steven

730068B5-A20F-51D4-A802-1550FECC6258

urn:lsid:ipni.org:names:165419-1

***Corispermummarschallii*** Steven, Mém. Soc. Imp. Naturalistes Moscou 5: 336 (1814).

##### Native status

Native.

##### Conservation status

Data Deficient (DD).

A threatened category has not been assigned due to the absence of recent collections. A broader occurrence is reported by Knjazev (2009).

##### Distribution

**Figure 38.**
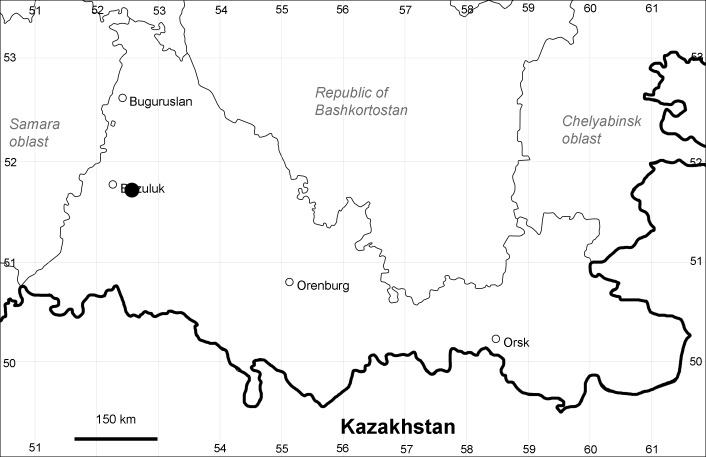
Distribution map of *Corispermummarschallii*.

Orenburg Region: Buzuluk District (Fig. 38).

Previous reports: Iljin (1930), Ryabinina (1998), Knjazev (2009).

The species occurs on sands and is distributed in Central and Eastern Europe.

#### Corispermum squarrosum

L.

1B3299D5-94CD-51F5-B521-3118762EC0B8

urn:lsid:ipni.org:names:165456-1

***Corispermumsquarrosum*** L., Sp. Pl. 1: 4 (1753).
= Corispermumsquarrosumsubsp.uralense Iljin, Izv. Glavn. Bot. Sada SSSR 28: 651 (1929) ≡ *Corispermumuralense* (Iljin) Aellen, Fedd. Repert. 69: 144 (1964).
– *Corispermumorientale* auct.: Iljin (1930), Mosyakin (1996), Ryabinina (1998), Knjazev (2009).

##### Native status

Native.

##### Conservation status

Data Deficient (DD).

A threatened category has not been assigned because the collection data seem to be highly incomplete. Knjazev (2009) reported a broader occurrence in the territory.

##### Distribution

**Figure 39.**
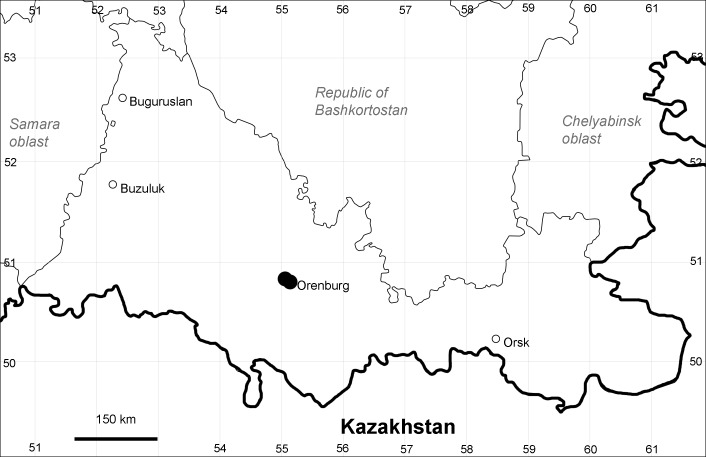
Distribution map of *Corispermumsquarrosum*.

Orenburg Region: Sakmara District, Orenburg City (Fig. 39).

Previous reports: Iljin (1930), Ryabinina (1998), Knjazev (2009), also as *C.orientale*.

The species occurs on sands. It may be rather common along the Ural River and its main tributories.

The species is distributed in Eastern Europe (south-eastern part: Orenburg Region), West Siberia (southern part) and Kazakhstan (north-western part).

#### Halocnemum strobilaceum

(Pall.) M.Bieb.

4A3B9B94-108B-57AE-8890-398FD9551F49

urn:lsid:ipni.org:names:165766-1

***Halocnemumstrobilaceum*** (Pall.) M.Bieb., Fl. Taur.-Cauc. 3: 3 (1819) ≡ *Salicorniastrobilacea* Pall, Reise 1: 412 (1771).

##### Native status

Native.

##### Conservation status

Least Concern (LC).

EOO 7,700 km^2^, AOO 36 km^2^. This species is restricted to the southern parts of the territory, but is not threatened.

##### Distribution

**Figure 40.**
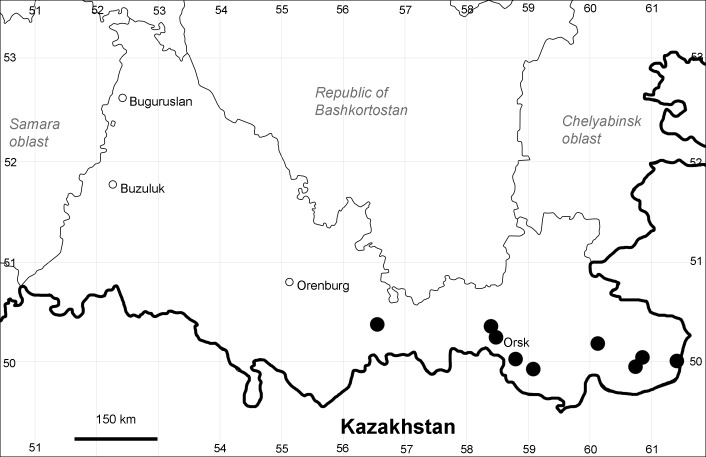
Distribution map of *Halocnemumstrobilaceum*.

Orenburg Region (south-eastern districts): Belyaevka, Dombarovka, Gai, Orsk, Svetlyi, Yasnyi Districts, Orsk Town (Fig. 40).

Previous reports: Ryabinina (1998), Knjazev (2009).

The species occurs on solonchaks. It is found in the south-eastern part of the Region.

The species is distributed in arid areas of Eurasia.

#### Kalidium caspicum

(L.) Ung.-Sternb.

7C71E7F6-65AA-5E65-B2C5-A25FC443B3E0

urn:lsid:ipni.org:names:165908-1

***Kalidiumcaspicum*** (L.) Ung.-Sternb., Atti Congr. Bot. Firenze 1874: 317 (1876) ≡ *Salicorniacaspica* L., Sp. Pl. 1: 4 (1753).

##### Native status

Native.

##### Conservation status

Data Deficient (DD).

A threatened category has not been assigned due to the absence of recent collections.

##### Distribution

**Figure 41.**
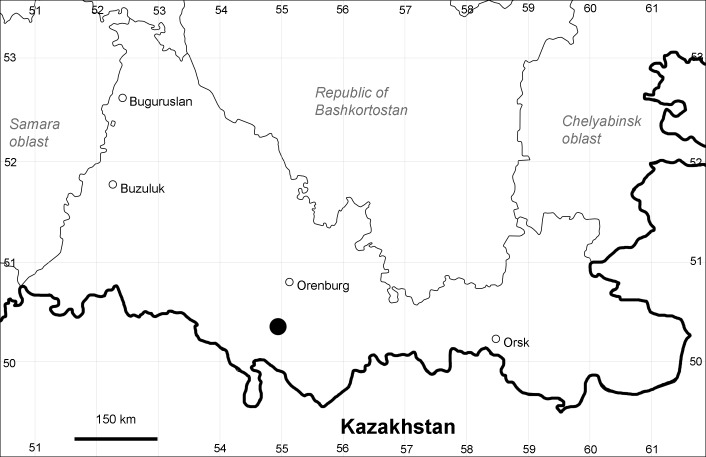
Distribution map of *Kalidiumcaspicum*.

Orenburg Region: Sol'-Iletsk District (Fig. 41).

Previous reports: Mavrodiev and Sukhorukov (2000), Knjazev (2009).

The species occurs on saline lands. It is known from a single old record in the Region, made in the 1910s.

The species is distributed in Eastern Europe (south-eastern part: the only locality in Orenburg Region), West Asia (Turkey), the Caucasus and Central Asia.

#### Kalidium foliatum

(Pall.) Moq.

9935B936-AB95-5DB3-A550-622632A60474

urn:lsid:ipni.org:names:165911-1

***Kalidiumfoliatum*** (Pall.) Moq. in DC., Prodr. 13(2): 147 (1849) ≡ *Salicorniafoliata* Pall., Reise 1: 422 (1771).

##### Native status

Native.

##### Conservation status

Least Concern (LC).

Currently under legal protection in Orenburg Region because of its rarity and restricted distribution, category 3 (Belov 2019).

EOO 800 km^2^, AOO 12 km^2^. This species has a restricted occurrence at the northern limit of its distribution. Nevertheless, its populations are confined to saline soils which are not threatened by human activities. For this reason, its further legal protection is considered unnecessary.

##### Distribution

**Figure 42.**
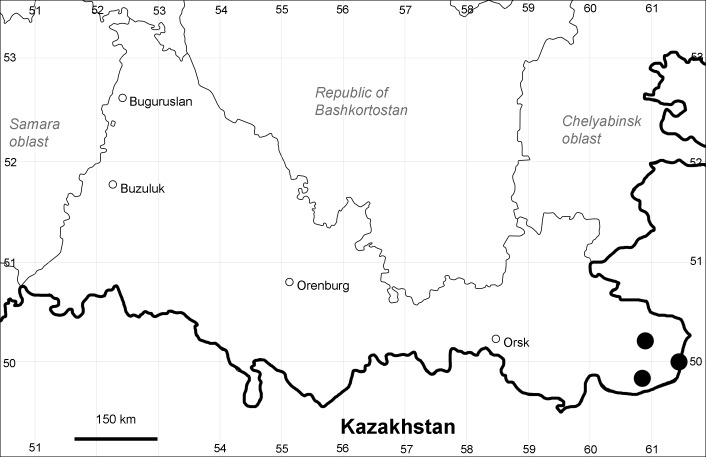
Distribution map of *Kalidiumfoliatum*.

Orenburg Region: Svetlyi District (Fig. 42).

Previous reports: Knjazev (2009).

The species occurs on saline substrates. It is restricted to the easternmost part of the Region.

The species is distributed in southern Russia, the Caucasus, Central Asia, Iran.

#### Salicornia perennans

Willd.

33AB78F9-4BB5-594A-985A-671F60F1BF6B

urn:lsid:ipni.org:names:166576-1

***Salicorniaperennans*** Willd., Sp. Pl. 1: 24 (1797).
= *Salicorniaprostrata* Pall., Ill. Pl.: 8, tab. 3 (1803).
– *Salicorniaeuropaea* auct.: Ryabinina (1998).
– *Salicorniaherbacea* auct.: Iljin (1930).

##### Native status

Native

##### Conservation status

Least Concern (LC).

EOO 63,000 km^2^, AOO 60 km^2^. The species is common in the southern districts and may be found sporadically occurring elsewhere.

##### Distribution

**Figure 43.**
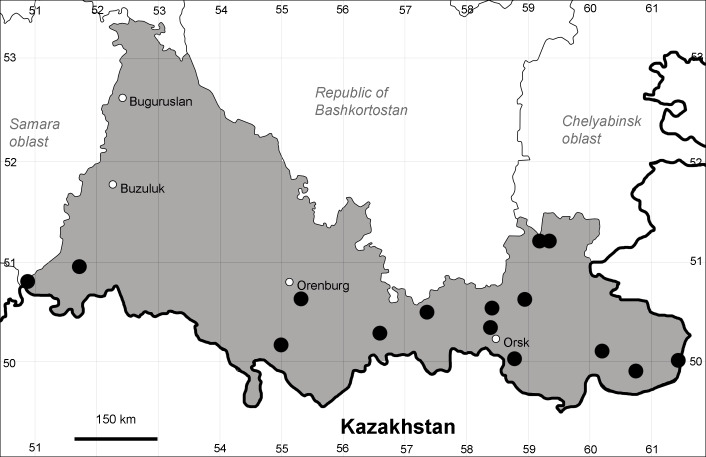
Distribution map of *Salicorniaperennans*, confirmed (points) and projected (shade) occurrence.

Orenburg Region (southern districts): Adamovka, Belyaevka, Dombarovka, Gai, Kuvandyk, Kvarkeno, Novoorsk, Pervomaiskii, Sol'-Iletsk, Svetlyi, Yasnyi Districts, Orenburg City (Fig. 43).

Previous reports: Iljin (1930), Ryabinina (1998), Knjazev (2009).

The species occurs on saline sites. It is common in the Region.

The species is distributed in arid regions of Eurasia.

#### Suaeda acuminata

(Ledeb.) Moq.

DA50697B-9493-5719-A559-337EE8D98D51

urn:lsid:ipni.org:names:167396-1

***Suaedaacuminata*** (Ledeb.) Moq., Ann. Sci. Nat. (Paris) 23: 306 (1831) ≡ *Schoberiaacuminata* Ledeb., Icon. Pl. Fl. Ross. 1: 11 (1829).
= *Suaedaconfusa* Iljin, Fl. Yugo-Vostoka Evr. Chasti SSSR 4: 196 (1930).

##### Native status

Native.

##### Conservation status

Least Concern (LC).

EOO 10,700 km^2^, AOO 24 km^2^. The species is rather common in the southern districts, without a tendency to decline.

##### Distribution

**Figure 44.**
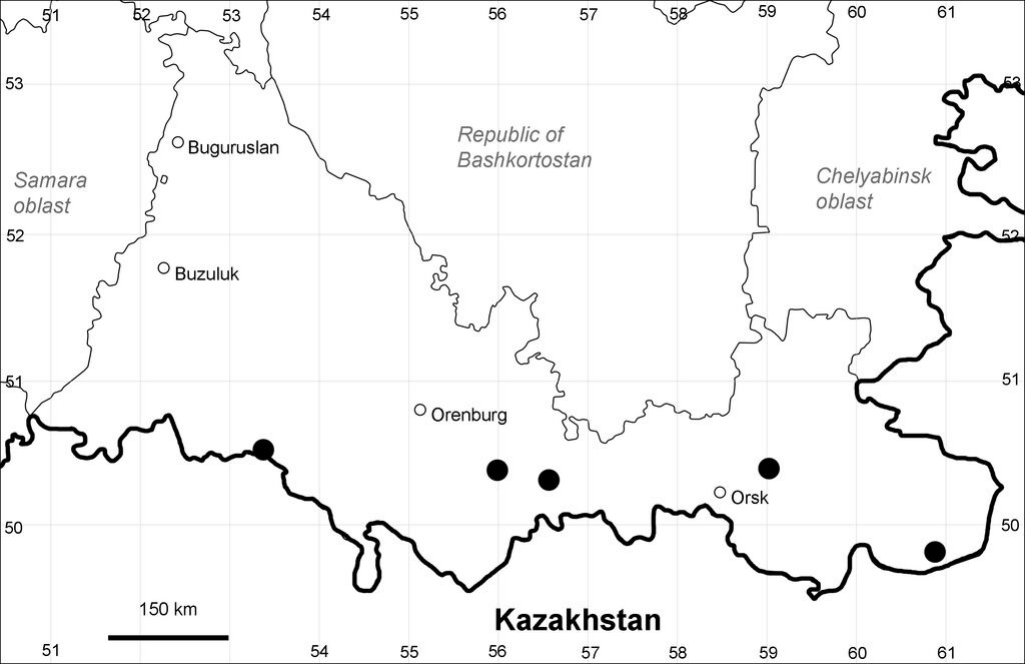
Distribution map of *Suaedaacuminata*.

Orenburg Region (southern districts): Belyaevka, Ilek, Novoorsk, Svetlyi Districts (Fig. 44). Northern limit of the species distribution.

Previous reports: Ryabinina (1998), Knjazev (2009).

The species occurs on saline substrates. It is frequent in the Region.

The species is distributed as native in the arid zone of Eurasia. It is known as a rare alien in the forest zone of Europe.

#### Suaeda corniculata

(C.A.Mey.) Bunge

15311E21-1413-5784-B4FA-2AB10252B466

urn:lsid:ipni.org:names:167442-1

***Suaedacorniculata*** (C.A.Mey.) Bunge, Trudy S.-Peterburgsk. Bot. Sada 6(2): 429 (1879) ≡ *Schoberiacorniculata* C.A.Mey. in Ledeb., Fl. Alt. 1: 399 (1829).

##### Native status

Native.

##### Conservation status

Least Concern (LC).

EOO 80,700 km^2^, AOO 72 km^2^. The species is common in the territory, without a tendency to decline.

##### Distribution

**Figure 45.**
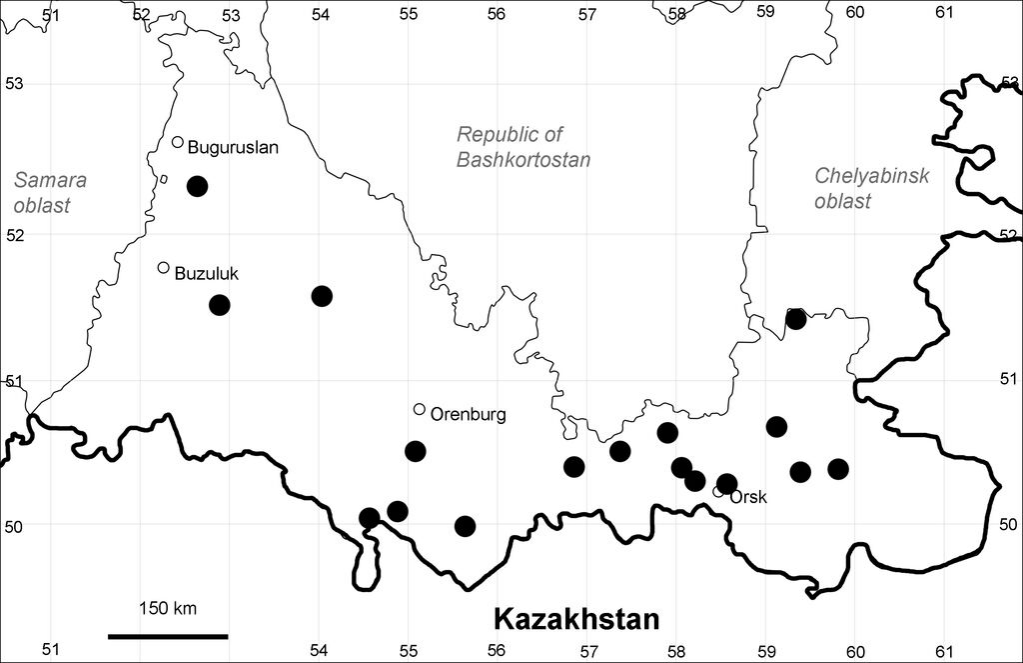
Distribution map of *Suaedacorniculata*.

Orenburg Region (whole territory): Adamovka, Akbulak, Asekeevo, Belyaevka, Gai, Kuvandyk, Kvarkeno, Novoorsk, Novosergievka, Orenburg, Sol'-Iletsk, Totskoe Districts, Orsk Town (Fig. 45).

Previous reports: Iljin (1930), Ryabinina (1998), Knjazev (2009).

The species occurs on saline substrates. It is frequent in the Region.

The species is distributed in Eastern Europe (Volga River), Siberia, Mongolia and northern China.

#### Suaeda kulundensis

Lomon. & Freitag

C72C025A-EA07-592E-BFCA-515F0982685A

urn:lsid:ipni.org:names:77091710-1

***Suaedakulundensis*** Lomon. & Freitag, Willdenowia 38(1): 99 (2008).

##### Native status

Native.

##### Conservation status

Least Concern (LC).

EOO 3,600 km^2^, AOO 16 km^2^. Further records are expected and no significant decline was observed or projected.

##### Distribution

**Figure 46.**
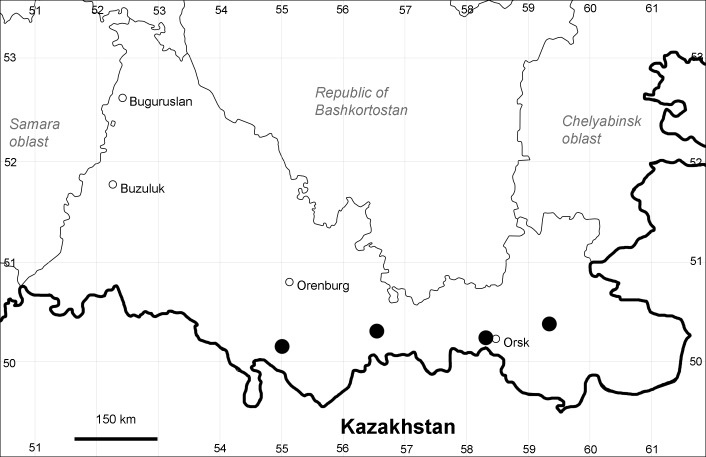
Distribution map of *Suaedakulundensis*.

Orenburg Region (south-eastern districts): Belyaevka, Novoorsk, Sol'-Iletsk Districts, Orsk Town (Fig. 46).

Previous reports: Lomonosova et al. (2008).

The species occurs on saline substrates.

The species is distributed in Eastern Europe (Volga River) and Siberia.

#### Suaeda linifolia

Pall.

DDA56D88-3235-588E-A37B-E0191815D78F

urn:lsid:ipni.org:names:167492-1

***Suaedalinifolia*** Pall., Ill. Pl.: 47 (1803).

##### Native status

Native.

##### Conservation status

Data Deficient (DD).

A threatened category has not been assigned due to the absence of recent collections.

##### Distribution

**Figure 47.**
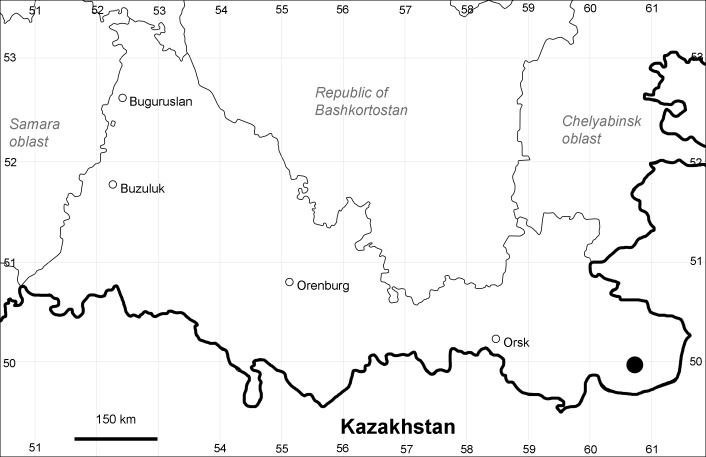
Distribution map of *Suaedalinifolia*.

Orenburg Region: Svetlyi District (Fig. 47).

Previous reports: Ryabinina (1998).

The species occurs on saline substrates. It is known from a single locality.

The species is distributed in Eastern Europe (south-eastern part), south-western Siberia, West and Central Asia, Iran, western China. It is known as alien in the western part of North America.

#### Suaeda physophora

Pall.

E389BE89-AD23-56EF-9C6F-D649A29E77DF

urn:lsid:ipni.org:names:167542-1

***Suaedaphysophora*** Pall., Ill. Pl.: 51 (1803).

##### Native status

Native.

##### Conservation status

Least Concern (LC).

EOO 28,800 km^2^, AOO 32 km^2^. The species was found in several localities, without a tendency to decline.

##### Distribution

**Figure 48.**
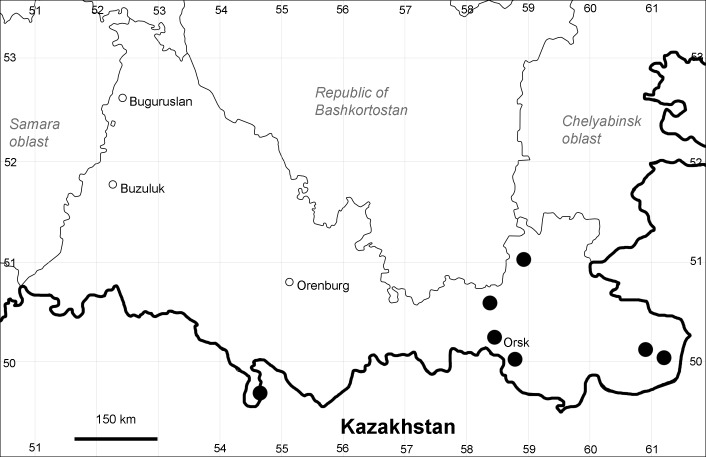
Distribution map of *Suaedaphysophora*.

Orenburg Region (southern and eastern districts): Dombarovka, Gai, Kvarkeno, Sol'-Iletsk, Svetlyi Districts, Orsk Town (Fig. 48).

Previous reports: Iljin (1930), Ryabinina (1998), Knjazev (2009).

The species occurs on saline substrates. It is rare in the southern and eastern parts of the Region.

The species is distributed in Eastern Europe (south-east European Russia), the Caucasus (eastern part), Iran (northern part), Kazakhstan, western China.

#### Suaeda prostrata

Pall.

D9087FF6-1DA5-5268-92B2-FDDD90EFF81D

urn:lsid:ipni.org:names:167547-1

***Suaedaprostrata*** Pall., Ill. Pl.: 55 (1803).
– *Suaedamaritima* auct.: Iljin (1930).

##### Native status

Native.

##### Conservation status

Least Concern (LC).

EOO 60,000 km^2^, AOO 40 km^2^. The species is common in the territory and its wider occurrence is expected (as reported by Knjazev (2009)). No evidence of declining.

##### Distribution

**Figure 49.**
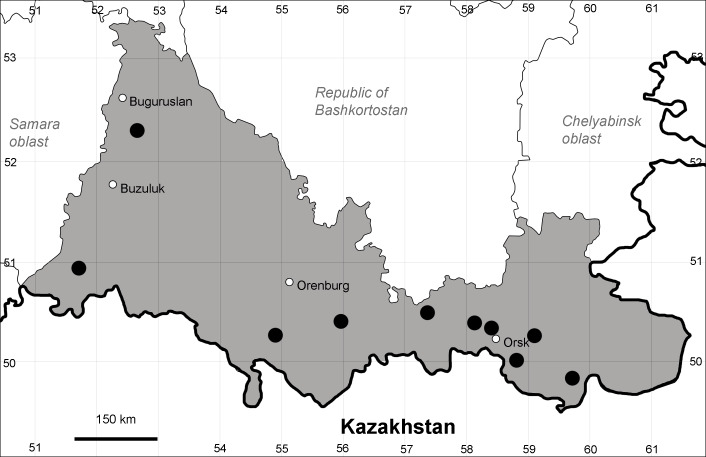
Distribution map of *Suaedaprostrata*, confirmed (points) and projected (shade) occurrence.

Orenburg Region (whole territory): Asekeevo, Belyaevka, Dombarovka, Gai, Kuvandyk, Novoorsk, Pervomaiskii, Sol'-Iletsk Districts (Fig. 49).

Previous reports: Ryabinina (1998), Knjazev (2009).

The species occurs on saline substrates. It is common in the Region.

The species is distributed in the steppe and forest-steppe zones of Eurasia, from Central Europe to western China.

#### Suaeda salsa

(L.) Pall.

2005AB61-EDE7-5FF1-8A64-3B11E2164C63

urn:lsid:ipni.org:names:167562-1

***Suaedasalsa*** (L.) Pall., Ill. Pl.: 46 (1803) ≡ *Chenopodiumsalsum* L., Sp. Pl. 1: 221 (1753).

##### Native status

Native.

##### Conservation status

Least Concern (LC).

EOO 14,100 km^2^, AOO 32 km^2^. The species is common in the south and no decline was observed or projected.

##### Distribution

**Figure 50.**
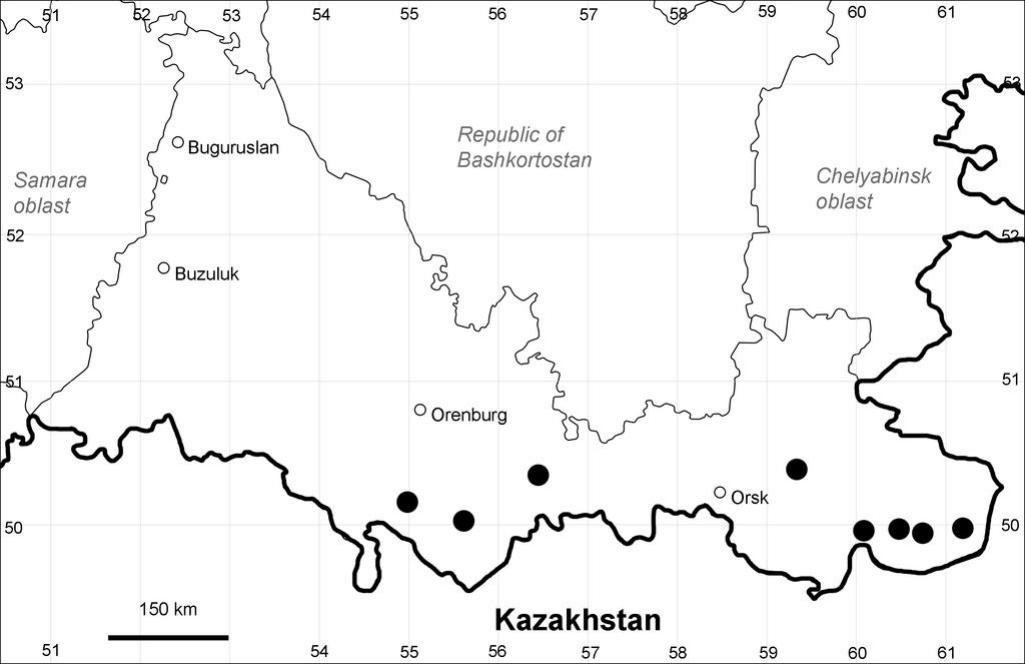
Distribution map of *Suaedasalsa*.

Orenburg Region (southern districts): Akbulak, Belyaevka, Novoorsk, Sol'-Iletsk, Svetlyi, Yasnyi Districts (Fig. 50).

Previous reports: Iljin (1930), Knjazev (2009).

The species occurs on saline substrates. It is common in the Region.

The species is distributed in Eastern Europe (south-eastern part), the Caucasus, Kazakhstan, southern Siberia.

#### Bassia hyssopifolia

(Pall.) Kuntze

C83E2787-4822-59FD-B0FA-E23EAD5755C4

urn:lsid:ipni.org:names:29802-2

***Bassiahyssopifolia*** (Pall.) Kuntze, Rev. Gen. Pl. 2: 547 (1891) ≡ *Salsolahyssopifolia* Pall., Reise 1: 491 (1771).

##### Native status

Native.

##### Conservation status

Least Concern (LC).

The species is known from a single locality, in which it was collected twice during a long period (1955, 2016). The population seems to be healthy and out of immediate danger; no further details were reported by Golovanov et al. (2018) who visited the place.

##### Distribution

**Figure 51.**
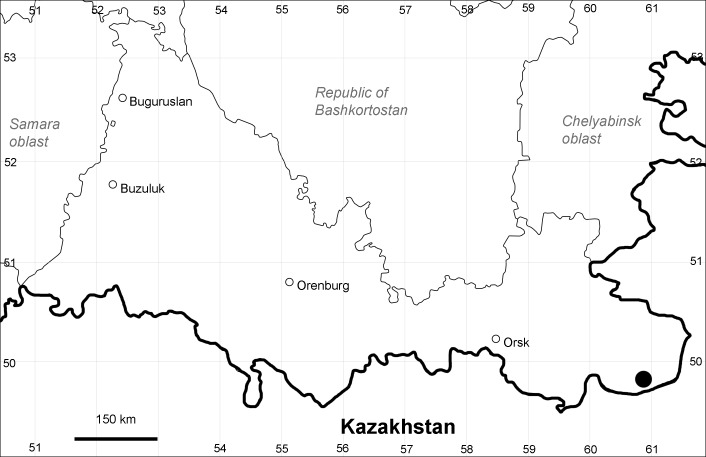
Distribution map of *Bassiahyssopifolia*.

Orenburg Region: Svetlyi District (Fig. 51).

Previous reports: Ryabinina (1998), Golovanov et al. (2018).

The species occurs on saline substrates. It is very rare in the eastern part of the Region.

The species is distributed in Eastern Europe (south-eastern part), West Asia (southwards to Iraq and Saudi Arabia), Central Asia. It is known as a rare alien in the forest zone of Europe, in Australia, North and South America.

#### Bassia laniflora

(S.G.Gmel.) A.J.Scott

73D1815E-2070-5B78-AA79-39C5F3EFEA49

urn:lsid:ipni.org:names:164386-1

***Bassialaniflora*** (S.G.Gmel.) A.J.Scott, Fedd. Repert. 89(2–3): 108 (1978) ≡ *Salsolalaniflora* S.G.Gmel., Reise 1: 160 (1774) ≡ *Kochialaniflora* (S.G.Gmel.) Borb., Balaton Fl.: 340 (1900).

##### Native status

Native.

##### Conservation status

Least Concern (LC).

EOO 24,500 km^2^, AOO 32 km^2^. The species was found in several localities covering the western part of the territory and no decline was observed or projected.

##### Distribution

**Figure 52.**
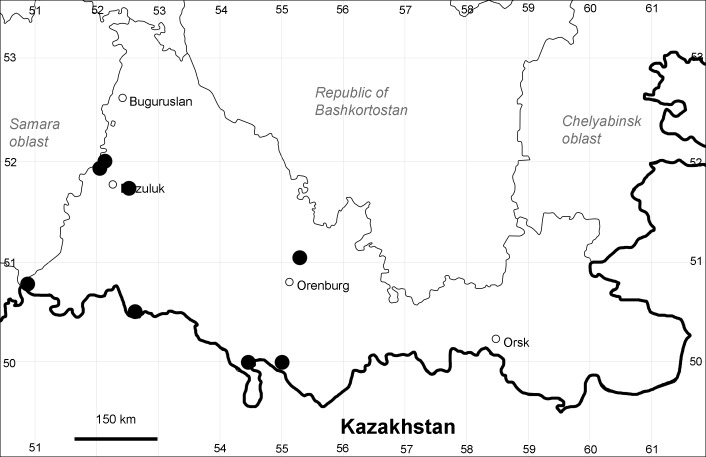
Distribution map of *Bassialaniflora*.

Orenburg Region (western districts): Buzuluk, Pervomaiskii, Sakmara, Sol'-Iletsk, Tashla Districts (Fig. 52).

Previous reports: Iljin (1930), Ryabinina (1998), Knjazev (2009).

The species occurs on sands. It is sporadically found in the Region.

The species is distributed in Central and Southern Europe, the Caucasus, Kazakhstan, southern Siberia.

#### Bassia prostrata

(L.) Beck

ECAB0B36-F0EB-5E7F-A008-D10E16E1C571

urn:lsid:ipni.org:names:60447623-2

***Bassiaprostrata*** (L.) Beck in Reichenbach, Icon. Fl. Germ. Helv. 24: 155 (1909) ≡ *Kochiaprostrata* (L.) Schrad., Neues J. Bot. 3(3–4): 85 (1809) ≡ *Salsolaprostrata* L., Sp. Pl. 1: 222 (1753).

##### Native status

Native.

##### Conservation status

Least Concern (LC).

EOO 120,800 km^2^, AOO 196 km^2^. The species occurs abundantly and is very common in the territory.

##### Distribution

**Figure 53.**
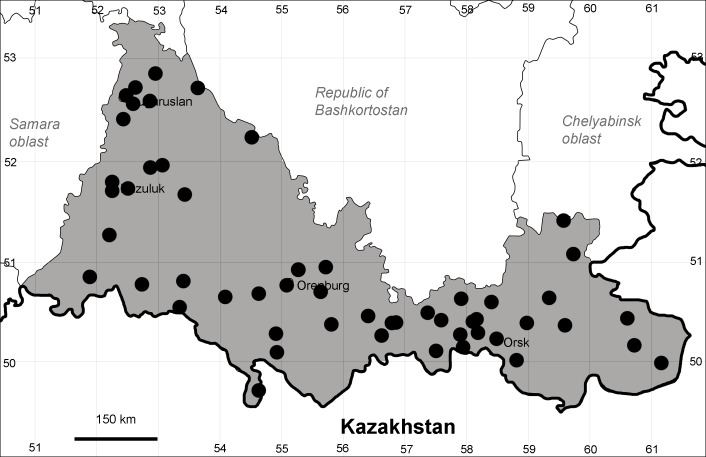
Distribution map of *Bassiaprostrata*, confirmed (points) and projected (shade) occurrence.

Orenburg Region (whole territory): Abdulino, Adamovka, Akbulak, Asekeevo, Belyaevka, Buguruslan, Buzuluk, Dombarovka, Gai, Grachevka, Ilek, Krasnaya Gvargia, Kvarkeno, Kuvandyk, Novoorsk, Novosergievka, Orenburg, Perevolotskii, Pervomaiskii, Ponomarevka, Sakmara, Sol'-Iletsk, Svetlyi, Tashla, Totskoe, Yasnyi Districts, Orsk Town (Fig. 53).

Previous reports: Iljin (1930), Ryabinina (1998), Knjazev (2009).

The species occurs in steppes, on limestone. It is common in the Region.

The species is distributed as native in Eurasia (from steppes to deserts) and locally in North Africa (Morocco). It is known as alien in North America.

#### Bassia scoparia

(L.) Beck

5F5283E1-BA37-5004-8FB1-897EEDBB6F7F

urn:lsid:ipni.org:names:77325913-1

***Bassiascoparia*** (L.) Beck in Reichenbach, Icon. Fl. Germ. Helv. 24: 155 (1909) ≡ *Kochiascoparia* (L.) Schrad., Neues J. Bot. 3((3-4): 85 (1809) ≡ *Chenopodiumscoparia* L., Sp. Pl. 1: 221 (1753).
= *Suaedasieversiana* Pall., Ill. Pl.: 45 (1803) ≡ *Kochiasieversiana* (Pall.) C.A.Mey. in Ledeb., Fl. Altaic. 1: 415 (1829).
= Kochiascopariavar.densiflora Moq. in DC., Prodr. 13(2): 131 (1849) ≡ *Kochiadensiflora* (Moq.) Aellen, Mitt. Basler Bot. Ges. 2(1): 13 (1954) ≡ Kochiascopariasubsp.densiflora (Moq.) Aellen in Hegi, Ill. Fl. Mitteleur., ed. 2, 3/2: 710 (1961).

##### Native status

Established alien.

##### Conservation status

Not applicable.

##### Distribution

**Figure 54.**
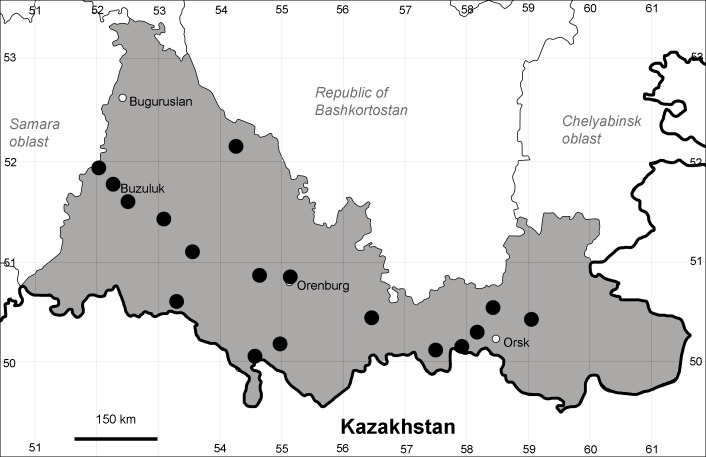
Distribution map of *Bassiascoparia*, confirmed (points) and projected (shade) occurrence.

Orenburg Region: Belyaevka, Buzuluk, Gai, Ilek, Kuvandyk, Novoorsk, Novosergievka, Perevolotsky, Ponomaryovka, Sol'-Iletsk, Sorochinsk, Totskoe Districts, Orenburg City (Fig. 54).

Previous reports: Ryabinina (1998), Knjazev (2009).

The species occurs along railroad beds, in populated places and their closest proximity.

The species is distributed as native in Central Asia (western China, Mongolia, southern Siberia). It is widely known as alien and naturalised in the temperate zone around the World. It spreads along roads and has arrived to the territory due to its formerly common cultivation as a technical plant (used for brooms in villages).

#### Camphorosma lessingii

Litv.

965A4CA8-C2BE-5C06-A538-8B7681407209

urn:lsid:ipni.org:names:164611-1

***Camphorosmalessingii*** Litv., Trudy Bot. Muz. Imp. Acad. Nauk 2: 96 (1905).

##### Native status

Native.

##### Conservation status

Least Concern (LC).

EOO 17,200 km^2^, AOO 44 km^2^. The species is common in the eastern part of the territory and no decline was observed or projected.

##### Distribution

**Figure 55.**
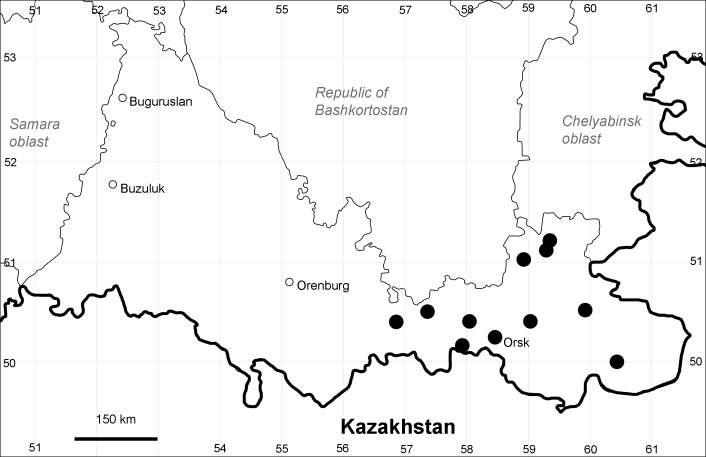
Distribution map of *Camphorosmalessingii*.

Orenburg Region (eastern districts): Adamovka, Belyaevka, Gai, Kuvandyk, Kvarkeno, Novoorsk, Yasnyi Districts, Orsk Town (Fig. 55). Northern limit of the species distribution.

Previous reports: Ryabinina (1998), Knjazev (2009).

The species occurs on saline substrates. It is frequent in the Region.

The species is distributed as native in Eastern Europe (south-eastern part) and Central Asia.

#### Camphorosma monspeliaca

L.

678E67C6-D57B-5988-862F-4B7EBAEBA06A

urn:lsid:ipni.org:names:164614-1

***Camphorosmamonspeliaca*** L., Sp. Pl. 1: 122 (1753).

##### Native status

Native.

##### Conservation status

Least Concern (LC).

EOO 47,500 km^2^, AOO 64 km^2^. The species is common in the southern parts of the territory and shows no sign of decline.

##### Distribution

**Figure 56.**
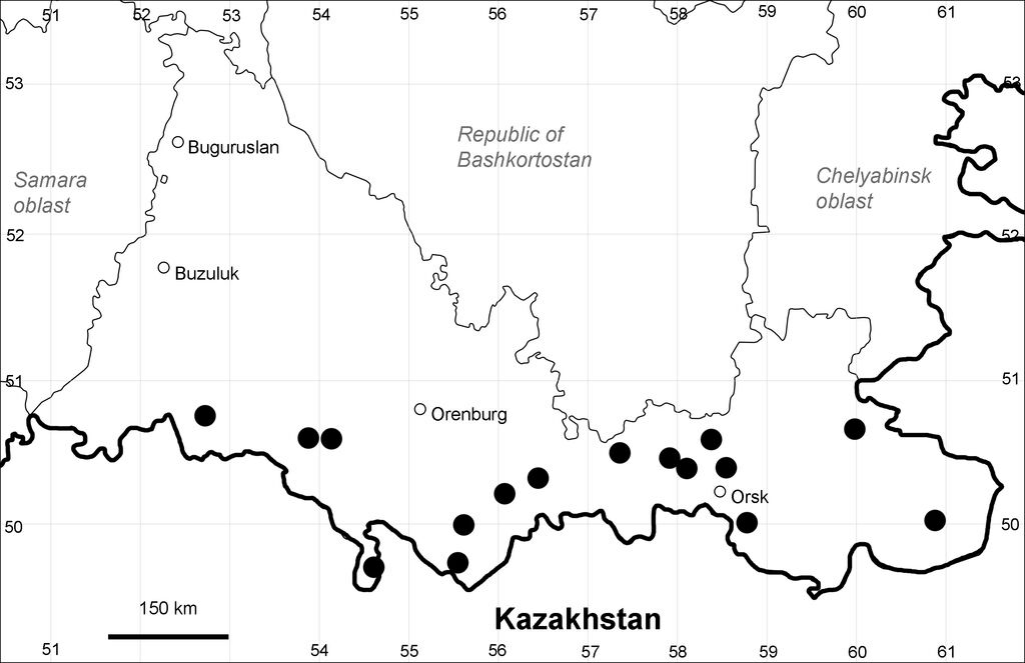
Distribution map of *Camphorosmamonspeliaca*.

Orenburg Region (southern districts): Adamovka, Akbulak, Belyaevka, Dombarovskii, Gai, Ilek, Kuvandyk, Orenburg, Sol'-Iletsk, Svetlyi, Tashla Districts (Fig. 56).

Previous reports: Iljin (1930), Ryabinina (1998), Knjazev (2009).

The species occurs on saline lands. It is frequent in the Region.

The species is distributed in South and Eastern (southern part) Europe and Central Asia.

#### Camphorosma songorica

Bunge

97394883-1FA0-5A72-AE01-21F18E07B383

urn:lsid:ipni.org:names:164628-1

***Camphorosmasongorica*** Bunge, Trudy Imp. S.-Peterburgsk. Bot. Sada 6(2): 415 (1879).
– *Camphorosmaannua* auct.: Iljin (1930).

##### Native status

Native.

##### Conservation status

Least Concern (LC).

EOO 91,000 km^2^, AOO 80 km^2^. The species was found in many localities in all parts of the territory. No decline was observed or projected.

##### Distribution

**Figure 57.**
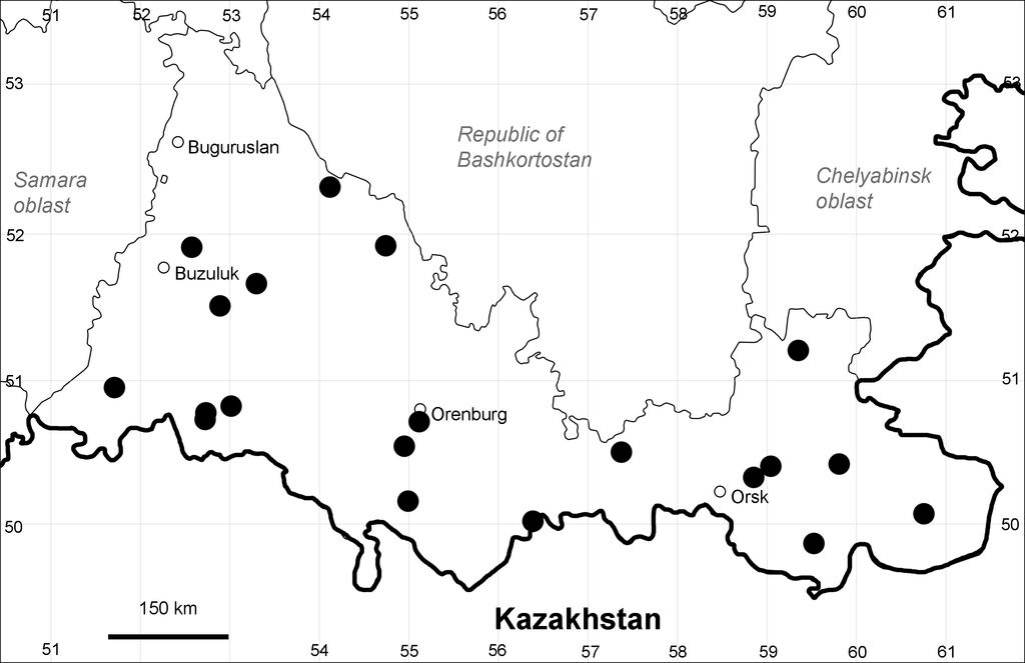
Distribution map of *Camphorosmasongorica*.

Orenburg Region (whole territory): Adamovka, Akbulak, Buzuluk, Dombarovka, Krasnaya Gvardia, Kuvandyk, Kvarkeno, Novoorsk, Orenburg, Pervomaiskii, Sharlyk, Sol'-Iletsk, Svetlyi, Tashla, Totskoe Districts (Fig. 57).

Previous reports: Iljin (1930), as *C.annua*; Ryabinina (1998), Knjazev (2009).

The species occurs on saline lands. It is common in the Region.

The species is distributed in Eastern Europe, Kazakhstan and western Siberia.

#### Sedobassia sedoides

(Pall.) Freitag & G.Kadereit

05109DD4-6F80-58DE-AC9F-C180BEDC93E2

urn:lsid:ipni.org:names:77110741-1

***Sedobassiasedoides*** (Pall.) Freitag & G.Kadereit, Taxon 60(1): 72 (2011) ≡ *Salsolasedoides* Pall., Reise 1: 492 (1771), nom. cons. ≡ *Bassiasedoides* (Pall.) Asch. in Schweinfurth, Beitr. Fl. Aethiop. 1: 187 (1867).

##### Native status

Native.

##### Conservation status

Least Concern (LC).

EOO 120,000 km^2^, AOO 170 km^2^. The species is one of the most common plants in saline sites, showing no decline.

##### Distribution

**Figure 58.**
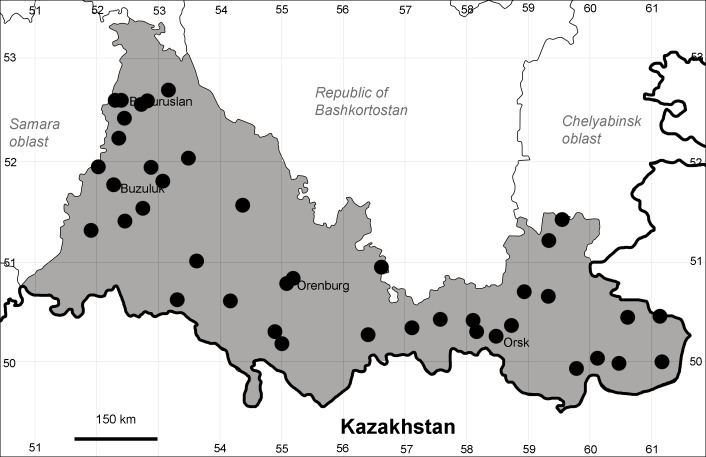
Distribution map of *Sedobassiasedoides*, confirmed (points) and projected (shade) occurrence.

Orenburg Region (whole territory): Adamovka, Aleksandrovka, Asekeevka, Belyaevka, Buguruslan, Buzuluk, Gai, Grachevka, Ilek, Krasnaya Gvardia, Kurmanaevka, Kuvandyk, Kvarkeno, Novoorsk, Novosergievka, Orenburg, Saraktash, Sol'-Iletsk, Svetlyi, Totskoe, Yasnyi Districts, Orsk Town (Fig. 58).

Previous reports: Iljin (1930), Ryabinina (1998), Knjazev (2009).

The species occurs on saline substrates. It is common in the Region.

The species is distributed as native in Central and Eastern (southern part) Europe, Kazakhstan, western China (Sennikov and Freitag 2014). It is known as a rare alien in the forest zone of Europe.

#### Spirobassia hirsuta

(L.) Freitag & G.Kadereit

58858E11-348C-53DD-9A31-E98923255203

urn:lsid:ipni.org:names:77110313-1

***Spirobassiahirsuta*** (L.) Freitag & G.Kadereit, Taxon 60(1): 71 (2011) ≡ *Chenopodiumhirsutum* L., Sp. Pl. 1: 221 (1753) ≡ *Bassiahirsuta* (L.) Asch. in Schweinfurth, Beitr. Fl. Aethiop. 1: 187 (1867).

##### Native status

Native.

##### Conservation status

Least Concern (LC).

EOO 27,800 km^2^, AOO 12 km^2^. The species has a restricted distribution in the south-eastern part of Orenburg Region. However, its populations are confined to saline lands, which are not threatened by anthropogenic disturbance and no actual protection is consequently required.

##### Distribution

**Figure 59.**
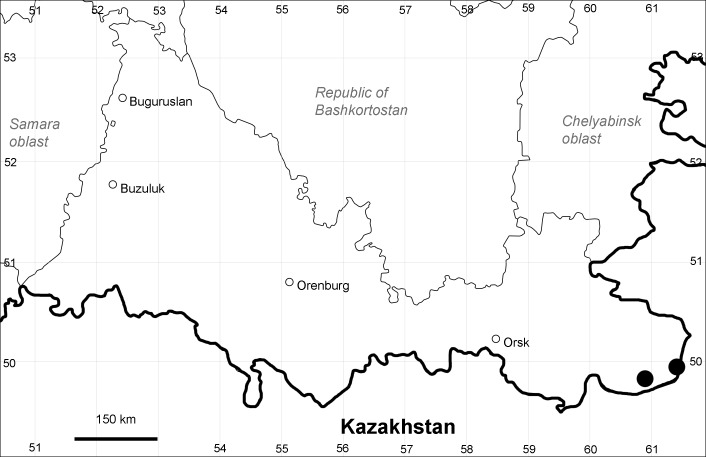
Distribution map of *Spirobassiahirsuta*.

Orenburg Region: Svetlyi District (Fig. 59).

Previous reports: Knjazev (2009).

The species occurs on saline lands. It is very rare in the eastern part of the Region.

The species is distributed as native in Western and Southern Europe (sea shores), south-west Siberia and Kazakhstan. It is known as alien in North America (eastern part).

#### Caroxylon laricinum

(Pall.) Tzvelev

DCE11AA8-8993-5B17-8CCE-29E4F488F66B

urn:lsid:ipni.org:names:972505-1

***Caroxylonlaricinum*** (Pall.) Tzvelev, Ukr. Bot. Zhurn. 50(1): 81 (1993) ≡ *Salsolalaricina* Pall., Ill: 21 (1803).

##### Native status

Native.

##### Conservation status

Least Concern (LC).

EOO 114,400 km^2^, AOO 72 km^2^. The species is widely distributed in the southern parts of the territory, showing no decline.

##### Distribution

**Figure 60.**
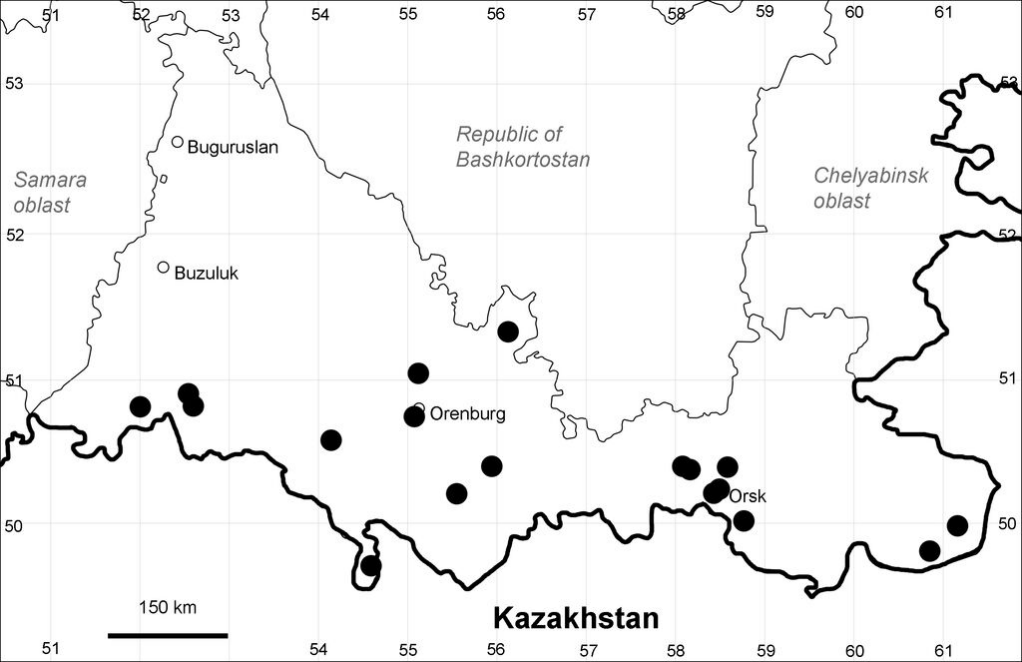
Distribution map of *Caroxylonlaricinum*.

Orenburg Region: Akbulak, Belyaevka, Dombarovka, Gai, Orenburg, Pervomaiskii, Severnoe, Sol'-Iletsk, Svetlyi, Tashla, Tyulgan Districts, Orsk Town (Fig. 60).

Previous reports: Iljin (1930), Ryabinina (1998), Knjazev (2009).

The species occurs in steppes, on sands and limestones. It is frequent in the Region.

The species is distributed in Russia, Ukraine (southern part), Kazakhstan, West Asia (Turkey).

#### Nanophyton erinaceum

(Pall.) Bunge

8E964E91-70AE-5EEE-97AB-1FC7F907852A

urn:lsid:ipni.org:names:166172-1

***Nanophytonerinaceum*** (Pall.) Bunge, Mém. Ac. Sci. Petersb., ser. 7, 4(11): 51 (1862) ≡ *Polycnemumerinaceum* Pall., Ill. Pl.: 58 (1803).
The species was originally described from Orenburg Region.

##### Native status

Native.

##### Conservation status

Vulnerable (VU): A3(d), C1. Currently under protection, category 3 (Belov 2019).

EOO 28,600 km^2^, AOO 76 km^2^. The species was protected because of its rarity on the northern margin of the species distribution and because of the vulnerability of its habitats (limestone and other calcareous deposits are actively developed) (Ryabinina 1995). Its currently known distribution is much more extensive than previously believed (Belov 2019), but the limited size of its populations and the vulnerability of its habitats to anthropogenic pressure suggest that its protection status is appropriate.

##### Distribution

**Figure 61.**
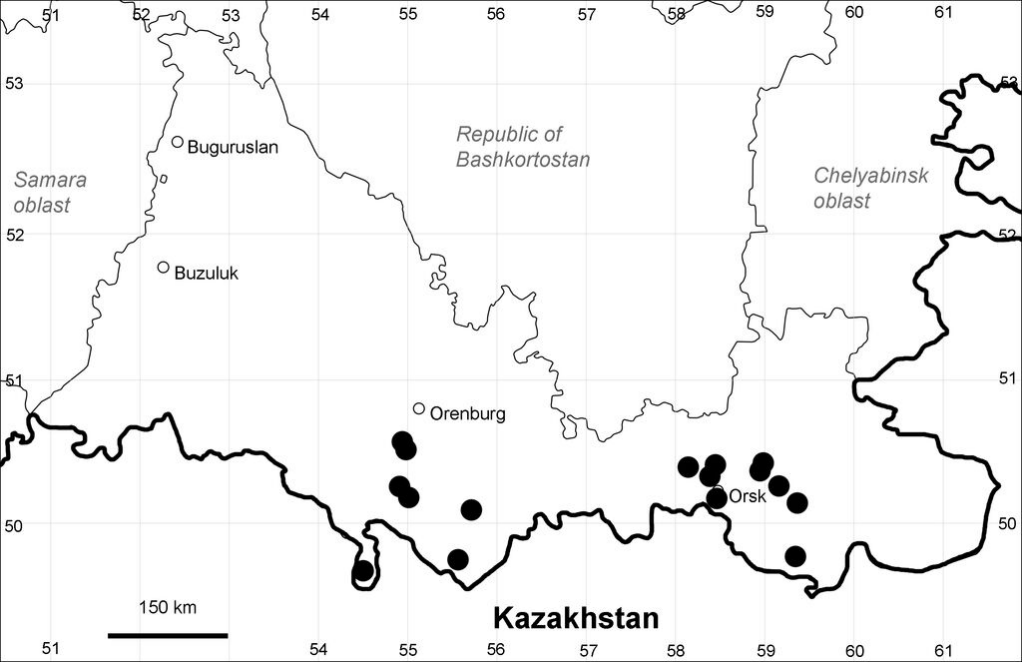
Distribution map of *Nanophytonerinaceum*.

Orenburg Region (southern districts): Akbulak, Belyaevka, Dombarovka, Gai, Novoorsk, Orenburg, Sol'-Iletsk, Yasnyi Districts, Orsk Town (Fig. 61).

Previous reports: Iljin (1930), Ryabinina (1998), Knjazev (2009).

The species occurs in petrophytic steppes, on chalk and limestone. It has a restricted distribution in the Region.

The species is distributed in Eastern Europe (south-eastern part), Kazakhstan, western China. In Russia, the species occurs only in Orenburg Region.

#### Ofaiston monandrum

(Pall.) Moq.

4DFD9129-876B-5215-A4BC-84341FDD9ECA

urn:lsid:ipni.org:names:166316-1

***Ofaistonmonandrum*** (Pall.) Moq. in DC., Prodr. 13(2): 203 (1849) ≡ *Salsolamonandra* Pall., Reise 3: 607 (1776).

##### Native status

Native.

##### Conservation status

Least Concern (LC).

EOO 33,600 km^2^, AOO 44 km^2^. The species is not rare in the eastern part of the territory.

##### Distribution

**Figure 62.**
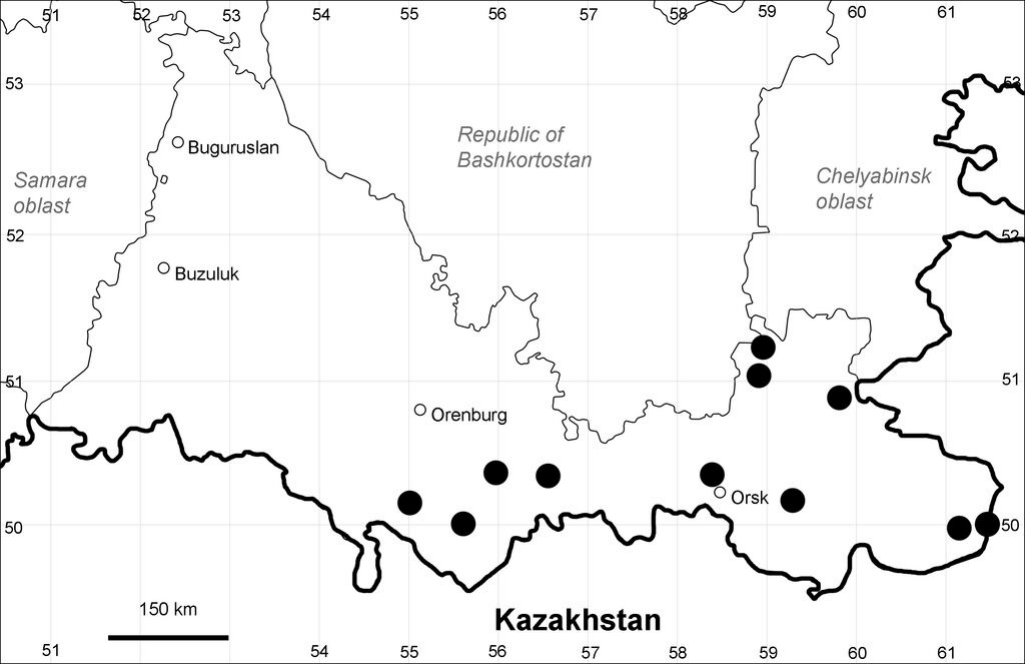
Distribution map of *Ofaistonmonandrum*.

Orenburg Region (central and eastern districts): Adamovka, Akbulak, Belyaevka, Gai, Kvarkeno, Novoorsk, Sol'-Iletsk, Svetlyi Districts (Fig. 62). Northern limit of the species distribution.

Previous reports: Ryabinina (1998), Knjazev (2009).

The species occurs on saline lands. It is frequent in the Region.

The species is distributed in Eastern Europe (southern part), West Siberia, Kazakhstan, Uzbekistan (near Aral Sea).

#### Petrosimonia brachyphylla

(Bunge) Iljin

03E5C87D-1F6B-5729-A357-F4960C8C9465

urn:lsid:ipni.org:names:166360-1

***Petrosimoniabrachyphylla*** (Bunge) Iljin, Materialy Komissii Exped. Issledovaniy Akad. Nauk 26(2): 282 (1930) ≡ Petrosimoniacrassifoliavar.brachyphylla Bunge, Mém. Acad. Imp. Sci. Saint-Pétersbourg, Sér. 7, 4(11): 56 (1862).

##### Native status

Native.

##### Conservation status

Least Concern (LC).

EOO 6,400 km^2^, AOO 16 km^2^. The species is rare in the region, but not immediately threatened.

##### Distribution

**Figure 63.**
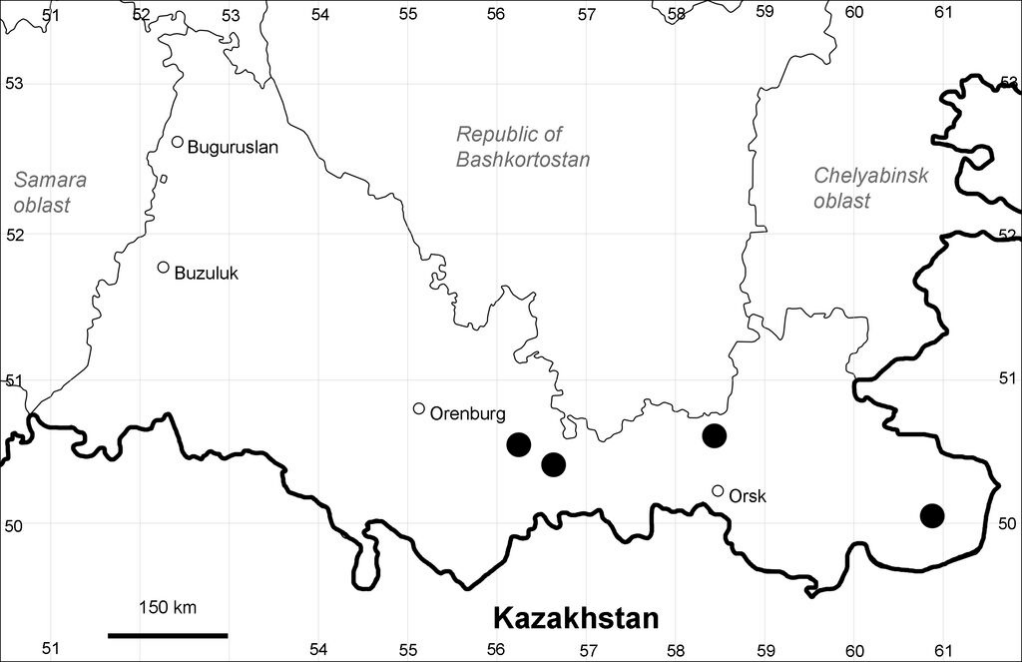
Distribution map of *Petrosimoniabrachyphylla*.

Orenburg Region: Belyaevka, Gai, Saraktash, Svetlyi Districts (Fig. 63).

Previous reports: Mavrodiev and Sukhorukov (2000), Knjazev (2009).

The species occurs on saline lands. It is rare in the Region.

The species is distributed in Eastern Europe (south-eastern part), Kazakhstan, western China.

#### Petrosimonia litwinowii

Korsh.

7D7DAF23-6403-5F62-9721-0E7A3B333771

urn:lsid:ipni.org:names:166369-1

***Petrosimonialitwinowii*** Korsh., Tent. Fl. Ross. Orient.: 358 (1898).
The species was originally described from Orenburg Region.

##### Native status

Native.

##### Conservation status

Least Concern (LC).

EOO 80,600 km^2^, AOO 80 km^2^. The species is common in the territory and shows no decline.

##### Distribution

**Figure 64.**
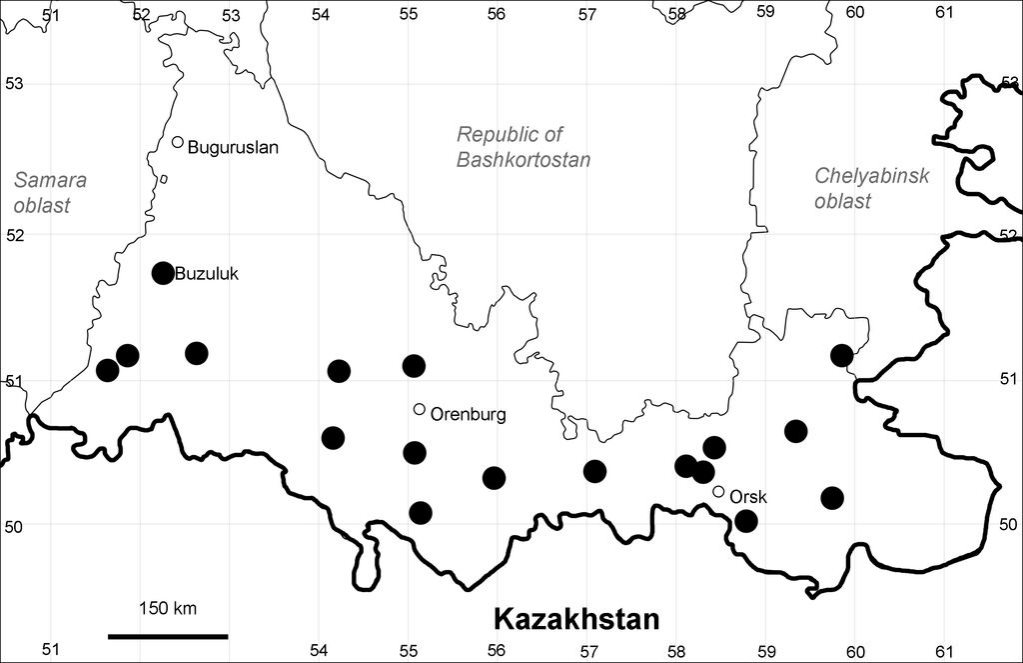
Distribution map of *Petrosimonialitvinovii*.

Orenburg Region: Adamovka, Aleksandrovka, Belyaevka, Dombarovka, Gai, Kvarkeno, Kurmanaevka, Kuvandyk, Novoorsk, Orenburg, Pervomaiskii, Sol'-Iletsk, Svetlyi, Totskoe, Yasnyi Districts (Fig. 64).

Previous reports: Ryabinina (1998), Knjazev (2009).

The species occurs on saline lands. It is common in the Region.

The species is distributed in Russia and Kazakhstan.

#### Petrosimonia monandra

(Pall.) Bunge

843663A6-B5D9-519F-B653-3DD6BFB3A85E

urn:lsid:ipni.org:names:166370-1

***Petrosimoniamonandra*** (Pall.) Bunge, Anabas. Rev.: 53 (1862) ≡ *Polycnemummonandrum* Pall., Reise 1: 483 (1771).

##### Native status

Native.

##### Conservation status

Data Deficient (DD).

A threatened category has not been assigned due to the absence of recent collections.

##### Distribution

**Figure 65.**
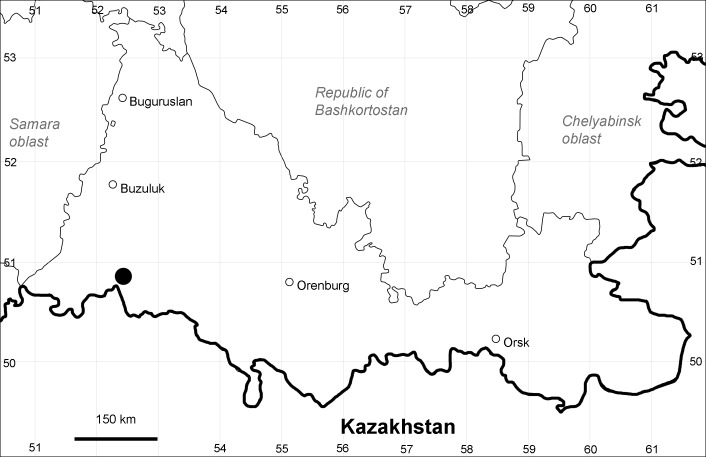
Distribution map of *Petrosimoniamonandra*.

Orenburg Region: Tashla District (Fig. 65).

Previous reports: Iljin (1930), Ryabinina (1998), Knjazev (2009).

The species occurs in steppes and on saline substrates. It is very rare in the Region, known from a single collection dated the 1920s.

The species is distributed in Russia and Kazakhstan.

#### Petrosimonia triandra

(Pall.) Simonk.

C64F11DE-18FA-5E3C-BB6C-C7A2BF08A144

urn:lsid:ipni.org:names:166376-1

***Petrosimoniatriandra*** (Pall.) Simonk., Enum. Pl. Transsilv.: 466 (1866) ≡ *Polycnemumtriandrum* Pall., Reise 1: 483 (1771).
= *Polycnemumvolvox* Pall., Ill. Pl.: 60 (1803) ≡ *Petrosimoniavolvox* (Pall.) Bunge, Anabas. Rev.: 54 (1862).

##### Native status

Native.

##### Conservation status

Least Concern (LC).

EOO 34,700 km^2^, AOO 44 km^2^. The species is common in the southern part of the territory and shows no decline.

The species is currenty listed as legally protected in Orenburg Region (category 2) because of its presumed rarity and restricted distribution (four localities were known at the time) (Belov 2019). According to our data, the species is widely distributed in the southern part of Orenburg Region and its wider occurrence is expected. No legal protection is needed.

##### Distribution

**Figure 66.**
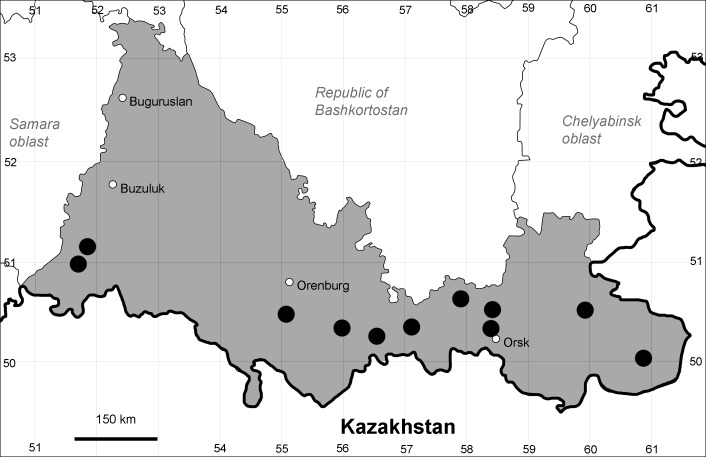
Distribution map of *Petrosimoniatriandra*, confirmed (points) and projected (shade) occurrence.

Orenburg Region: Adamovka, Belyaevka, Gai, Kurmanaevka, Kuvandyk, Pervomaiskii, Sol'-Iletsk, Svetlyi Districts (Fig. 66).

Previous reports: Knjazev (2009).

The species occurs in steppes and on saline lands. It is common in the southern part of the Region.

The species is distributed in Central, South and Eastern Europe, the Caucasus, western Iran, Central Asia (Kazakhstan).

#### Pyankovia brachiata

(Pall.) Akhani & Roalson

8216EFE5-F2B2-56D9-98E3-D9DFE032746A

urn:lsid:ipni.org:names:77089207-1

***Pyankoviabrachiata*** (Pall.) Akhani & Roalson, Int. J. Pl. Sci. 168(6): 949 (2007) ≡ *Salsolabrachiata* Pall., Ill. Pl. 30 (1803) ≡ *Climacopterabrachiata* (Pall.) Botsch., Sborn. Rabot Akad. Sukachevu: 114 (1956).

##### Native status

Native.

##### Conservation status

Least Concern (LC).

EOO 37,400 km^2^, AOO 36 km^2^. The species is common in the central and eastern parts of the territory and shows no decline.

##### Distribution

**Figure 67.**
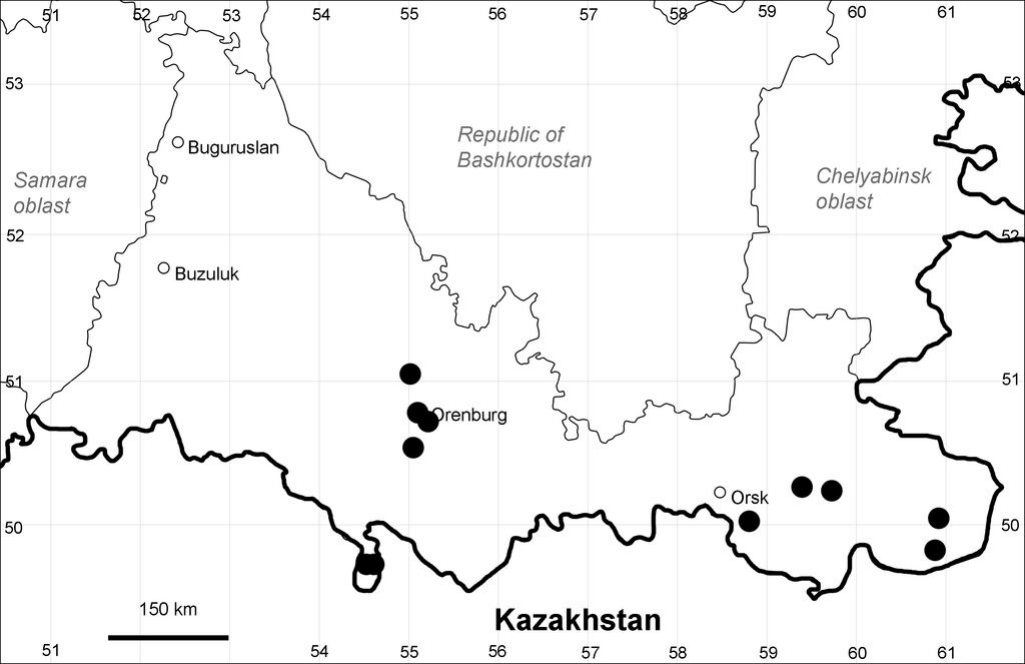
Distribution map of *Pyankoviabrachiata*.

Orenburg Region: Novoorsk, Orenburg, Sakmara, Sol'-Iletsk, Svetlyi, Yasnyi Districts (Fig. 67).

Previous reports: Knjazev (2009).

The species occurs in steppes, on saline lands and also in human-made habitats. It is common in the southern part of the Region.

The species is distributed in Eastern Europe (south-eastern part) and Central Asia.

#### Anabasis cretacea

Pall.

FB46C7E8-C7C6-56D9-A028-146E8517B7FC

urn:lsid:ipni.org:names:163406-1

***Anabasiscretacea*** Pall., Reise 1: 493 (1771).

##### Native status

Native.

##### Conservation status

Vulnerable (VU): A3(d), C1. Currently under protection, category 3 (Belov 2019).

EOO 44,800 km^2^, AOO 16 km^2^. Before the recent period of the botanical exploration, the species was known in three districts only and considered to have a limited distribution in Orenburg Region (Ryabinina 1998). Since then, further localities were discovered (Golovanov et al. 2018) and the distribution mapped here is even greater.

The species occurs exclusively on open calcareous substrates (chalk, limestone, marl, gypsaceous denudations) in small populations (Ryabinina 1998, Belov 2019). Although no apparent large-scale decline of its populations was observed, its habitats are fragile and require legal protection because of their being susceptible to anthropogenic pressure (by visiting and pasturing) and destruction by mining, which assumes projected decline of its populations in the absence of protection. Similarly, the species is protected in the neighbouring territories: Bashkortostan (Martynenko 2021) and Chelyabinsk Region (Lagunov 2017).

##### Distribution

**Figure 68.**
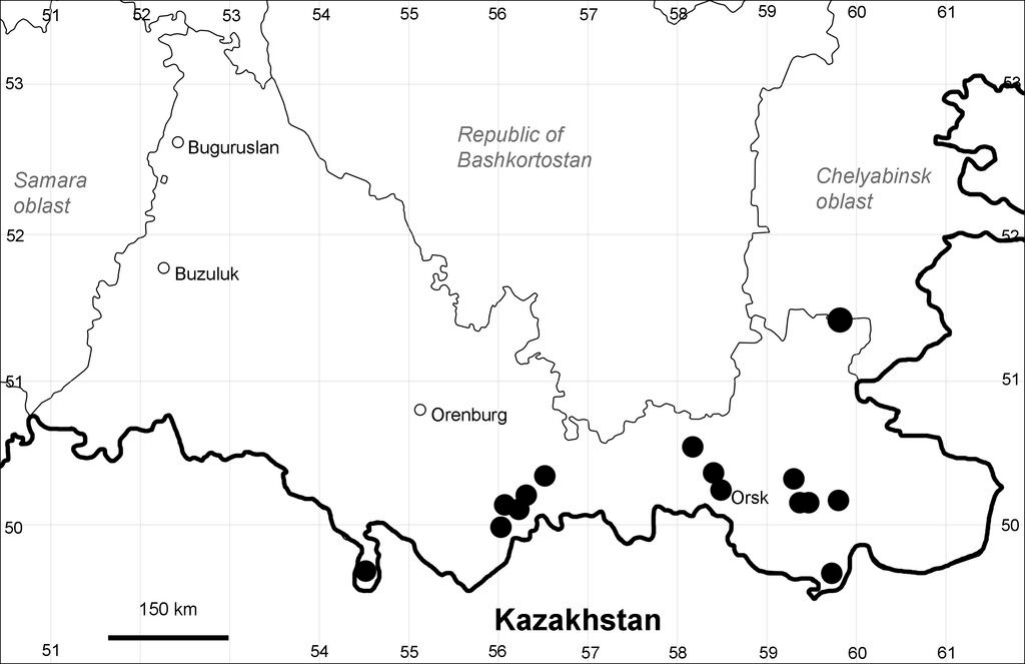
Distribution map of *Anabasiscretacea*.

Orenburg Region: Akbulak, Belyaevka, Dombarovka, Gai, Novoorsk, Sol'-Iletsk, Yasnyi Districts, Orsk Town (Fig. 68).

Previous reports: Ryabinina (1998), Knjazev (2009).

The species occurs in petrophytic steppes, on limestone. It has a limited distribution in the Region.

The species is distributed in Eastern Europe (south-eastern part), Siberia (south-western part), Kazakhstan, western China (Xinjiang).

#### Anabasis salsa

(Ledeb.) Benth. ex Volkens

C846BE9B-592E-5990-88B1-2884D6AF6422

urn:lsid:ipni.org:names:163460-1

***Anabasissalsa*** (Ledeb.) Benth. ex Volkens in Engler & Prantl, Nat. Pflanzenfam. 3, 1a: 87 (1893) ≡ *Brachylepissalsa* Ledeb., Icon. Pl. Fl. Ross. 1: 12 (1829).

##### Native status

Native.

##### Conservation status

Vulnerable (VU): C1. Currently under protection, category 3 (Belov 2019).

EOO 43,300 km^2^, AOO 68 km^2^. The species has been placed under legal protection because of its rarity on the northern margin of the species distribution, because its population size was estimated at less than 1000 mature individuals and because its populations were threatened by grazing. Its currently known distribution is much more extensive than previously believed (Belov 2019) and its habitats (saline lands) are not threatened by human activities. For these reasons, we suggest that its current protection status is inappropriate and should be changed to Least Concern.

##### Distribution

**Figure 69.**
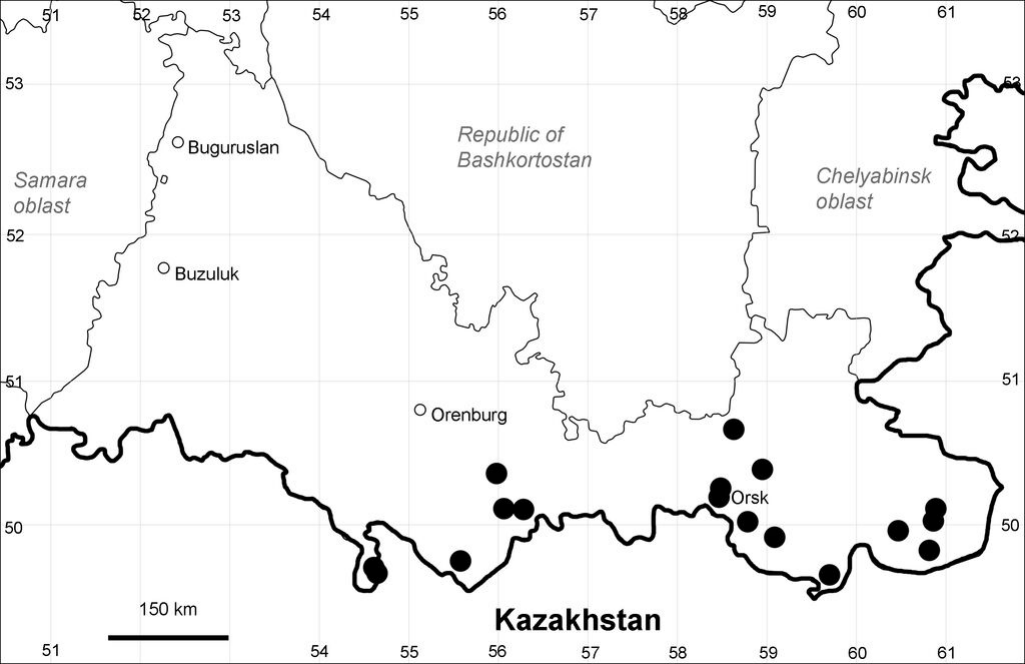
Distribution map of *Anabasissalsa*.

Orenburg Region: Akbulak, Belyaevka, Dombarovka, Gai, Novoorsk, Sol'-Iletsk, Svetlyi, Yasnyi Districts, Orsk Town (Fig. 69).

Previous reports: Knjazev (2009).

The species occurs in petrophytic steppes, on saline linds. It has a limited distribution in the Region.

The species is distributed in Eastern Europe (south-eastern part), the Caucasus (Azerbaijan), south-western Siberia, Kazakhstan, western China (Xinjiang), western Mongolia.

#### Arthrophytum lehmannianum

Bunge ex Litv.

3D98CDB4-ED22-5062-9CFE-6A3B970EF555

urn:lsid:ipni.org:names:163594-1

***Arthrophytumlehmannianum*** Bunge ex Litv. in Trudy Bot. Muz. Imp. Akad. Nauk 11: 33 (1913).

##### Native status

Native.

##### Conservation status

Vulnerable (VU). D1.

The species is known from the only locality in Orenburg Region and Russia, isolated at the northern margin of its distribution area, where the only small population is known. It was observed in a petrophytic steppe on saline substrates, co-occurring with *Camphorosmamonspeliaca*, *Artemisianitrosa*, *Atriplexcana* etc. (Stepanova and Safronova 2019). The species population is situated next to the road and is in danger of further development of the road network. For this reason, legal protection is recommended.

##### Distribution

**Figure 70.**
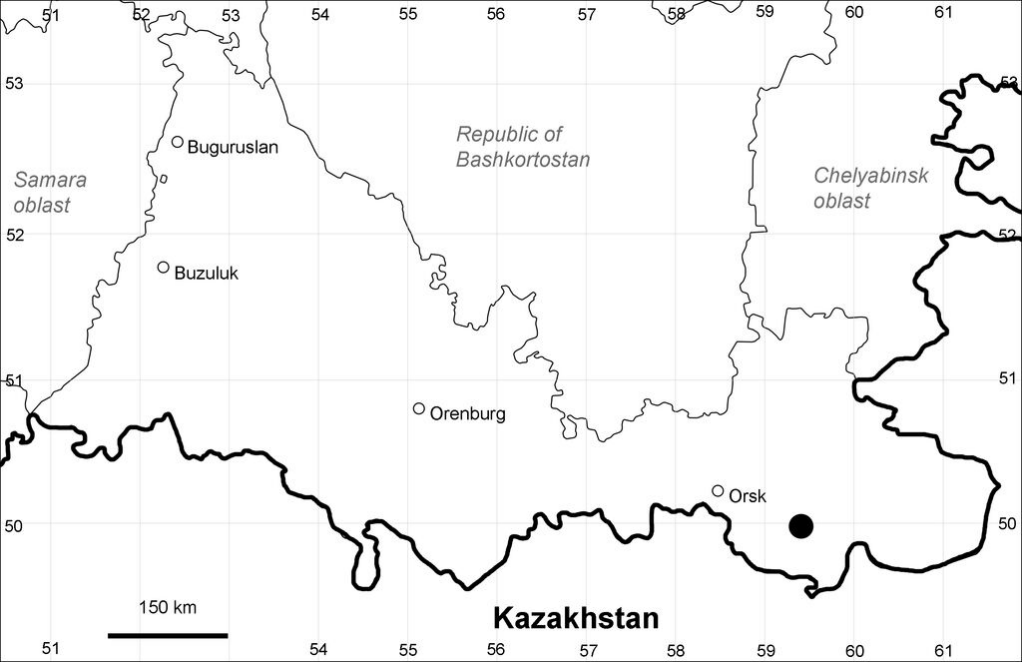
Distribution map of *Arthrophytumlehmannianum*.

Orenburg Region: Yasnyi District (Fig. 70).

Previous reports: Stepanova and Safronova (2019).

The species occurs in petrophytic steppes. It is very rare in the Region.

The species is distributed in Eastern Europe (south-eastern part), Kazakhstan, western China. The record in Yasnyi District belongs to the only population found in Russia (Stepanova and Safronova 2019).

#### Halogeton glomeratus

(M.Bieb.) Ledeb.

02478A50-8512-5A61-AFC0-375D370D66DF

urn:lsid:ipni.org:names:60448682-2

***Halogetonglomeratus*** (M.Bieb.) Ledeb., Icon. Pl. Fl. Ross. 1: 10 (1829) ≡ *Anabasisglomerata* M.Bieb., Mém. Soc. Imp. Naturalistes Moscou 1(ed. 2): 110 (1811).

##### Native status

Native.

##### Conservation status

Least Concern (LC).

EOO 1,000 km^2^, AOO 12 km^2^. The species is found in a few localities in the south-eastern part of the territory and shows no tendency to decline.

##### Distribution

**Figure 71.**
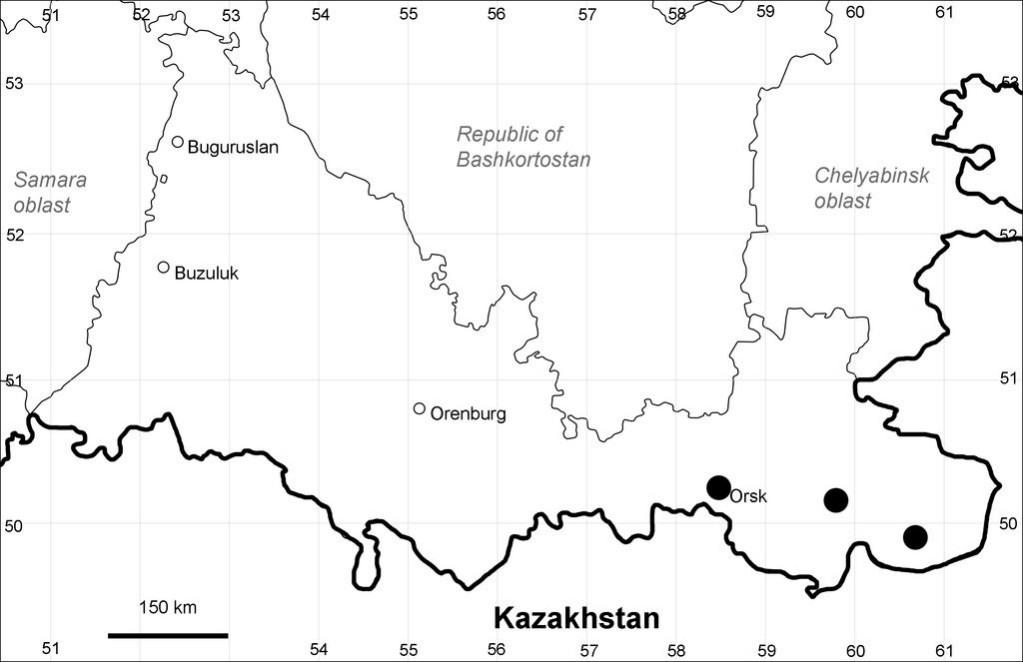
Distribution map of *Halogetonglomeratus.*

Orenburg Region (south-eastern districts): Svetlyi, Yasnyi Districts, Orsk Town (Fig. 71).

Previous reports: Knjazev (2009).

The species occurs in steppes, on saline lands and in human-made habitats. It is found sporadically in the eastern part of the Region.

The species is distributed as native in Eastern Europe (southern Ural), Siberia, Central Asia, North Himalaya, Mongolia, western China. It is known as alien in North America.

#### Salsola collina

Pall.

505664FD-DB4A-5B87-ADB3-9704A0E4622F

urn:lsid:ipni.org:names:166737-1

***Salsolacollina*** Pall., Ill. Pl.: 34 (1803) ≡ *Kalicollina* (Pall.) Akhani & Roalson, Int. J. Pl. Sci. 168(6): 946 (2007).

##### Native status

Native.

##### Conservation status

Least Concern (LC).

EOO 107,700 km^2^, AOO 108 km^2^. The species is very common in the whole territory and shows no tendency to decline.

##### Distribution

**Figure 72.**
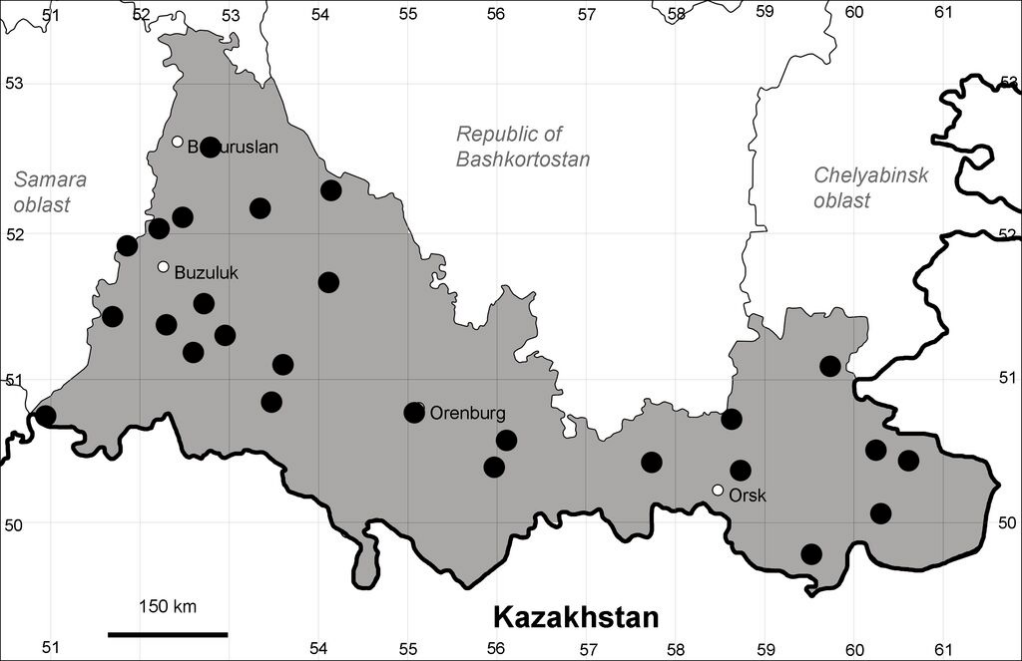
Distribution map of *Salsolacollina*, confirmed (points) and projected (shade) occurrence.

Orenburg Region (whole territory): Adamovka, Aleksandrovka, Asekeevka, Belyaevka, Buguruslan, Buzuluk, Dombarovskii, Gai, Kurmanaevka, Kvarkeno, Matveevka, Novosergievka, Orenburg, Pervomaiskii, Ponomarevka, Saraktash, Sorochinsk, Totskoe, Yasnyi Districts (Fig. 72).

Previous reports: Iljin (1930), Ryabinina (1998), Knjazev (2009).

The species occurs in steppes, on saline lands and sands and in human-disturbed sites. It is common in the Region.

The species is distributed as native in Eastern Europe (south-eastern part) and across most of the arid Asia (Mongolia, China, Kyrgyzstan, mountainous parts of Kazakhstan, Tajikistan, southwards up to mountainous deserts of Karakoram). It is known as alien in the forest and forest-steppe zones of Eurasia.

#### Salsola rosacea

L.

C8583064-1A97-5052-B2F6-B3F33305FBBC

urn:lsid:ipni.org:names:167034-1

***Salsolarosacea*** L., Sp. Pl. 1: 222 (1753) ≡ *Kalirosaceum* (L.) Moench, Suppl. Meth.: 115 (1802).

##### Native status

Native.

##### Conservation status

Vulnerable (VU). D1.

The species is locally known only from Verblyuzhka Mount in a low number of individuals. This area is overgrazed due to the presence of several small livestock farms.

##### Distribution

**Figure 73.**
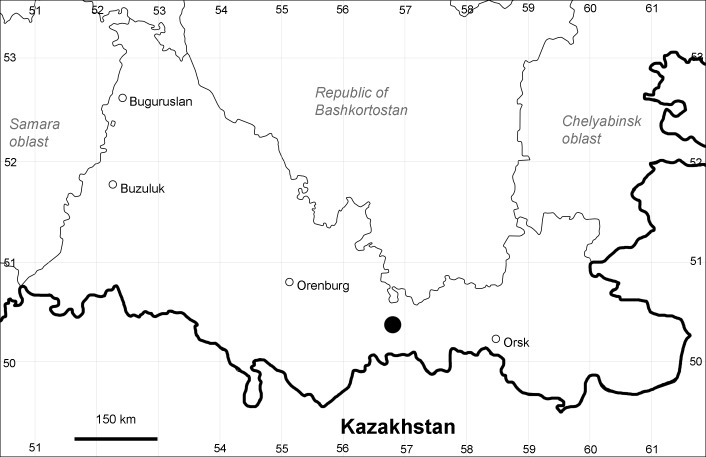
Distribution map of *Salsolarosacea*.

Orenburg Region: Belyaevka District (Fig. 73).

Previous reports: Knjazev (2009).

The species occurs in petrophytic steppes.

It is distributed in Eastern Europe (south-eastern part), southern Siberia, Central Asia. So far, this is the only record of *Salsolarosacea* in Europe, at the very limit of the European territory.

#### Salsola tamariscina

Pall.

5BEBB259-417E-50A7-8648-714B6A42F478

urn:lsid:ipni.org:names:167118-1

***Salsolatamariscina*** Pall., Ill. Pl.: 33 (1803) ≡ *Kalitamariscina* (Pall.) Akhani & Roalson, Int. J. Pl. Sci. 168(6): 946 (2007).

##### Native status

Native.

##### Conservation status

Least Concern (LC).

EOO 117,300 km^2^, AOO 104 km^2^. The species is common in the whole territory and shows no tendency to decline.

##### Distribution

**Figure 74.**
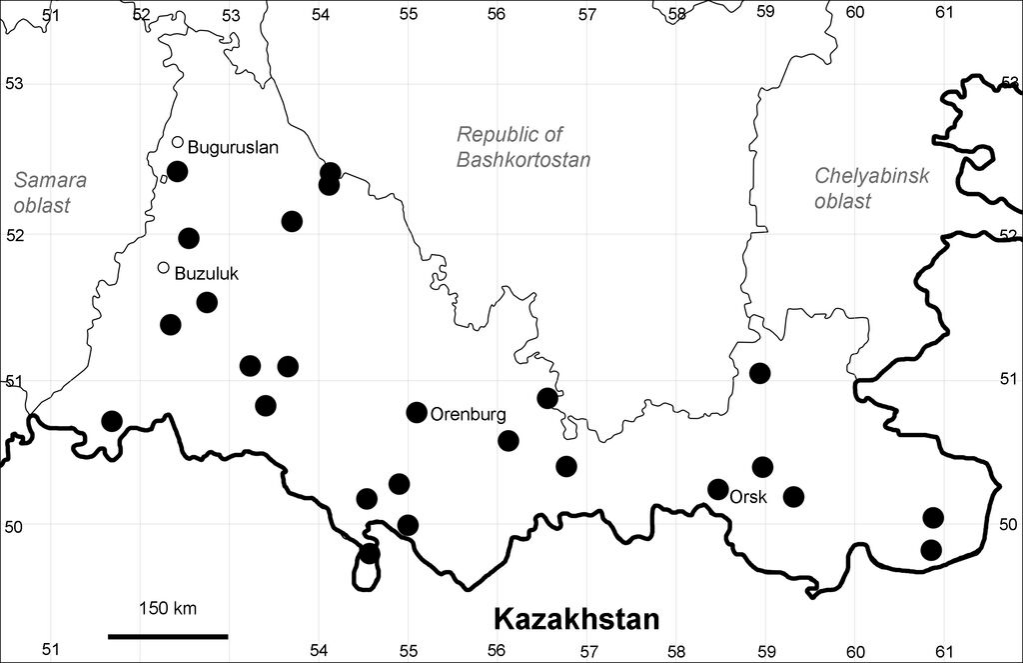
Distribution map of *Salsolatamariscina*.

Orenburg Region (whole territory): Belyaevka, Buguruslan, Buzuluk, Krasnaya Gvardia, Kvarkeno, Novoorsk, Novosergievka, Orenburg, Pervomaiskii, Ponomarevka, Saraktash, Sol'-Iletsk, Svetlyi, Totskoe Districts, Orsk Town (Fig. 74).

Previous reports: Iljin (1930), Ryabinina (1998), Knjazev (2009).

The species occurs in steppes, on saline lands and limestone. It is common in the Region.

The species is distributed in Eastern Europe (southern part), Kazakhstan, Uzbekistan, western China (Xinjiang).

#### Salsola tragus

L.

8AF18280-5AAE-506C-83B0-FDCD3672F042

urn:lsid:ipni.org:names:307757-2

**Salsolatragus** L., Cent. Pl. 2: 13 (1756) ≡ *Kalitragus* (L.) Scop., Fl. Carniol., ed. 2, 1: 175 (1772).
= *Salsolapestifer* A.Nelson, New Man. Bot. Centr. Rocky Mt., ed. 2: 169 (1909).
= *Salsolaruthenica* Iljin, Sornye Rast. SSSR 2: 137 (1934).
– *S.australis* auct.: Ryabinina (1998).

##### Native status

Native.

##### Conservation status

Least Concern (LC).

EOO 62,800 km^2^, AOO 40 km^2^. The species is common in the whole territory and shows no tendency to decline. Its actual distribution is predicted to be much wider than currently confirmed.

##### Distribution

**Figure 75.**
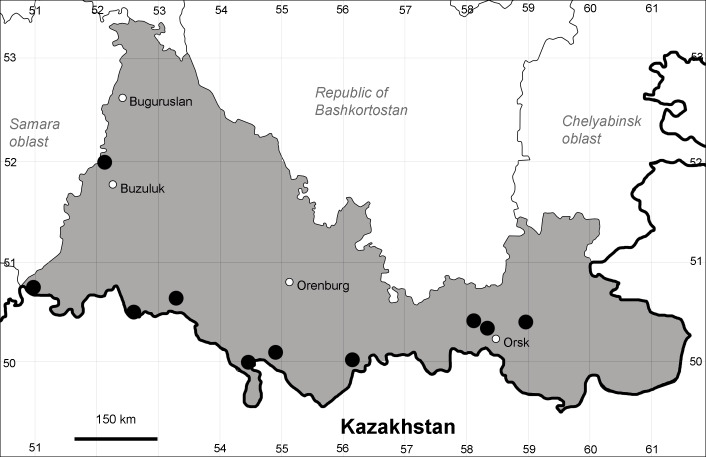
Distribution map of *Salsolatragus*, confirmed (points) and projected (shade) occurrence.

Orenburg Region: Akbulak, Buzuluk, Gai, Ilek, Novoorsk, Pervomaiskii, Sol'-Iletsk, Tashla Districts, Orsk Town (Fig. 75).

Previous reports: Ryabinina (1998), Knjazev (2009).

The species occurs in steppes, on saline lands, sands and limestone and also in human-disturbed sites. It is frequent in the Region.

The species is distributed in Eurasia (extratropical). It is known as alien in North and South America.

#### Soda acutifolia

(Bunge) Mosyakin, Freitag & Rilke

8BC78036-162D-5BF8-9BA0-907830664A7F

urn:lsid:ipni.org:names:60477034-2

**Sodaacutifolia** (Bunge) Mosyakin, Freitag & Rilke, Isr. J. Pl. Sci. 64(1–2): 25 (2017) ≡ *Halogetonacutifolius* Bunge, Beitr. Fl. Russl.: 301 (1852).
= *Salsolamutica* C.A.Mey., Bull. Soc. Nat. Mosc. 27(2): 455 (1854).

##### Native status

Native.

##### Conservation status

Least Concern (LC).

EOO 66,700 km^2^, AOO 32 km^2^. The species is common in some perts of the territory. No decline is observed or projected.

##### Distribution

**Figure 76.**
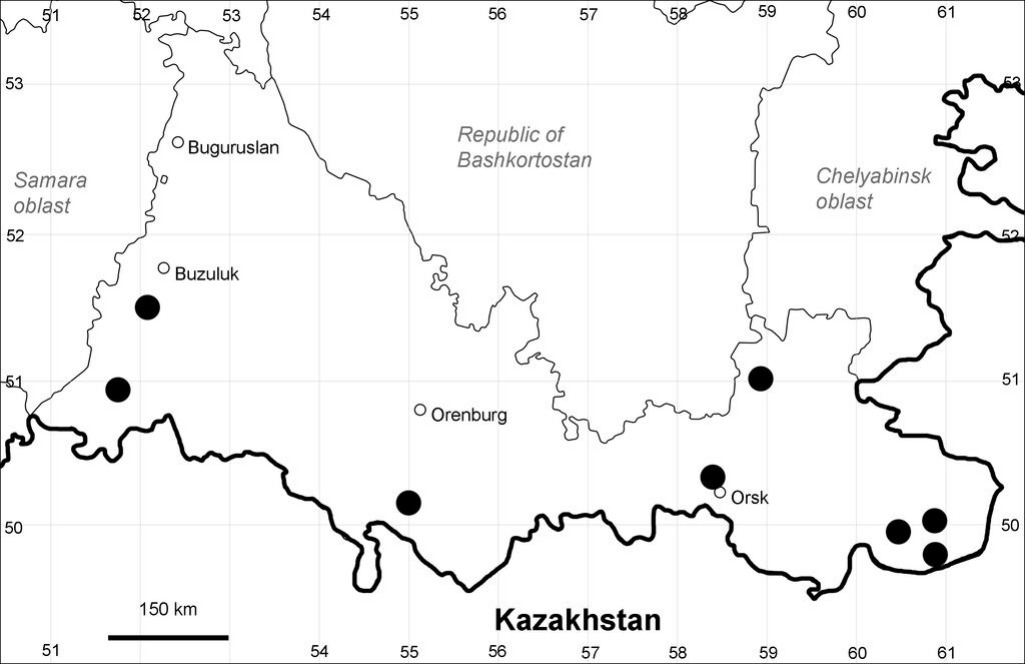
Distribution map of *Sodaacutifolia*.

Orenburg Region: Gai, Kurmanaevka, Kvarkeno, Pervomaiskii, Sol'-Iletsk, Svetlyi, Yasnyi Districts (Fig. 76).

Previous reports: Knjazev (2009).

The species occurs on saline soils. It is sporadically observed in the Region.

The species is distributed in Eastern Europe (south-eastern part) and Kazakhstan.

#### Soda foliosa

(L.) Akhani

44463653-D3A1-5BC8-893C-6F6D59F5B3F0

urn:lsid:ipni.org:names:77215009-1

**Sodafoliosa** (L.) Akhani, Front. Pl. Sci. 11-546518: 30 (2020) ≡ *Anabasisfoliosa* L., Sp. Pl. 1: 223 (1753) ≡ *Salsolafoliosa* (L.) Schrad. ex Schult. in Roemer & Schultes, Syst. Veg., ed. 15[bis], 6: 235 (1820) ≡ *Neocaspiafoliosa* (L.) Tzvel., Ukr. Bot. Zhurn. 50(1): 81 (1993).

##### Native status

Native.

##### Conservation status

Least Concern (LC).

The species is found in two localities in the south-eastern part of the territory. Despite its limited distribution, its habitats (saline lands) are not under threat and the populations show no tendency to decline.

##### Distribution

**Figure 77.**
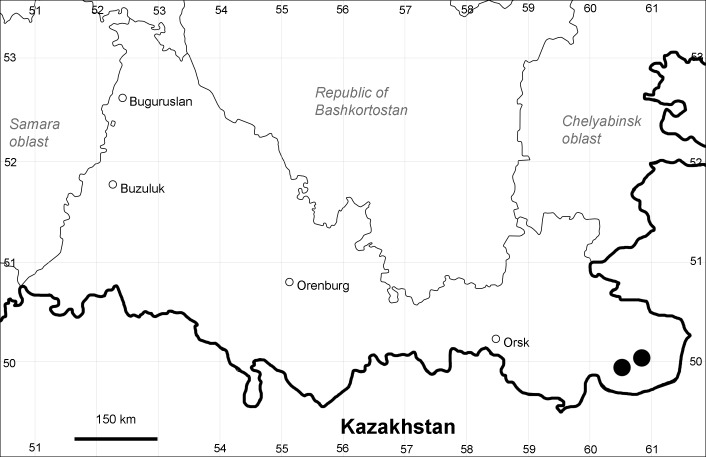
Distribution map of *Sodafoliosa*.

Orenburg Region: Svetlyi District (Fig. 77).

Previous reports: Iljin (1930), Ryabinina (1998), Knjazev (2009).

The species occurs on saline soils and limestones. It is sporadically observed in the easternmost part of the Region.

The species is distributed in Eastern Europe (south-eastern part), Kazakhstan, western China, Mongolia.

#### Soda inermis

(Moench) Fourr.

50868440-4CF9-5B85-A732-0376F1117763

urn:lsid:ipni.org:names:167375-1

***Sodainermis*** (Moench) Fourr., Ann. Soc. Linn. Lyon, sér. 2, 17: 145 (1869) ≡ *Salsolasoda* L., Sp. Pl. 1: 223 (1753) ≡ *Kaliinermis* Moench, Methodus: 331 (1794).
Kali inermis Moench is a legitimate name due to the simultaneous publication of *Kalisoda* Moench, nom. illeg. (Moench 1794).

##### Native status

Native.

##### Conservation status

Least Concern (LC).

EOO 30,600 km^2^, AOO 24 km^2^. The species has a sparse, but wide distribution in the territory, without apparent threats to its populations.

##### Distribution

**Figure 78.**
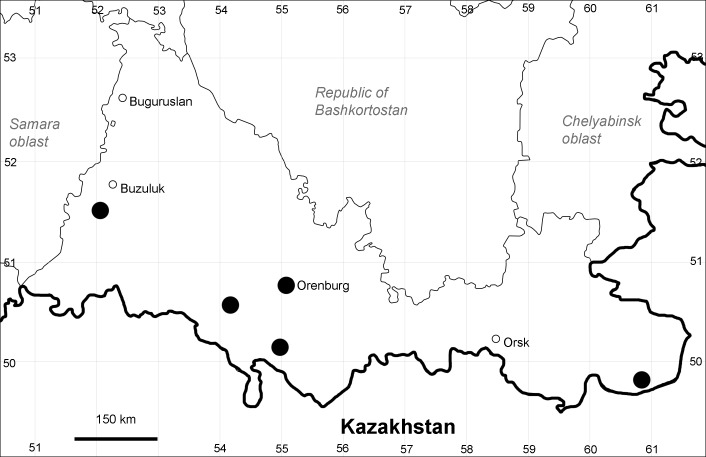
Distribution map of *Sodainermis*.

Orenburg Region: Kurmanaevka, Orenburg, Sol'-Iletsk, Svetlyi Districts (Fig. 78).

Previous reports: Iljin (1930), Ryabinina (1998), Knjazev (2009).

The species occurs on saline substrates. It is rare in the Region.

The species is distributed in Southern and Eastern Europe, Kazakhstan, West Asia, North Africa. It is known as alien in North and South America.

### Excluded taxa

#### Atriplex hortensis

L.

DBAECFC6-D2D5-50A0-A90F-39DCEF79CE58

##### Distribution

Some specimens of this species are known in the northern territories, for example, Sverdlovsk Region (SVER!) and Bashkortostan (LE!). In the steppe zone, *A.hortensis* needs a freshwater irrigation and the salinisation effect is damaging the seed germination of the species, thus making its spontaneous occurrence difficult.

##### Notes

This species was reported as occurring in large populated places of Orenburg Region (Iljin 1930, Ryabinina 1998, Knjazev 2009), but no specimens were seen. Nevertheless, it may be found as cultivated in gardens as an ornamental plant.

#### Atriplex rosea

L.

7B1743FA-30D2-5A4B-977D-B926990DA905

##### Distribution

It has disappeared from almost all territories of European Russia where it was collected in the 19^th^ and first third of 20^th^ centuries and all old specimens were collected in the areas located westwards of the Volga (Sukhorukov 2006, Sukhorukov et al. 2022), thus outside of Orenburg Region.

##### Notes

Reported by Ryabinina (1998) and Knjazev (2009). No specimens seen.

#### Chenopodium acuminatum

Willd.

75C7C246-CFDA-5091-B74C-0136B3FF96F0

##### Distribution

A species widely distributed from Central Kazakhstan, South Siberia, Central Asia and temperate Far East.

##### Notes

Previously reported by Ryabinina (1998) and Knjazev (2009).

All specimens seen from Orenburg Region belong to *Chenopodiumalbum* L. or *C.betaceum* Andrz. Nevertheless, future records are possible due to its presence in the neighbouring Kurgan Region (NSK!, SVER!).

#### Chenopodium karoi

(Murr) Aellen

C06F6B9B-5FCD-5199-A8F9-4A7EC54EA91C

##### Distribution

It is distributed in temperate Asia and often confused with other species (Lomonosova and Uotila 2022). In the last three decades, *C.karoi* was found as an alien plant along railroad embankments in the neighbouring Bashkortostan Republic (SVER!).

##### Notes

Knjazev (2009) reported this species as a rare alien occurring on railway embankments in western districts of Orenburg Region. No specimens were seen.

#### Chenopodium praetericola

Rydb.

9F0080B9-6D2F-57D0-96A7-F0D0ECA3445D

##### Distribution

A North American species often confused with the native narrow-leaved *Chenopodiumalbum* and *C.betaceum*. Its presence in European Russia is still doubtful. Identifications can be proven by measurements of the seed coat thickness (Sukhorukov and Zhang 2013, Sukhorukov 2014) and molecular phylogeny.

##### Notes

Knjazev (2009) reported this alien species from the Guberlya Mts. No specimens were seen to confirm this record.

#### Chenopodium suecicum

Murr.

C0B8B2BE-FDDA-567C-BA2C-AB4F75CCC1D5

##### Distribution

Northern Eurasia.

##### Notes

Ryabinina (1998) reported this segregate of the *Chenopodiumalbum* L. aggr. as occurring on ruderal places in the whole Orenburg Region. While the species occurrence is possible in this territory, no herbarium specimens were found to confirm this record.

#### Chenopodium vulvaria

L.

EFC8B97D-E094-5B61-A6F6-4789C16B26BE

##### Distribution

Mediterranean, West Asia, Iran, southern Central Asia, Himalayas (Groom 2015, Lomonosova and Uotila 2022).

##### Notes

This species was reported as a rare plant possibly occurring in the western part of Orenburg Region (Knjazev 2009), although it has been previously believed to occur throughout the whole territory (Ryabinina 1998). Some voucher specimens seen in ORIS belong to the *Chenopodiumalbum* aggregate and no specimens of *C.vulvaria* from the entire Volga-Ural region were seen by the first author.

#### Climacoptera affinis

(C.A.Mey. ex Schrenk) Botsch.

390B1D70-48BA-59FB-A23E-8906C0769D69

##### Distribution

Central Asia.

##### Notes

The species was once collected by Antonov in 1852 “near Orsk town” (LE). More recent collections are absent from Orenburg Region. The species is widely distributed in central and eastern Kazakhstan.

#### Climacoptera crassa

(M.Bieb.) Botsch.

B701EC13-F635-5576-8DF7-EF01A65D1524

##### Distribution

Central Asia.

##### Notes

Iljin (1930) reported this species from Orenburg on the basis of a single old specimen collected by Karelin in the mid-19^th^ century. This occurrence has not been confirmed by recent specimens. The northern limit of this species distribution area lies south of Orenburg Region, in the southern part of Aqtöbe Region of Kazakhstan.

#### Halimocnemis karelinii

Moq.

40F98A0C-98E4-58BC-893E-D0BAAB345123

##### Distribution

Central Asia.

##### Notes

There is one old collection from Orenburg Region with the label that reads “Orsk, 1852, Antonov” (LE). We believe that this specimen was collected south of Orenburg Region, apparently in Aqtöbe Region of Kazakhstan. The species is common in southern Kazakhstan.

#### Petrosimonia oppositifolia

(Pall.) Litv.

C53AC0EE-DFAB-578F-A42C-4F952607C313

##### Distribution

Eastern and South-Eastern Europe, Central Asia.

##### Notes

Ryabinina (1998) reported this species from four districts of Orenburg Region. However, voucher specimens are absent in herbarium collections. Although the presence of this species is logically possible in the Region, we exclude it from the list due to the lack of documentation.

#### Salsola paulsenii

Litv.

D5EB6C9B-84D7-5AA8-8281-2B0839FE435F

##### Distribution

SE Europe (Astrakhan Region, Kalmyk Republic), Central Asia, Afghanistan, Iran, NW China.

##### Notes

Collected by Antonov in 1852 “near Orsk town”, but this record was not confirmed by later specimens. At that time, Orenburg Region was a very remote location and one of the outposts of the Russian Empire. The exact location of these records cannot be properly established and the species was seemingly collected by Antonov in northern Kazakhstan.

#### Xylosalsola arbuscula

(Pall.) Tzvel.

B0456154-002B-5721-A217-8454F90EA1BB

##### Distribution

SE Europe (Astrakhan and Volgograd Regions), Central Asia.

##### Notes

Collected by Antonov in 1852 “near Orsk town” (LE), but not confirmed by later gatherings. The nearest localities are known in Central Kazakhstan. So far, this conspicuous plant has not been observed during floristic work in Orenburg Region (Ryabinina 1998, Knjazev 2009).

## Discussion

### Data collection and species distributions

So far, our sampling is the most comprehensive attempt of data collection for Chenopodiaceae in Orenburg Region. Regarding less common species, especially those currently under legal protection, our distribution maps demonstrate that the coverage increased substantially due to the recent collection effort and the inclusion of all available herbarium specimens, especially those kept in smaller and local collections.

At the same time, we acknowledge that our current data remain partly deficient because of still insufficient sampling, especially of common and taxonomically critical taxa. Recent accounts (Ryabinina 1998, Knjazev 2009) assume broader distributions of some species; however, we were not able to verify those distributional data because of the lack of vouchers.

Although Chenopodiaceae are characteristic of arid zones as a whole, several species of this family occur at their zonal limits of distribution in Orenburg Region. For example, *Atriplexoblongifolia*, *A.patens*, *Ceratocarpusarenarius*, *Krascheninnikoviaceratoides*, *Salicorniaperennans*, *Suaedaacuminata*, *S.corniculata*, *Bassiaprostrata*, *Camphorosmalessingii*, *C.monspeliaca* etc. are characteristic of the northern steppes, whereas the northern limits of *Agriophyllumpungens*, *Halocnemumstrobilaceum*, *Kalidiumcaspicum*, *K.foliatum*, *Bassiahyssopifolia*, *B.laniflora*, *Spirobassiahirsuta*, *Ofaistonmonandrum*, *Petrosimoniabrachyphylla*, *Anabasissalsa*, *Pyankoviabrachiata*, *Arthrophytumlehmannianum*, *Halogetonglomeratus* etc. largely belong to the true steppes of Orenburg Region and the semi-deserts of northern Kazakhstan. Such patterns were used in phytogeographic studies by Krascheninnikov (1925) and revealed in Chenopodiaceae by Iljin (1930). We confirm the significance of zonal patterns in Chenopodiaceae using a denser geographical sampling, although we also observed that these patterns are partly blurred in Orenburg Region. For example, typical dominant plants of semi-deserts, *Atriplexcana* and *Suaedaphysophora*, extensively penetrate into true steppes, but occur sporadically there, in smaller populations.

### Species richness

To date, 76 species of Chenopodiaceae are confirmed to occur in Orenburg Region (Table 1). This number is much higher than in the neighbouring administrative territories (Bashkortostan Republic, Kurgan, Rostov, Samara, Saratov Regions), is comparable to the count in Astrakhan Region and slightly lower than those in Volgograd Region and Dagestan Republic. The territories situated in the forest zone are not included in the comparisons due to the drastically decreasing species numbers.

### Conservation

Although most of the Chenopodiaceae species in Orenburg Region are either common or confined to the habitats which are not exploited by humans (e.g. saline substrates), some of these species may be rare and threatened because of being situated at the margin of their distribution or because of being restricted to the habitats intensely used (e.g. calcareous deposits).

Two species (*Anabasiscretacea*, *Nanophytonerinaceum*) occur mostly on calcareous denudations, which can be destroyed by mining in the absence of protection; they are protected starting from the first edition of the regional Red Data Book. Two species (*Anabasissalsa* and *Kalidiumfoliatum*) are currently protected, because their populations are situated on the margin of their distribution. We propose adding two more species, *Arthrophytumlehmannianum* and *Salsolarosacea*, to the same category because their populations in Orenburg Region are small and situated outside the main area as the northernmost or westernmost foreposts of the species distributions.

We recommend to exclude *Petrosimoniatriandra* from the Red Data Book of Orenburg Region, because the actual distribution of this species, as circumscribed in our study, was found much greater than previously assumed and no apparent reduction in its population size or occurrence was observed or projected. Similarly, *Kalidiumfoliatum* and *Anabasissalsa* are proposed for exclusion because their populations are situated in saline lands, which are not threatened by anthropogenic disturbance.

As a general observation, the conservation status of Chenopodiaceae species in Orenburg Region depends on plant habitats. We do not consider species occurring on saline lands (even those with restricted distributions) as threatened because this habitat is not used in economic activities and is not under immediate threat of destruction or significant degradation. On the other hand, calcareous outcrops are the traditional target of mining for construction works and plants restricted to this habitat may be threatened due to the loss of habitats. Plants occurring in petrophytic steppes, although these habitats are outside of particular economic interest, may also be threatened because their populations may be situated near roads and may be destroyed by the expansion of the road network or because of grazing by domestic livestock. The primary importance of habitat threatening was underestimated in the previous work on plant conservation in Orenburg Region (Belov 2019), which focused more on plant rarity and, therefore, arrived at different conclusions.

### Vegetation features

**Xerophilous grasslands, or temperate steppe**

Apart from Poaceae and many Asteraceae (mostly represented by *Artemisia* spp.), subshrubby *Bassiaprostrata* and *Krascheninnikoviaceratodes* (Fig. 79b) dominate in this type of natural community (Fig. 80a), sometimes with *Caroxylonlaricinum*. Depending on different degrees of steppe disturbance, the annual Chenopodiaceae (*Atriplexoblongifolia*, *A.sagittata*, *Ceratocarpusarenarius*, *Chenopodiumalbum*, *Oxybasisurbica*, *Sedobassiasedoides*, *Salsolatamariscina*) can be more or less abundant in grasslands or form patches of different origin, for example, influenced by the activities of *Marmotabobak*, erosion or human-based activities. According to our observations, some other true steppe annuals like *Atriplexsphaeromorpha* and *Petrosimoniamonandra* are very rare in Orenburg Region and much more frequent in steppes and semi-deserts of Kazakhstan.

**Petrophytic steppe**

This steppe subtype (Fig. 80b) is locally distributed in the central, western and south-eastern parts of Orenburg Region. Due to an increased salt content in the soil, uneven microrelief and stony substrate, petrophytic steppe is unsuitable for agriculture or contains plants not eaten by livestock (e.g. many Chenopodiaceae: Iljin (1936)). For these reasons, petrophytic steppes are usually less degraded compared with other open plant communities. Among other widespread plants, for example *Alyssumtortuosum* (Brassicaceae), *Artemisialerchiana* (Asteraceae), *Limoniumsuffruticosum* (Plumbaginaceae) and *Scabiosaisetensis* (Caprifoliaceae), the Chenopodiaceae are represented by the subshrubby *Nanophytonerinaceum*, as well as by *Anabasiscretacea* and *Atriplexcana*, both latter species being more typical of other natural vegetation types. Another subshrubby petrophytic species, *Arthrophytumlehmannianum*, was found only recently as a single small population (Stepanova and Safronova 2019). The annuals *Salsolacollina* and *S.tamariscina* (Fig. 79c) are also common in petrophytic communities.

**Halophytic plant communities**

Halophytic plant communities (Fig. 80c) are represented by ‘solonetz’ and ‘solonchak’ communities, differing in the level of salinity. They are very common in the southern and eastern parts of Orenburg Region. Nevertheless, the subshrubby chenopodiaceous plants are unevenly distributed across the territory. *Kalidiumfoliatum*, *Atriplexlaevis* (Fig. 79a) and *Suaedalinifolia* (Fig. 79e) are restricted to its south-eastern part (solonchaks on dried-up salt lakes and ponds), *Suaedaphysophora* is distributed in the eastern part, *Atriplexcana* and *Anabasissalsa* are stretching through the south and east and *Halimioneverrucifera* is found across the territory. All of them are at the northern limit of distribution. Other subshrubs (*Camphorosma* spp.) and the annuals *Atriplexpatens*, *A.tatarica*, *Camphorosmasongorica*, *Oxybasischenopodioides*, *Petrosimonialitvinovii*, *P.triandra*, *Salicorniaperennans*, *Suaeda* spp. and *Sedobassiasedoides* are common elements of diverse halophytic communities. In general, the role of the subshrubby Chenopodiaceae increases from the north towards the south and east.

**Limestone and chalk formations**

Calcareous outcrops (Fig. 80d) are locally found in the territory, especially in its southern part. *Anabasiscretacea* (Fig. 79d) and *Nanophytonerinaceum* (Fig. 79f) are characteristic of chalk hills (Golovanov et al. 2021); the subshrubby *Bassiaprostrata, Krascheninnikoviaceratoides and Caroxylonlaricinum* and the annuals *Blitumvirgatum*, *Salsolatamariscina* and *Sodafoliosa* often occur in both chalk and limestone formations.

**Sands**

Psammophyte communities (Fig. 80e) are not uniform in their species composition. Riverbank soil ecosystems, particularly along the Ural River, the major watercourse in the region, include several mesophytic Chenopodiaceae (*Atriplexprostrata*, *Chenopodiumacerifolium*, *Corispermumsquarrosum*, *Oxybasisrubra*, *O.glauca*), but some of them (*Chenopodiumacerifolium*, *Corispermumsquarrosum*) are undercollected and considered rare in the territory. Sandy steppe is another type of the psammophytic vegetation. This kind of steppes or meadows is xerophytic and contains other chenopodiaceous species (native *Bassialaniflora*, *Corispermumhyssopifolium*, *Salsolacollina*, *Salsolatragus*, alien *Corispermumdeclinatum*), as well as the rare native *Agriophyllumpungens* in the south. Recently, *Chenopodiumvirgatum* was discovered in degraded sandy steppe communities.

**Disturbed lands**

Some annual Chenopodiaceae are noxious ruderal plants. *Atriplexsagittata*, *Chenopodiumalbum*, *Chenopodiastrumhybridum*, *Oxybasisurbica* and *Salsolacollina* are the most common species along roadsides (Fig. 80f), degraded fluvial terraces and other dump places. *Atriplexmicrantha*, with scattered occurrences in halophytic communities, was recently found in ruderal plant communities (collected by APS in Orenburg City in 2021). *Atriplexpatula*, one of the most common ruderal plants in the forest-steppe and broad-leaved forest subzones, is not frequently found in Orenburg Region.

## Supplementary Material

XML Treatment for Blitum virgatum

XML Treatment for Teloxys aristata

XML Treatment for Dysphania botrys

XML Treatment for Atriplex aucheri

XML Treatment for Atriplex cana

XML Treatment for Atriplex intracontinentalis

XML Treatment for Atriplex laevis

XML Treatment for Atriplex micrantha

XML Treatment for Atriplex oblongifolia

XML Treatment for Atriplex patens

XML Treatment for Atriplex patula

XML Treatment for Atriplex prostrata

XML Treatment for Atriplex sagittata

XML Treatment for Atriplex sphaeromorpha

XML Treatment for Atriplex tatarica

XML Treatment for Chenopodium acerifolium

XML Treatment for Chenopodium album

XML Treatment for Chenopodium betaceum

XML Treatment for Chenopodium opulifolium

XML Treatment for Chenopodium virgatum

XML Treatment for Chenopodiastrum hybridum

XML Treatment for Halimione pedunculata

XML Treatment for Halimione verrucifera

XML Treatment for Lipandra polysperma

XML Treatment for Oxybasis chenopodioides

XML Treatment for Oxybasis glauca

XML Treatment for Oxybasis rubra

XML Treatment for Oxybasis urbica

XML Treatment for Axyris amaranthoides

XML Treatment for Ceratocarpus arenarius

XML Treatment for Krascheninnikovia ceratoides

XML Treatment for Agriophyllum pungens

XML Treatment for Corispermum declinatum

XML Treatment for Corispermum hyssopifolium

XML Treatment for Corispermum laxiflorum

XML Treatment for Corispermum marschallii

XML Treatment for Corispermum squarrosum

XML Treatment for Halocnemum strobilaceum

XML Treatment for Kalidium caspicum

XML Treatment for Kalidium foliatum

XML Treatment for Salicornia perennans

XML Treatment for Suaeda acuminata

XML Treatment for Suaeda corniculata

XML Treatment for Suaeda kulundensis

XML Treatment for Suaeda linifolia

XML Treatment for Suaeda physophora

XML Treatment for Suaeda prostrata

XML Treatment for Suaeda salsa

XML Treatment for Bassia hyssopifolia

XML Treatment for Bassia laniflora

XML Treatment for Bassia prostrata

XML Treatment for Bassia scoparia

XML Treatment for Camphorosma lessingii

XML Treatment for Camphorosma monspeliaca

XML Treatment for Camphorosma songorica

XML Treatment for Sedobassia sedoides

XML Treatment for Spirobassia hirsuta

XML Treatment for Caroxylon laricinum

XML Treatment for Nanophyton erinaceum

XML Treatment for Ofaiston monandrum

XML Treatment for Petrosimonia brachyphylla

XML Treatment for Petrosimonia litwinowii

XML Treatment for Petrosimonia monandra

XML Treatment for Petrosimonia triandra

XML Treatment for Pyankovia brachiata

XML Treatment for Anabasis cretacea

XML Treatment for Anabasis salsa

XML Treatment for Arthrophytum lehmannianum

XML Treatment for Halogeton glomeratus

XML Treatment for Salsola collina

XML Treatment for Salsola rosacea

XML Treatment for Salsola tamariscina

XML Treatment for Salsola tragus

XML Treatment for Soda acutifolia

XML Treatment for Soda foliosa

XML Treatment for Soda inermis

XML Treatment for Atriplex hortensis

XML Treatment for Atriplex rosea

XML Treatment for Chenopodium acuminatum

XML Treatment for Chenopodium karoi

XML Treatment for Chenopodium praetericola

XML Treatment for Chenopodium suecicum

XML Treatment for Chenopodium vulvaria

XML Treatment for Climacoptera affinis

XML Treatment for Climacoptera crassa

XML Treatment for Halimocnemis karelinii

XML Treatment for Petrosimonia oppositifolia

XML Treatment for Salsola paulsenii

XML Treatment for Xylosalsola arbuscula

FD26CFBA-E430-5A44-978E-E7EFB6B4D210 10.3897/BDJ.12.e121541.suppl1 Supplementary material 1 Voucher specimens for the revision of Chenopodiaceae in Orenburg Region, Russia Data type occurrences Brief description Occurrence dataset based on herbarium specimens and human observations, formatted according to DarwinCore standard. File: oo_977667.txt https://binary.pensoft.net/file/977667 Sukhorukov, A.P.

## Figures and Tables

**Figure 79a.**
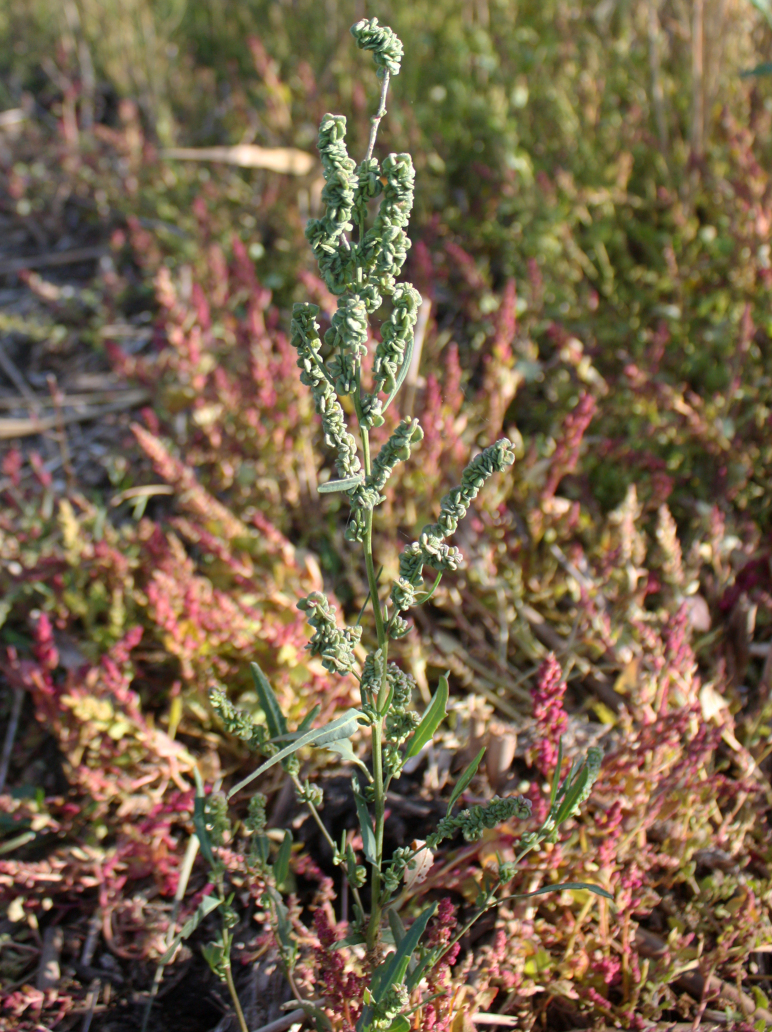
*Atriplexlaevis* (Svetlyi District, 25.09.2020, A. Sukhorukov).

**Figure 79b.**
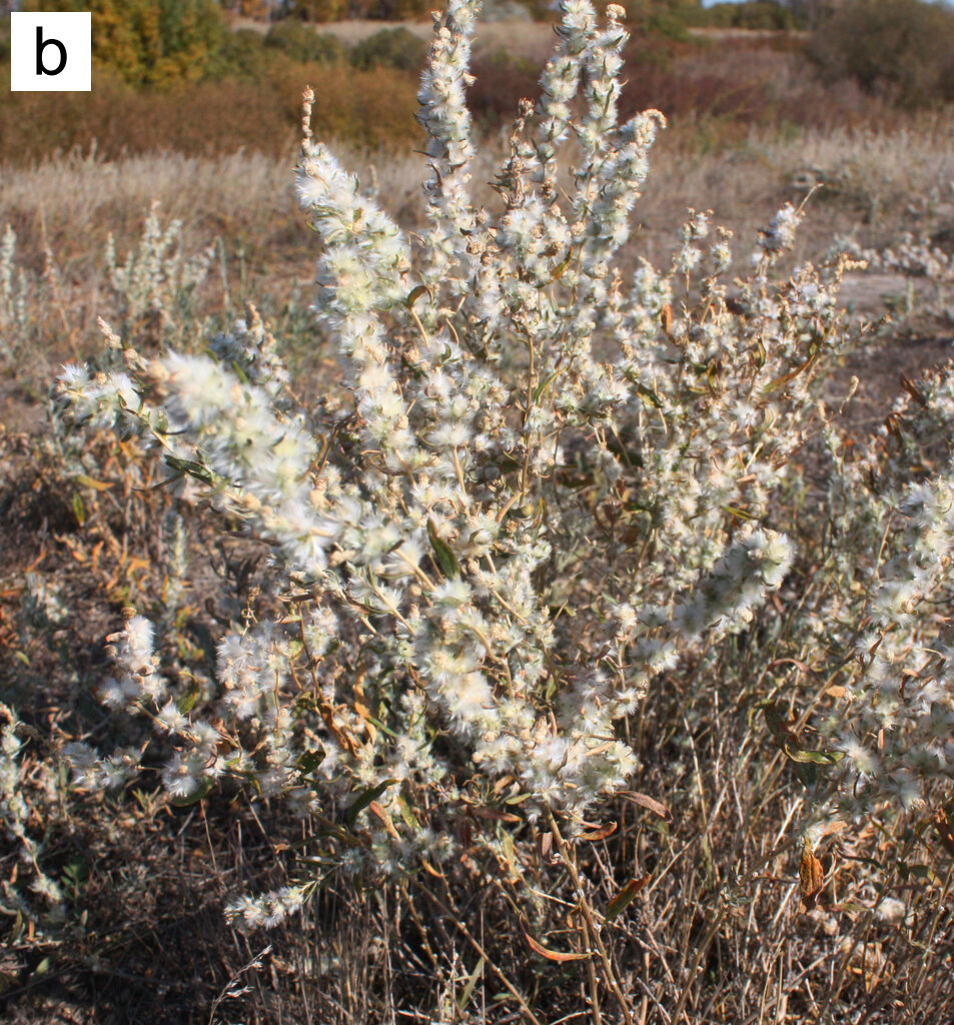
*Krascheninnikoviaceratoides* (Adamovka District, 27.09.2020, A. Sukhorukov).

**Figure 79c.**
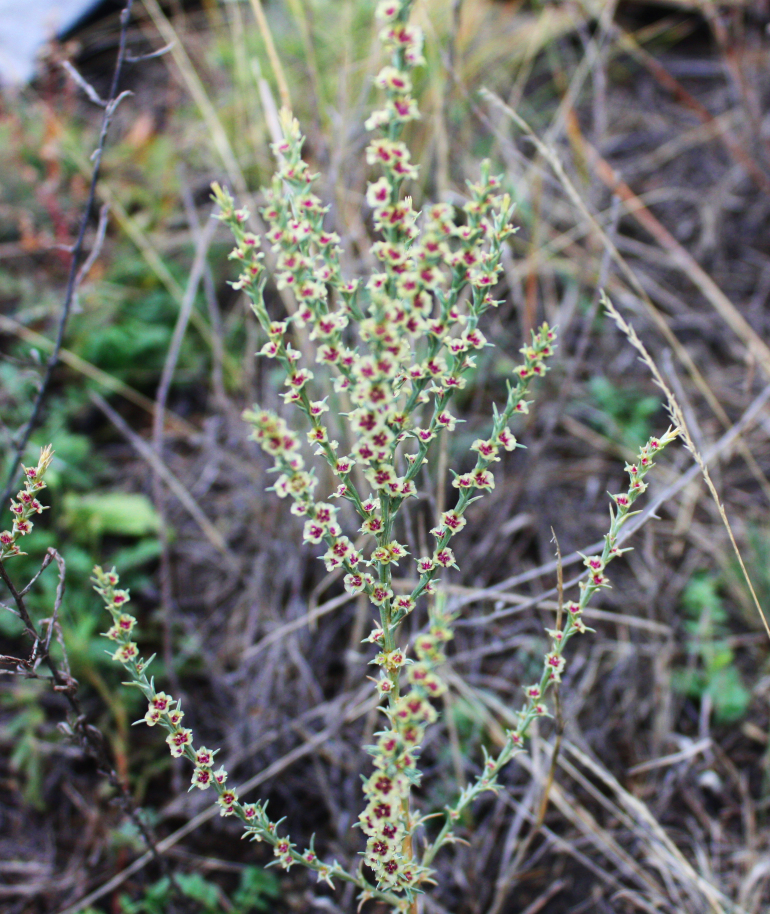
*Salsolatamariscina* (Buguruslan District, 21.09.2020, A. Sukhorukov).

**Figure 79d.**
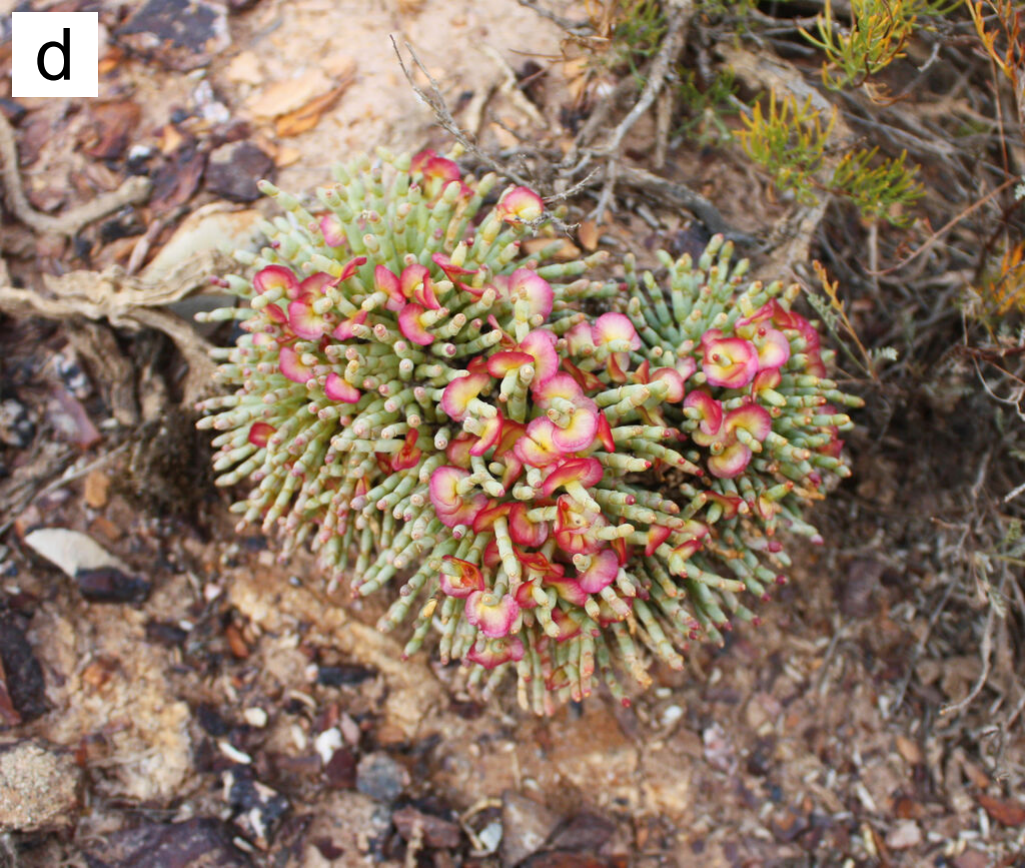
*Anabasiscretacea* (Yasnyi District, 22.09.2020, A. Sukhorukov).

**Figure 79e.**
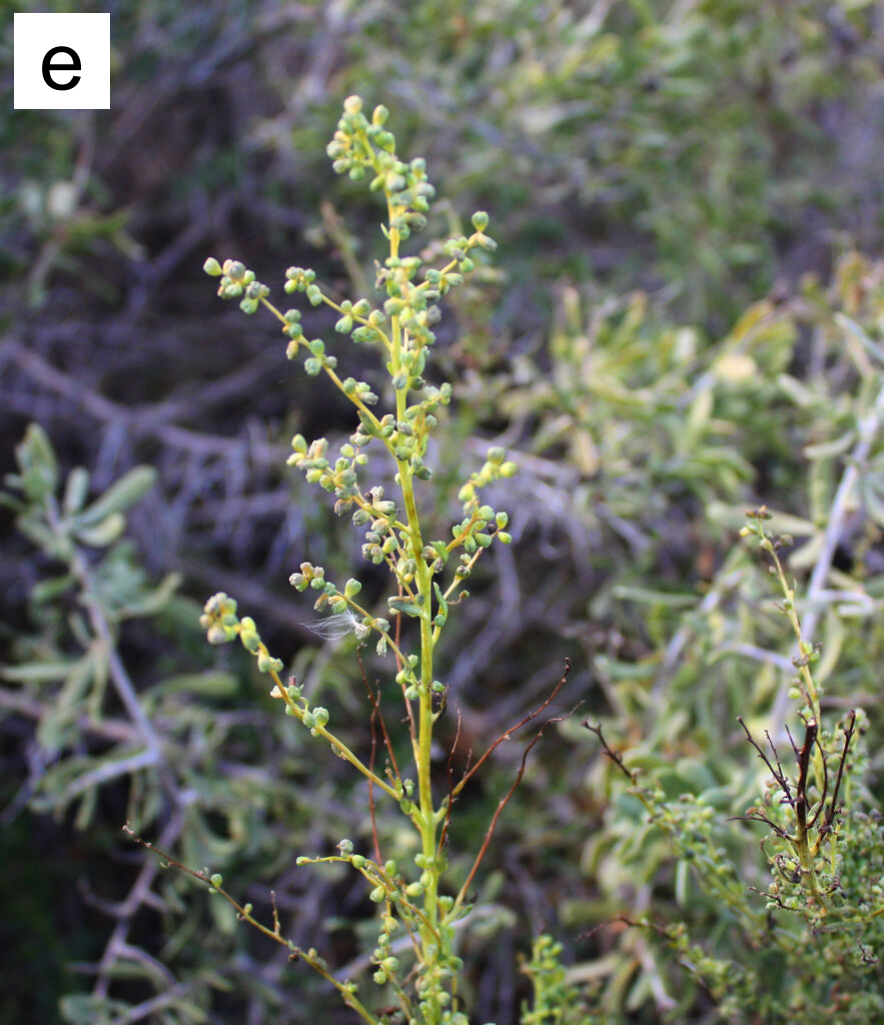
*Suaedalinifolia* (Svetlyi District, 25.09.2020, A. Sukhorukov).

**Figure 79f.**
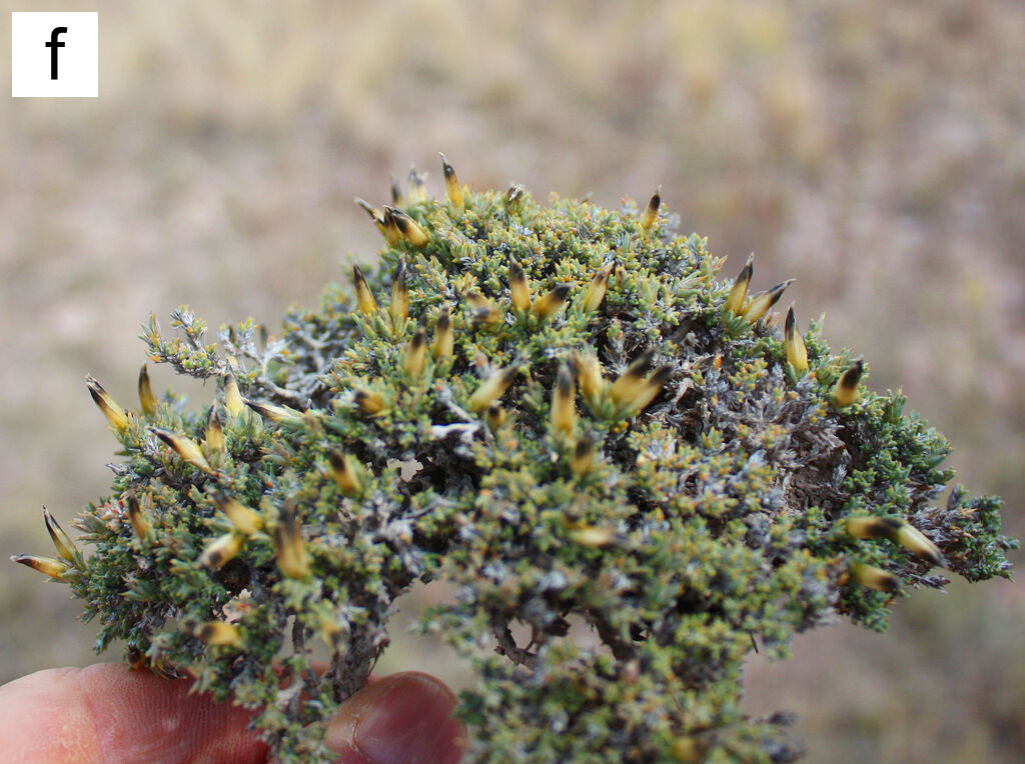
*Nanophytonerinaceum* (Yasnyi District, 22.09.2020, A. Sukhorukov).

**Figure 80a.**
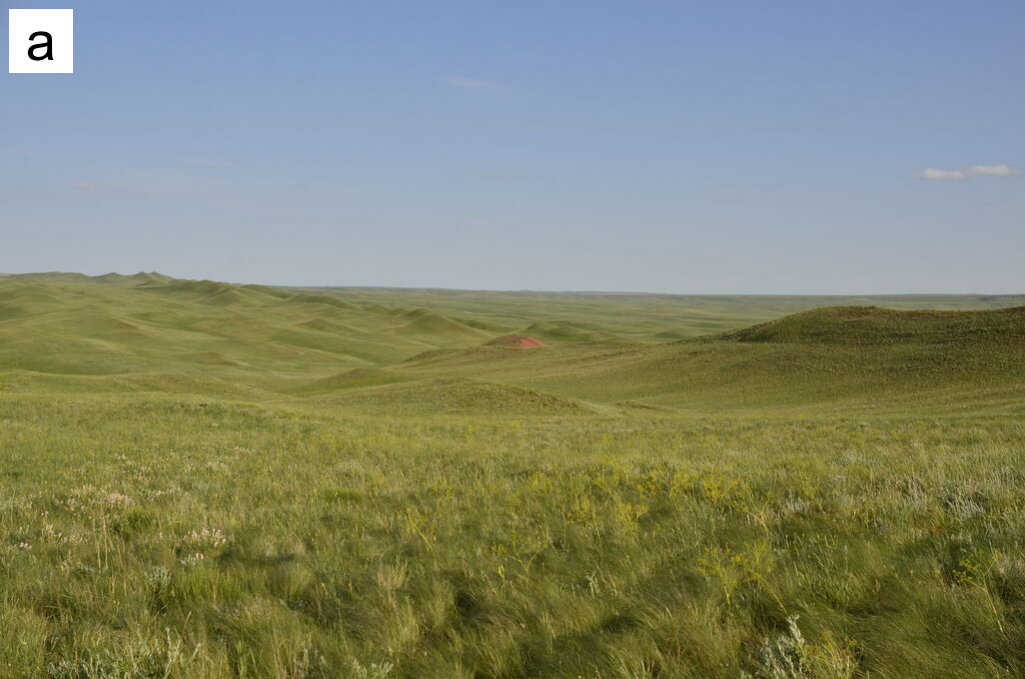
Steppe (Kuvandyk District, 08.06.2015, Ya. Golovanov).

**Figure 80b.**
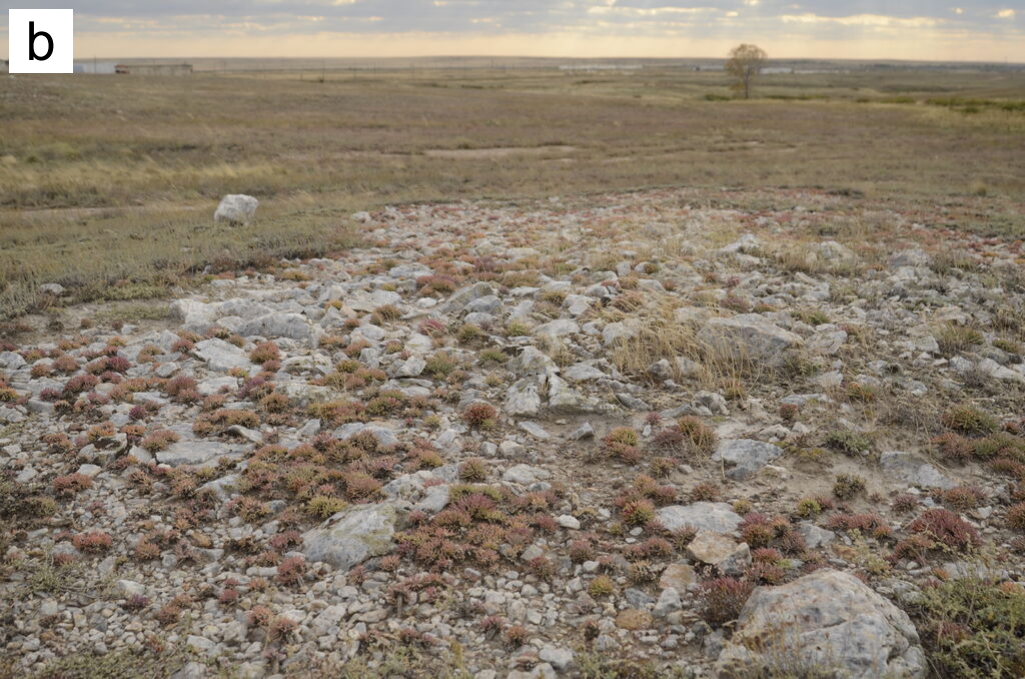
Petrophytic steppe (Yasnyi District, 23.09.2020, N. Stepanova).

**Figure 80c.**
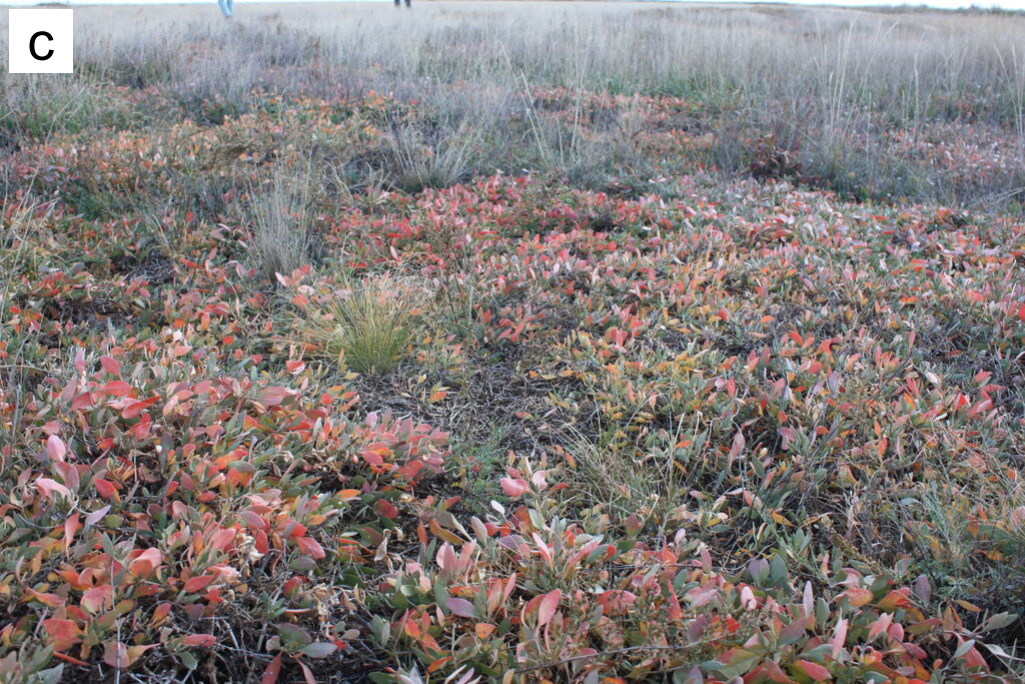
Saline lands with *Halimioneverrucifera* as a dominant (Yasnyi District, 25.09.2020, A. Sukhorukov).

**Figure 80d.**
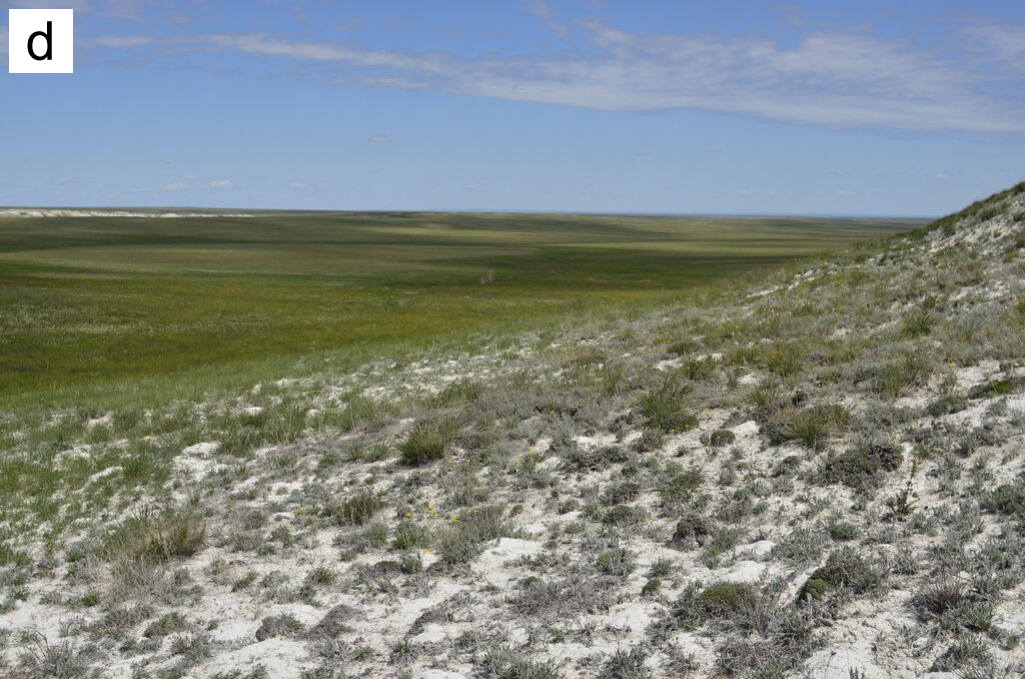
Calcareous outcrops (Sol'-Iletsk District, 07.06.2018, Ya. Golovanov).

**Figure 80e.**
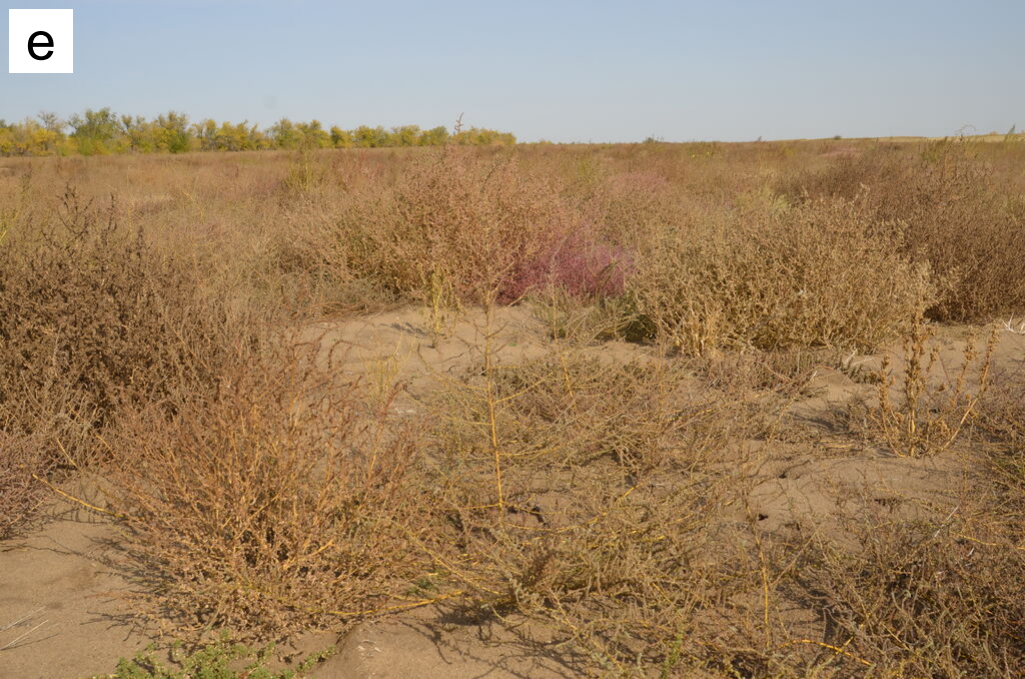
Sands (Sol'-Iletsk District, 28.09.2020, N. Stepanova).

**Figure 80f.**
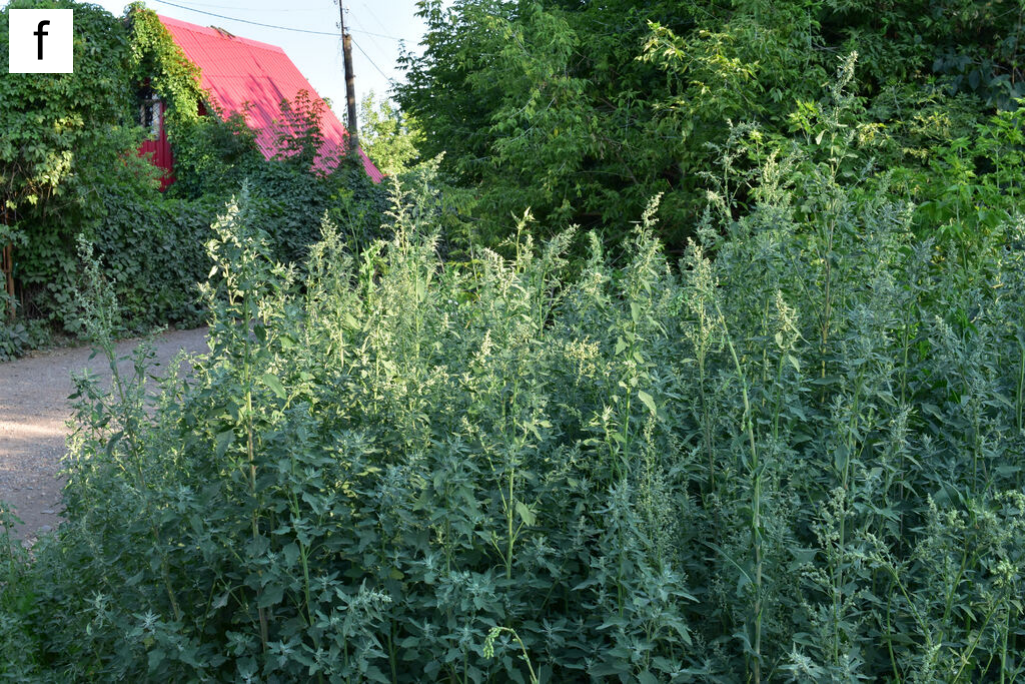
Ruderal vegetation (Orenburg City, 09.07.2020, Ya. Golovanov).

**Table 1.** Species richness of Chenopodiaceae in Orenburg Region and other southern administrative territories of Russia.

Administrative territory	Species number	Source
Astrakhan Region	75	Sukhorukov (unpubl. data)
Republic of Bashkortostan	57	Muldashev et al. (2017)
Chelyabinsk Region	62	Kulikov (2010)
Republic of Dagestan	82	Murtazaliev (2009)
Kurgan Region	44	Naumenko (2008)
Orenburg Region	76	Present study
Samara Region	45	Sukhorukov (unpubl. data)
Saratov Region	62	Sukhorukov (unpubl. data)
Rostov Region	53	Sukhorukov (unpubl. data)
Volgograd Region	80	Sukhorukov (unpubl. data)
